# EPISCORE: cell type deconvolution of bulk tissue DNA methylomes from single-cell RNA-Seq data

**DOI:** 10.1186/s13059-020-02126-9

**Published:** 2020-09-04

**Authors:** Andrew E. Teschendorff, Tianyu Zhu, Charles E. Breeze, Stephan Beck

**Affiliations:** 1 CAS Key Laboratory of Computational Biology, CAS-MPG Partner Institute for Computational Biology, Shanghai Institute of Nutrition and Health, Shanghai Institutes for Biological Sciences, University of Chinese Academy of Sciences, Chinese Academy of Sciences, 320 Yue Yang Road, Shanghai, 200031 China; 2 UCL Cancer Institute, Paul O’Gorman Building, University College London, 72 Huntley Street, London, WC1E 6BT UK; 3 Altius Institute for Biomedical Sciences, 2211 Elliott Avenue, Seattle, USA

**Keywords:** DNA methylation, EWAS, Single-cell RNA-Seq

## Abstract

Cell type heterogeneity presents a challenge to the interpretation of epigenome data, compounded by the difficulty in generating reliable single-cell DNA methylomes for large numbers of cells and samples. We present EPISCORE, a computational algorithm that performs virtual microdissection of bulk tissue DNA methylation data at single cell-type resolution for any solid tissue. EPISCORE applies a probabilistic epigenetic model of gene regulation to a single-cell RNA-seq tissue atlas to generate a tissue-specific DNA methylation reference matrix, allowing quantification of cell-type proportions and cell-type-specific differential methylation signals in bulk tissue data. We validate EPISCORE in multiple epigenome studies and tissue types.

## Background

DNA methylation is a key cell type-specific epigenetic mark associated with gene expression that plays a key role in development and differentiation [1]. Epigenome-wide association studies (EWAS) have demonstrated altered DNA methylation (DNAm) patterns in a wide range of diseases [2, 3], but interpretation is severely hampered by cell type heterogeneity [4–7]. Indeed, due to cost and logistical reasons, almost all of the genome-wide DNA profiles generated to date have been performed in complex tissues that are composed of many different cell types, which can confound analysis and prevent the identification of cell type-specific changes underlying disease. In principle, the challenge posed by cell type heterogeneity is best addressed with single-cell technologies [8, 9], which consortia such as the Human and Mouse Cell Atlas (HCA/MCA) projects [10–13] are using to generate tissue-specific single-cell RNA-Seq (scRNA-Seq) atlases at high cellular resolution. Such tissue-specific scRNA-Seq atlases provide a nearly unbiased catalog of all major cell types present in a tissue and constitute a resource that is already being exploited to enable cell type deconvolution of bulk mRNA expression profiles [14]. However, the generation of single-cell atlases at the DNAm level is currently infeasible for most tissues because current single-cell DNAm technologies only generate very sparse data, in relatively low numbers of cells/samples, and at high cost [9, 15–17]. Hence, there is a pressing need to develop orthogonal, computational, solutions for performing cell type deconvolution of bulk tissue epigenetic data at single-cell type resolution.

Here, we asked if the high resolution nature of tissue-specific scRNA-Seq atlases can be leveraged to generate corresponding tissue-specific DNAm reference matrices, which, in conjunction with existing reference-based cell type deconvolution algorithms [6, 14, 18–23], would enable cell type deconvolution of an arbitrary bulk tissue DNAm profile. To this end, we develop a novel computational algorithm called EPISCORE (Epigenetic cell type deconvolution using Single Cell Omic REferences), which can infer DNAm levels at regulatory elements of cell type-specific marker genes, thus allowing translation of a tissue-specific scRNA-Seq reference matrix into a corresponding one at the DNAm level. The resulting tissue-specific DNAm reference matrix can subsequently be used to estimate the cellular composition of a corresponding bulk tissue DNAm sample, and to identify differentially methylated cell types (DMCTs) [24], i.e., cell type-specific differentially methylated cytosines, within the context of any given EWAS or general epigenome study. Thus, EPISCORE enables cell type deconvolution at high cellular resolution of the many thousands of bulk DNAm profiles from solid tissues that are present in the public domain [25, 26], which would otherwise remain fully confounded by cell type heterogeneity.

## Results

### The EPISCORE algorithm

**Fig. 1:**
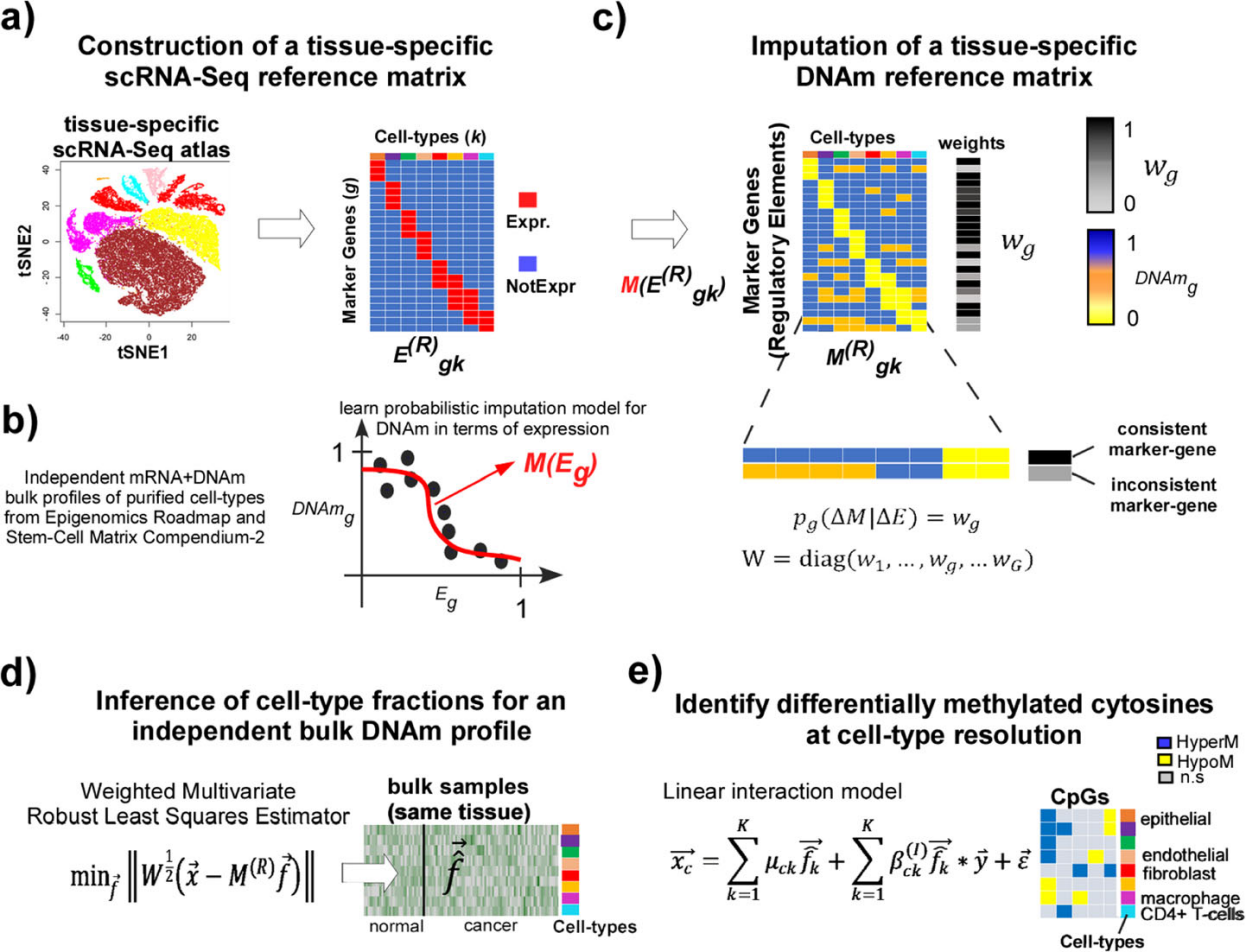
EPISCORE concept and workflow. **a** First step in EPISCORE is the construction of a tissue-specific mRNA expression reference matrix E^(R)^_gk_, which is derived from a corresponding scRNA-Seq tissue atlas. The expression reference matrix is defined over a set of marker genes that are differentially expressed between cell types, as defined in the scRNA-Seq tissue atlas. **b** Using completely independent (matched) bulk RNA-Seq and DNAm data of purified samples from Epigenomics Roadmap and SCM2, we identify genes for which differential DNAm at their regulatory elements (e.g., promoter) across the samples is predicted by corresponding gene expression. For the predictive genes, we learn a probabilistic Bayesian model denoted M (E_g_), using logistic regression fits if necessary that allows prediction of likely DNAm values from gene expression. **c** Using the model learned in **b** and the expression values from the reference matrix constructed in **a**, we impute a corresponding tissue-specific DNAm reference matrix M^(R)^_gk_, weighting the marker genes (w_g_) according to how well the imputed DNAm values reflect gene expression. **d** Using the imputed DNAm reference matrix, we can now estimate proportions for the corresponding cell types (encoded as a vector $\overrightarrow{\mathrm{f}}$ with K elements, one for each cell type) in a bulk DNAm profile $\overrightarrow{\mathrm{x}}$ (encoded as a vector over the CpGs/genes in the DNAm reference matrix) representing the given tissue type, be it healthy or disease. The estimation proceeds via weighted multivariate robust linear least squares that tries to minimize the objective function as shown. **e** With these cell type fraction estimates, it is then possible to generate genome-wide maps of cell type-specific differential DNAm changes at resolution of single CpGs, informing us which CpGs are hyper or hypomethylated in any given cell type in relation to some phenotype of interest. In the equation, ${\overrightarrow{\mathrm{x}}}_{\mathrm{c}}$ denotes the DNA methylation profile of a CpG c across the samples, ${\overrightarrow{\hat{\mathrm{f}}}}_{\mathrm{k}}$ is the estimated cell type fraction for cell type k across the samples, and $\overrightarrow{\mathrm{y}}$ denotes the phenotype-label (e.g., normal/cancer) of the samples

We posited that the high-resolution nature of tissue-specific scRNA-Seq atlases can be leveraged to construct approximate tissue-specific reference profiles at the DNAm level, via development of a probabilistic model that infers likely DNAm values at differentially expressed marker genes within each of the cell types in the given tissue. Briefly, EPISCORE [27] consists of the following 4 steps (Fig. 1): (i) construction of a tissue-specific mRNA expression reference matrix, defined over a number of marker genes and cell types, as derived from a corresponding tissue-specific scRNA-Seq atlas; (ii) separately from (i), genome-wide identification of genes for which DNAm at their regulatory elements (promoter and/or enhancer) can be predicted from their expression-level; (iii) development of a probabilistic “imputation” model that allows translation of the mRNA expression reference matrix from step (i) into a corresponding DNAm reference matrix; and (iv) estimation of cell type fractions and cell type-specific differential DNAm in corresponding bulk tissue DNAm profiles, using the DNAm reference matrix constructed in step (iii).

### Construction and validation of a lung-specific mRNA expression reference

**Fig. 2:**
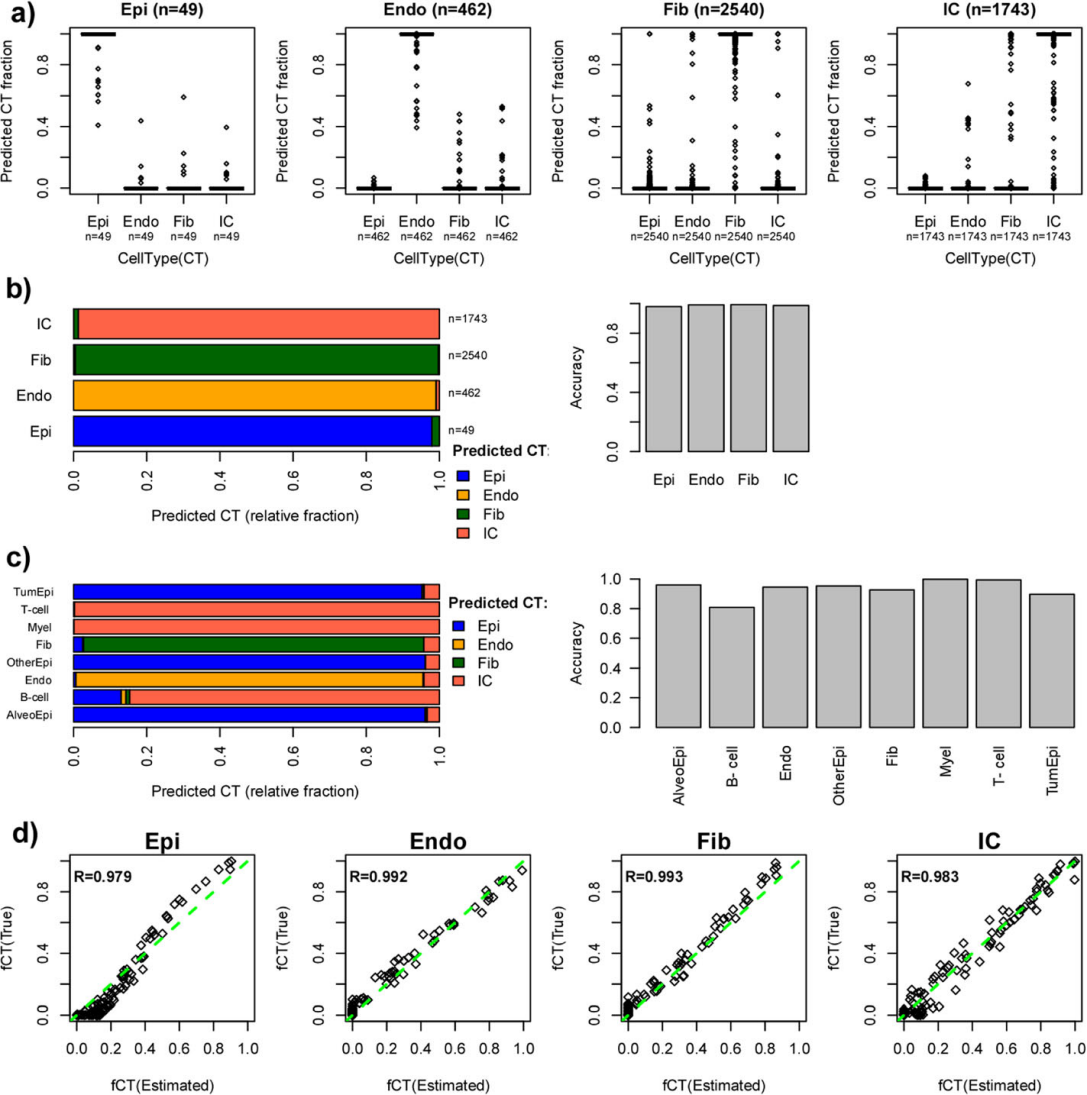
Validation of lung tissue scRNA-Seq reference matrix. **a** Boxplots of estimated cell type fractions for single-cells from the lung MCA1 10X atlas, which were annotated as epithelial (Epi), endothelial (Endo), fibroblast (Fib), and immune cell (IC). The number of single cells annotated to each cell type is given. Cell type fractions were estimated using RPC with an expression reference defined over 4 cell types (epithelial, endothelial, fibroblast, and immune cell) using the SmartSeq2 scRNA-Seq data from MCA1. **b** Left: Barplots depicting the relative fractions of single-cells annotated to each single-cluster (labeled on *y*-axis) which were predicted to be epithelial, endothelial, fibroblast, or immune cell (labeled by different colors) based on the cell type fraction estimates obtained in **a**. Right: Barplot displaying the overall accuracy of using RPC+expression reference to correctly classify each single-cell type. **c** As **b**, but now for using RPC+expression reference on the 10X human data from Lambrecht et al. and for a total 8 cell types as determined in that study. **d** Scatterplots of the true cell type fractions (*y*-axis) vs. the estimated cell type fractions (*x*-axis) in Lambrecht et al. data as obtained via RPC+expression reference on simulated bulk lung tissue samples, generated via in silico mixtures of 1000 single cells. Mixtures were generated from all 8 cell types as shown in **c**, and estimated cell type fractions were obtained for total epithelial, endothelial, fibroblast, and immune cell fractions, as shown

Since EPISCORE is primarily aimed at dissecting the cellular heterogeneity of complex solid tissues, we first focused on lung, a tissue for which ample scRNA-Seq and DNAm data are available, thus allowing for rigorous validation. Specifically, lung tissue was profiled with two different single-cell technologies (SmartSeq2 and 10X) as part of the Tabula Muris/Mouse Cell Atlas-1 (MCA1) consortium [12], as well as by other independent scRNA-Seq studies [28, 29]. We used the Smart-Seq2 MCA1 data to construct an mRNA expression reference matrix defined over 1293 marker genes and 4 main cell types (epithelial, immune cells, endothelial, and fibroblasts) (the “Methods” section). To demonstrate the robustness and validity of this reference matrix, we combined it with a robust partial correlation (RPC) framework [20, 30] to infer cell type fractions and cell type for independent single cells profiled as part of the MCA1 and Lambrecht et al. [28] 10X-assays (the “Methods” section). Of note, the validation in the MCA1-10X data tests for the effects of single-cell technology (SmartSeq2 vs. 10X), whereas the Lambrecht scRNA-Seq set was generated from human cells, thus allowing us to assess if mouse cell atlas data can be used to generate references applicable to humans. We further note that the Lambrecht 10X data was generated in normal lung tissue from lung cancer patients, allowing us to also assess the effects of malignancy on the accuracy of cell type deconvolution. On the MCA1 10X data, cells annotated as epithelial, endothelial, fibroblast, or immune cell were correctly classified as such with an overall accuracy of 98.7% (Fig. 2a, b). An equally high classification accuracy (94%) was observed in the human Lambrecht et al. dataset, even when considering separate epithelial and immune cell subtypes (Fig 2c). For instance, approximately 90% of tumor epithelial cells were correctly classified as epithelial according to our algorithm (Fig. 2c). We also generated in silico mixtures simulating bulk lung tissue samples of known cell type fractions and used RPC with our derived expression reference to infer these fractions. RPC consistently achieved high *R*^2^ values (Fig. 2d). To explore if increased cellular resolution is possible, we rederived a scRNA-Seq expression reference matrix from the Smart-Seq2 MCA1 dataset, but now including separate lymphocyte (147 cells), monocyte (155 cells), and myeloid (85 cells) components. Validating this higher resolution reference matrix in the MCA1-10X scRNA-Seq dataset revealed that classification accuracy remained over 98% for all cell types except myeloid cells (74% accuracy), possibly due to their lower cell numbers (Additional file 1: Fig. S1). All these results thus demonstrate that our lung-specific scRNA-Seq reference is robust and valid across different scRNA-Seq technologies, that it is applicable to human data despite having been constructed in mouse, and that it can correctly classify healthy and malignant cell types within the tumor environment.

### Validation of EPISCORE in lung tissue

In order to translate the above lung-specific expression reference matrix into a corresponding one at the DNAm level, we first performed a genome-wide scan to identify genes for which DNAm at associated regulatory elements (proximal promoter or distal enhancer) can be reasonably predicted from its expression level (“imputable genes”, the “Methods” section). We reasoned that for specific genes such a prediction may be possible, and that these imputable genes would be enriched for cell type-specific marker genes, such as those making up expression reference matrices. To ascertain the validity of these hypotheses, we assembled two independent datasets with matched gene expression and DNAm profiles, one from the NIH Epigenomics Roadmap (RMAP) [31–33] and another from the Stem Cell Matrix Compendium (SCM2) [34], encompassing a wide variety of pure, or relatively pure, somatic primary samples and cell lines, that would allow us to robustly identify genes where promoter DNAm and gene expression are highly associated (the “Methods” section). By using two separate matched datasets, i.e., RMAP with RNA-Seq and whole-genome bisulfite sequencing (WGBS), and SCM2 with Illumina beadarrays (450k and HT12v3), we are able to assess the robustness of our procedure. The array- and sequencing-based matched sets consisted of 13,290 Entrez gene IDs and 34 samples and 18,651 Entrez gene IDs and 45 samples, respectively (Additional file 2). We identified a total of 1152 (SCM2) and 2174 (RMAP) imputable genes, of which the great majority (81% and 85% for SCM2 and RMAP sets, respectively) exhibited anti-correlation between promoter DNAm and gene expression (the “Methods” section). We observed a strong and highly significant overlap of 516 genes (Monte-Carlo empirical *P* < 10^−5^, normal approximation *P* < 10^−300^, Additional file 1: SI Fig. S2a-b). This strong overlap suggested to us that most of the identified genes were true positives and that any differences between the two analyses owes mainly to the use of different sets of samples. Indeed, of the 1152 anti-correlated genes from SCM2, 892 exhibited significant variation in RMAP, of which 88% also exhibited an anti-correlation (Additional file 1: SI Fig. S2c). To maximize gene coverage, we thus declared the union set of 1152 and 2174 genes, representing 2810 unique anti-correlated genes, as defining a core set of imputable genes. Examples of scatterplots between promoter DNAm and mRNA expression in the two matched sets and for many of the genes in this list clearly support the view that for these core genes, DNAm is predictable from gene expression (Additional file 1: SI Fig. S3). For instance, for *MYO1G*, high and low expression was consistently associated with low and high promoter DNAm, respectively, as evaluated over a total of 79 samples (Additional file 1: SI Fig. S3).

**Fig. 3:**
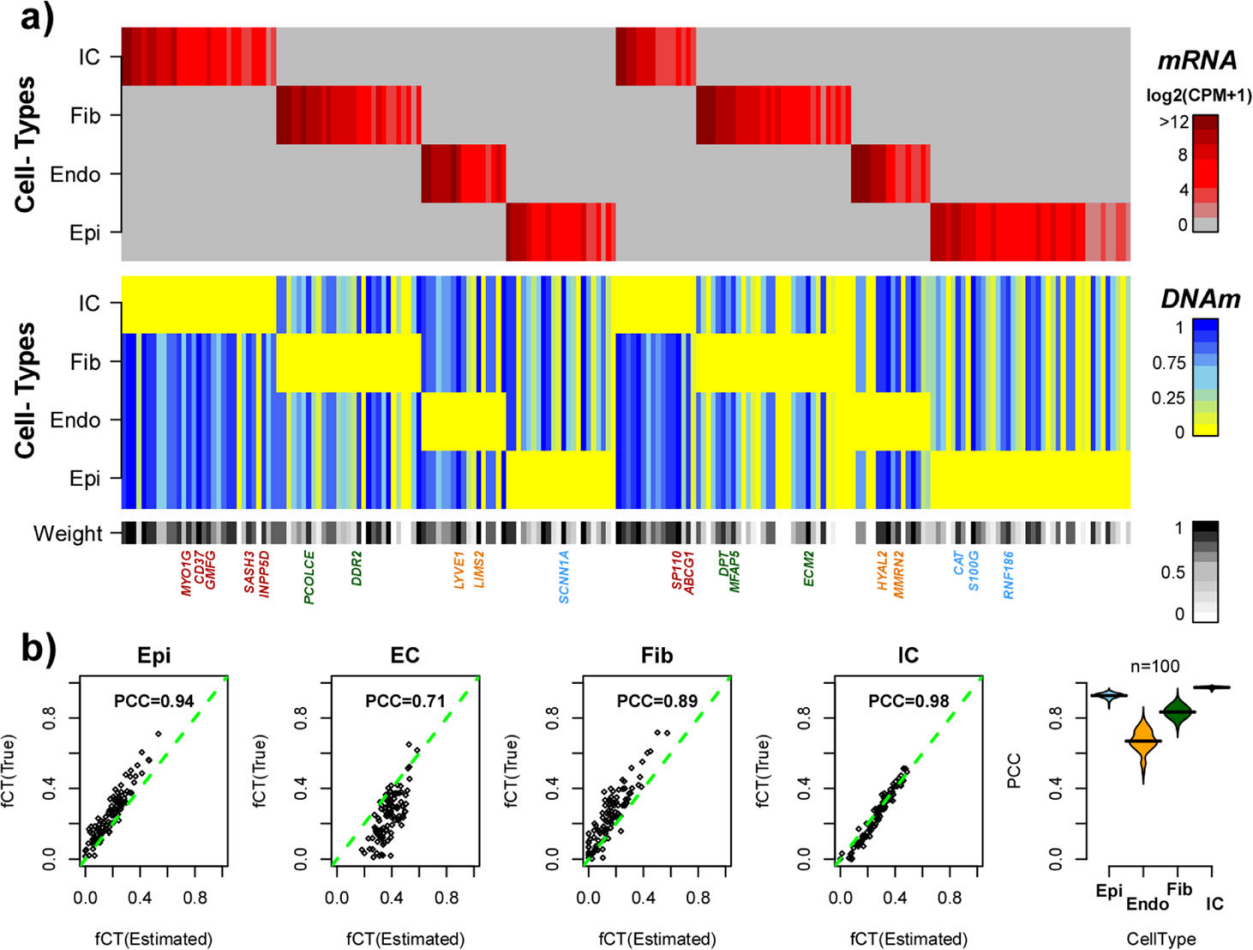
Validation of EPISCORE-derived DNAm reference matrix for lung. **a** Heatmaps representing the scRNA-Seq reference matrix for lung (top) and the corresponding inferred DNAm reference matrix (bottom), defined over the 4 main cell types found in lung tissue: Epithelial (Epi), endothelial (Endo), fibroblasts (Fib), and immune cells (IC) and over the common set of marker genes. Note that in the case of the DNAm reference matrix, each column represents the average DNAm within 200 bp of the transcription start site of the gene. The colorbar at the bottom displays the weight of the corresponding gene with weights close to 1 indicating genes for which differential DNAm is highly associated with differential expression across the 4 cell types. Examples of genes with weights > 0.8 and which are known markers for their respective cell types are shown. **b** Validation of the inferred DNAm reference matrix in **a** using 100 in silico generated mixtures of DNAm 450k profiles representing purified epithelial, endothelial, fibroblast, and immune cells derived from independent studies. The first four panels display scatterplots of the estimated cell type fraction (*x*-axis) vs. the true fraction (*y*-axis) for each main cell type, with the Pearson correlation coefficient (PCC), as shown. The last panel displays density plots of the PCC for each cell type (as estimated over 100 in silico mixtures), as obtained from 100 different runs

Based on this core set of anti-correlated genes, we next developed a probabilistic model of epigenetic regulation that would allow us to infer likely promoter DNAm from gene expression (the “Methods” section). This model was learned separately for each of these core genes in each of the sequencing and array-based sets, and subsequently applied to any genes overlapping with the lung-specific scRNA-Seq reference matrix, in order to infer corresponding DNAm values (the “Methods” section, Additional file 1: SI Fig. S4). We verified that the DNAm imputation procedure was robust to the choice of parameter significance thresholds (the “Methods” section, Additional file 1: SI Fig. S5). This resulted in two separate DNAm reference matrices (one from each of SCM2 and RMAP), consisting of 131 and 197 marker genes, respectively. The number of marker genes in the lung expression reference overlapping with imputable core genes from the sequencing RMAP and array SCM2 sets was statistically significant (Binomial test *P* = 5e−5 for array-set, *P* = 1e−5 for sequencing-set), thus confirming our original hypothesis that marker genes (i.e., genes exhibiting highly cell type-specific expression) are enriched for genes for which promoter DNAm is predictable from gene expression. As part of the inference, marker genes were also assigned a quality weight score, reflecting the confidence of the imputation (the “Methods” section), resulting in reduced 88 and 170 core marker gene DNAm reference matrices, as derived from the array- and sequencing-based matched sets, respectively (Additional file 3). The two DNAm references matrices exhibited an overlap of 56 genes and were highly congruent with each other, displaying an average Pearson correlation of 0.92 across the 4 cell types (Additional file 1: SI Fig. S6), allowing generation of a merged consensus DNAm reference for lung, defined over the union of 202 unique marker genes and the same 4 cell types (Fig. 3a, Additional file 3). The significance of the 202 genes is well supported, as many are either well-known cell type-specific markers or exhibit cell- or tissue type-specific expression as determined by independent studies [35, 36]. For instance, *MYO1G* and *CD37* are well-known markers for immune cells (T and B lymphocytes), *ECM2* and *DDR2* for extracellular matrix/fibroblasts, *HYAL2* and *LYVE1* (lymphatic vessel endothelial hyaluronan receptor 1) for endothelial cells, and *SCNNA1* (sodium channel epithelial 1 subunit alpha) and *RNF186* for epithelial cells (Fig. 3a). Of note, the merged DNAm reference also incorporates quality scores for each marker gene (Fig. 3a, the “Methods” section), which we use as weights when inferring cell type fractions in a lung tissue sample (the “Methods” section).

To validate the lung-specific DNAm reference, we used it to estimate corresponding cell type fractions on simulated bulk samples, generated by in silico mixtures of purified epithelial, endothelial, fibroblast, and immune cell DNAm profiles drawn from independent studies (the “Methods” section). Although most of these purified samples were not specific to lung tissue, we reasoned that our DNAm reference should nevertheless still be able to correctly identify generic epithelial, endothelial, fibroblast, and immune cells within these mixtures. Confirming this, estimated cell type fractions for the mixtures were strongly correlated with the true fractions, displaying an average Pearson correlation coefficient over the 4 cell types of 0.85 (Fig. 3b). We verified that this validation, as well as the high classification accuracy of the scRNA-Seq reference matrix, is robust to the choice of algorithm parameters (Additional file 1: SI Fig. S7a-b). We also investigated performance for an imputed DNAm reference derived at a higher cellular resolution of 6 cell types (epithelial, endothelial, fibroblast, lymphocyte, monocyte, and myeloid cells), revealing lower but still highly significant correlations (Additional file 1: SI Fig. S8). All these results demonstrate that it is possible to successfully translate a tissue-specific reference matrix at the mRNA expression level into a corresponding one at the DNAm level.

### EPISCORE reveals DNAm alterations in lung cancer endothelial cells

To validate EPISCORE on real mixtures and to demonstrate how it can lead to novel biological insight, we applied our inferred lung tissue DNAm reference matrix to the lung squamous cell carcinoma (LSCC) DNAm (Illumina 450k) dataset from TCGA [37]. We first estimated sample-specific cell type fractions for all major cell types (total epithelial, endothelial, fibroblast, and immune cell). Consistent with observations from scRNA-Seq studies [12] and with independent estimates of epithelial and immune cell fractions obtained using our HEpiDISH algorithm [19], the epithelial and immune cell fractions were dominant, with endothelial cells making up a smaller yet also substantial component of lung tissue (Additional file 1: SI Fig. S9a). The epithelial fraction was significantly increased in cancer compared to normal lung, consistent with the growth of cancer epithelial cells, while the endothelial proportion decreased (Additional file 1: SI Fig. S9b). We observed reasonably good correlation between sample specific epithelial and immune cell fractions obtained using the EPISCORE reference compared to those obtained using our previous HEpiDISH reference and algorithm (Additional file 1: SI Fig. S9c). However, for the non-immune stromal component (i.e., combined endothelial and fibroblasts), the correlation was much weaker (Additional file 1: SI Fig. S9c), which is not unexpected since the HEpiDISH DNAm reference does not include an endothelial component [19].

**Fig. 4:**
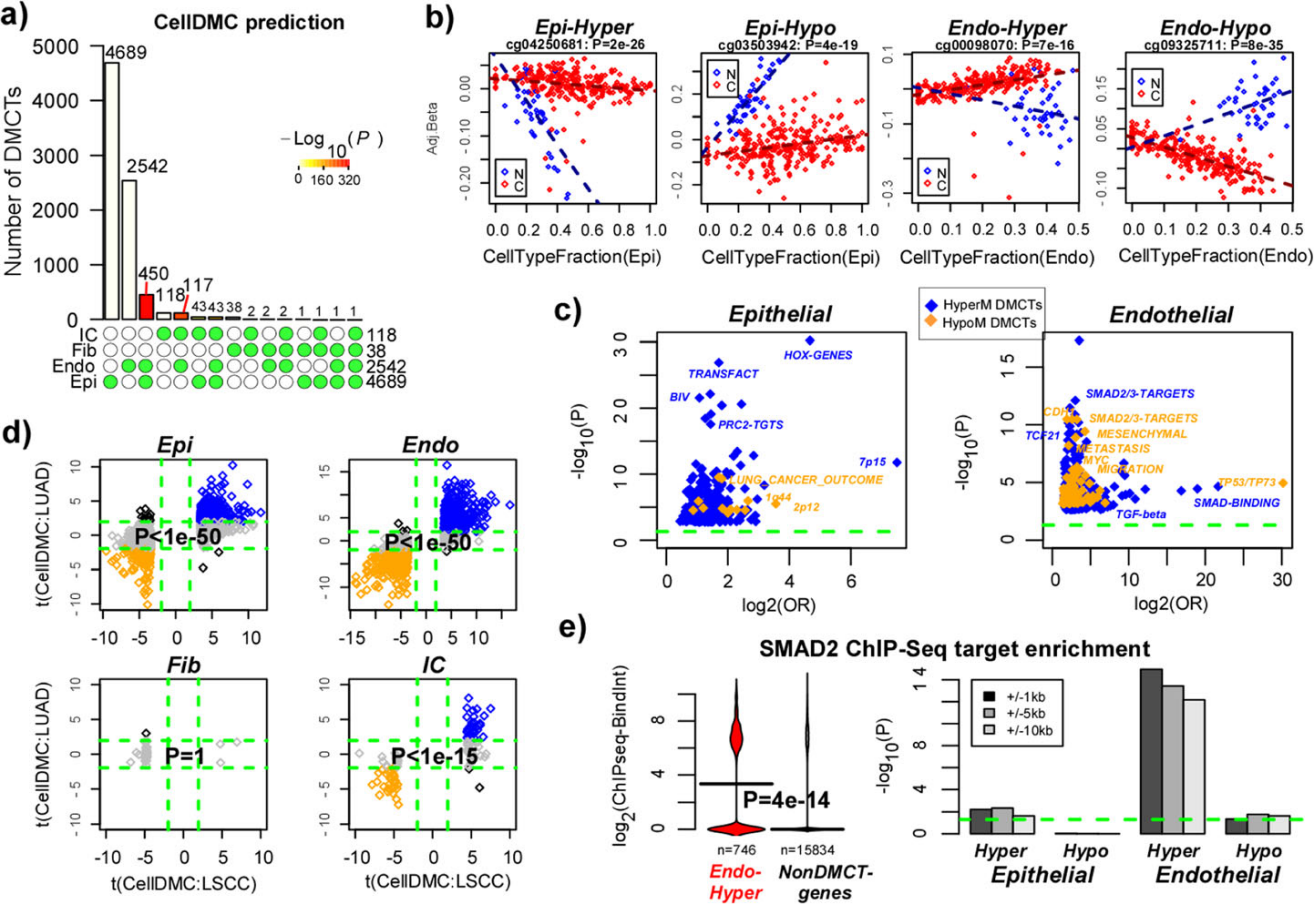
EPISCORE reveals epigenetic remodeling of endothelial cells in lung cancer. **a** Landscape barplot of CellDMC’s predictions for the number of lung-cancer associated DMCTs (*y*-axis) in each of the 4 major cell types, and their overlaps, as indicated beneath the barplots. For instance, there were 450 DMCTs occurring in both endothelial and epithelial cells and only 1 CpG commonly differentially methylated across all 4 cell types. Red barplots indicate highly significant overlaps of DCMTs occurring in respective cell types. **b** Four examples of DMCTs, two in the epithelial and two in the endothelial compartment. Each scatterplot displays the DNAm beta value adjusted for cell type fractions (*y*-axis) vs. the estimated fraction of the relevant cell type (*x*-axis). Specific CpG and associated *t* statistic *P* value of the DMCT are given. N, normal samples (blue); C, cancer samples (red). **c** Scatterplot of statistical significance (− log_10_*P*, *y*-axis) vs. logged odds ratio (log_2_OR, *x*-axis) for biological terms enriched among the epithelial (left) and endothelial (right) DMCTs respectively. Green dashed line indicates the line FDR < 0.05. Blue (orange) denote biological terms enriched among cancer hypermethylated (hypomethylated) DMCTs. **d** For each of the main cell types (epithelial, endothelial, fibroblast, and immune cell), scatterplot of the *t* statistics from CellDMC comparing LSCC to normal-adjacent lung tissue (*x*-axis) vs. the corresponding *t* statistics comparing LUAD to normal-adjacent lung tissue (*y*-axis). CpGs that show consistent significant cell type-specific hypermethylation (hypomethylation) between the two TCGA lung cancer studies are displayed in blue (orange). *P* value is from a one-tailed Fisher test assessing the statistical significance of the correlation. Green dashed lines correspond to the FDR < 0.05 line, as determined in the LSCC study. **e** Bean density plots of SMAD2 ChIP-Seq binding intensity values in HUVEC endothelial cell line for hypermethylated endothelial DMCT genes in LSCC (red) vs. genes not associated with any DMCTs. Binding peaks falling within a ± 5 kb window of a gene’s TSS was used to assign peaks to genes. *P* value is from a one-tailed Wilcoxon rank sum test. Horizontal lines indicate the median values in each group. Barplots display the corresponding − log_10_[*P* values] of a one-tailed Wilcoxon rank sum test for genes belonging to each of the main DMCT categories. Results are shown for SMAD2 binding targets defined at 3 different window sizes (± 1 kb, ± 5 kb, ± 10 kb). Green dashed line denotes the line *P* = 0.05

Next, we used the estimated cell type fractions as input to our CellDMC algorithm [24] in order to identify cell type-specific differentially methylated cytosines (DMCTs) between normal and cancer tissue. At an FDR < 0.05 threshold, we observed a total of 4689 epithelial DMCTs, 2542 endothelial DMCTs, 38 fibroblast DMCTs, and 118 immune cell DMCTs (Fig. 4a). The great majority of these DMCTs were cell type-specific (Fig. 4a). For instance, of the 38 fibroblast DMCTs, only 2 were also altered in endothelial and immune cell compartments, and only 1 was altered in epithelial cells (Fig. 4a). However, some cell types also exhibited statistically significant overlaps: notably, there were 450 cytosines that were altered in both epithelial and endothelial cells (*P* < 1e−300) and 117 cytosines that were altered in both endothelial and immune cells (*P* < 1e−300) (Fig. 4a). Plots of DNAm beta values adjusted for cell type fractions against the fraction of the altered cell type for top-ranked DMCTs confirmed the presence of highly significant interactions between normal/cancer status and cell type fraction (Fig. 4b).

To gain insight into the nature of these DMCTs, we performed GSEA [38, 39] (the “Methods” section). While the number of fibroblast and immune cell DMCTs was too small to attain any enrichment, for epithelial and endothelial DMCTs, we observed significant enrichment for many biological terms (Fig. 4c), which in general were highly specific to the actual cell type. For instance, among cytosines undergoing hypermethylation in tumor epithelial cells, we observed strong enrichment for polycomb-repressive-complex-2 (PRC2) targets and bivalently marked genes, transcription factors (TFs), and specially also *HOX*-genes (Fig. 4c). The observed hypermethylation at PRC2-marked and bivalent genes, many of which encode TFs, represents a well-known universal cancer DNAm signature which has been clearly shown to reflect DNAm changes in the epithelial compartment of solid tumors [40–48]. Thus, that CellDMC retrieves this known epithelial cancer signature in the epithelial compartment further validates our EPISCORE DNAm reference. In the epithelial compartment, we also observed enrichment for genes localized to specific cytobands (e.g., Chr7p15, Chr2p12, Chr1q44) (Fig. 4c), which in the case of Chr7p15 reflects the preponderance of *HOX*-genes at this locus. Other loci may reflect DNAm changes exhibiting increased variance due to underlying copy number variation (CNV) in the tumor epithelial cells. For instance, *CTNNA2* in Chr2p12 is frequently hypermethylated but also frequently gained in LSCC [37]. Also specific to the epithelial compartment, we observed enrichment for a signature conferring poor outcome in lung cancer [49] (Fig. 4c), which included general cancer prognostic genes like *AURKB*, *EXO1*, *MAD2L1*, *CDCA8* [50], and kinesins like *KIF15* [51]. In the endothelial compartment, we observed strong enrichment for terms related to cell migration and metastasis, TGF-beta signaling, and for binding targets of TCF21 (a well-known mesoderm factor) and SMAD2/3 (Fig. 4c). The combined enrichment of terms related to tumor invasion, TGF-beta signaling, and SMAD binding targets in the endothelial compartment is especially noteworthy, given that previous studies have implicated TGF-beta/SMAD signaling in the transformation of endothelial cells into a mesenchymal phenotype (EndoMT) and in lung cancer invasion [52–54]. Supporting the view that an EndoMT process may be involved, we also observed concomitant enrichment for genes upregulated in mesenchymal cells (Fig. 4c). Importantly, the enrichment for SMAD-binding targets and mesenchymal genes was not as strong, or not observed, in the epithelial compartment, consistent with the role of TGF-beta and SMAD signaling in the endothelial vasculature and thus with an epigenetic EndoMT.

To validate these findings, we applied EPISCORE and CellDMC to the lung adenocarcinoma (LUAD) TCGA cohort [55]. Epithelial, endothelial, and immune cell-specific DMCTs associated with LSCC validated with high statistical significance in LUAD, demonstrating that the DMCTs are reproducible, but also indicating that the associated DNAm changes are largely independent of lung cancer type (Fig. 4d). In line with this, GSEA results were in general also validated: in LUAD, we also observed epithelial-specific hypermethylation at transcription factors and PRC2 targets, epithelial-specific hypomethylation associated with a poor outcome signature in lung cancer, and endothelial-specific differential methylation at SMAD2/3 targets (Additional file 1: SI Fig. S10). To further ascertain that differential DNAm in lung cancer is enriched among SMAD2/3 targets and that this is happening preferentially in the endothelial compartment, we collated genome-wide ChIP-Seq binding intensity profiles for SMAD2/3 in endothelial cells and other cell types from the ChIP-Seq atlas resource [56]. For SMAD2, ChIP-Seq binding profiles in an endothelial cell line (HUVEC) was available, which confirmed that SMAD2 targets were enriched specifically among endothelial DMCTs, and specially among hypermethylated sites (Wilcox test *P* < 1e−14, Fig. 4e). These results were also observed for SMAD3 and were robust to the choice of window-size for associating ChIP-Seq binding peaks to genes (Fig. 4e, Additional file 1: SI Fig. S11).

### Validation of EPISCORE in breast tissue

**Fig. 5:**
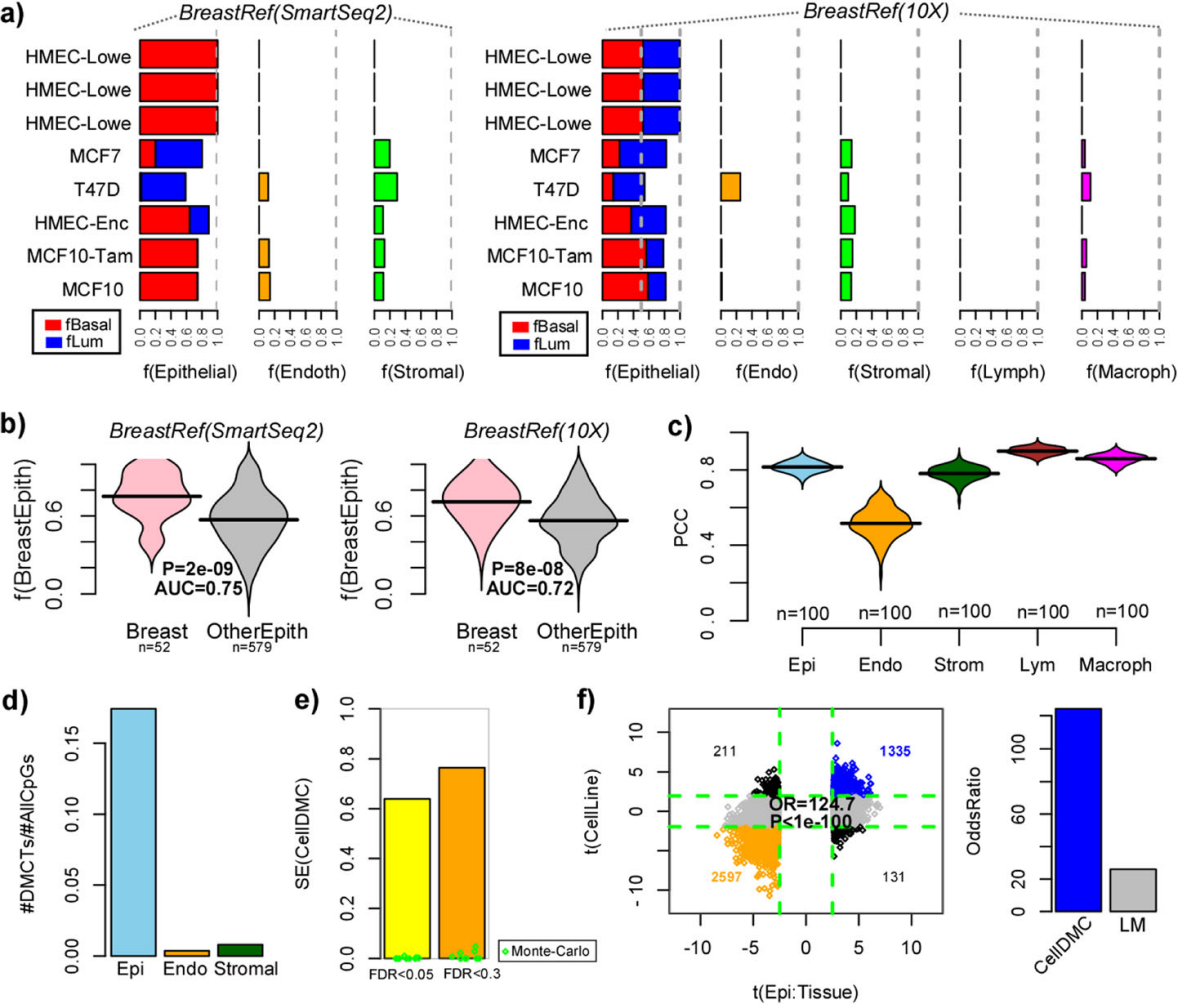
Validation of EPISCORE in breast cancer. **a** Barplots displaying for a number of mammary epithelial cell lines, their estimated epithelial (basal + luminal), endothelial, and stromal fractions as inferred using the imputed DNAm reference derived from the breast SmartSeq2 single-cell atlas (left panel). Right panel as left, but for the imputed DNAm reference derived from the breast tissue 10X single-cell atlas, which also contained references for lymphocytes and macrophages. **b** Beanplots displaying the estimated epithelial fraction (using one of the two imputed breast DNAm reference matrices, as indicated) for breast cancer cell lines and cancer cell lines derived from other epithelial tissues. *P* values derive from a one-tailed Wilcoxon rank sum test. **c** Beanplots displaying the Pearson correlation coefficient (PCC) between the estimated and true cell type fractions for each of the cell types in the 10X-derived DNAm reference matrix. There is one PCC value for each of 100 different Monte-Carlo runs, and for each run, the PCC is obtained by comparing estimated to true cell type fractions for 100 in silico mixtures. **d** Barplots comparing the fraction of DMCTs (FDR < 0.05) between triple-negative and ER+ breast cancer tissue occurring in each cell type, as inferred using CellDMC and the imputed breast DNAm reference (SmartSeq2). The number of DMCTs is expressed as a fraction of the total number of CpGs assayed. **e** The sensitivity (SE) of CellDMC to capture a gold standard set of epithelial-specific DMCTs derived by comparing triple-negative and ER+ breast cancer cell lines, in the corresponding breast cancer tissue DNAm dataset. SE is displayed at two different FDR thresholds, as indicated. Green datapoints represent the results for typical Monte-Carlo runs where the breast cancer subtype labels were randomized. **f** Scatterplot of *t* statistics between triple-negative and ER+ breast cancer tissue for significant (FDR < 0.05) epithelial DMCTs, as derived with CellDMC and the imputed DNAm reference (*x*-axis) vs. the corresponding *t* statistic as derived by comparing triple-negative to ER+ breast cancer cell lines (*y*-axis). The odds ratio (OR) and *P* value derived from a one-tailed Fisher test, quantifying the agreement between the DNAm changes in the epithelial cells of the tissue and epithelial cell lines is given. Barplot to the right compares the odds ratio for CellDMC to correctly identify epithelial DMCTs to that of an ordinary linear model which calls differentially methylated cytosines without regard to cell type

To demonstrate the generality of EPISCORE, we next considered the case of breast, a complex tissue consisting mainly of adipocytes, epithelial (basal and luminal), fibroblasts, endothelial, and immune cells. Although the MCA1 breast tissue atlases failed to capture adipocytes, we nevertheless proceeded to construct scRNA-Seq reference matrices from each of the SmartSeq2 and 10X platforms (the “Methods” section). The resulting scRNA-Seq reference matrices validated extremely well in the corresponding scRNA-Seq data from the other platform, achieving in general over 95% classification accuracy (the “Methods” section, Additional file 1: SI Fig. S12). From each of the SmartSeq2 and 10X-derived scRNA-Seq references, we next inferred corresponding breast tissue DNAm reference matrices (Additional file 4). To validate these, we first assessed them in a number of breast and breast cancer epithelial cell lines profiled with Illumina 450k DNAm beadarrays by ENCODE. As required, the imputed DNAm reference matrices correctly predicted these cell lines to be of epithelial origin (Fig. 5a). Moreover, cell lines previously categorized as having mainly luminal (MCF7, T47D) and basal characteristics (HMEC, MCF10) were generally predicted to be such (Fig. 5a). Similar results were obtained when applying the DNAm reference matrices to a total of 30 breast cancer cell lines for which their luminal/basal classification has been previously established [57, 58] (Additional file 1: SI Figs. S13-S14). To demonstrate the specificity of the breast tissue DNAm epithelial reference, we compared the estimated epithelial fraction for the breast cancer cell lines to those originating from other epithelial tissues, which revealed significantly higher fractions in the breast cell lines (Fig. 5b, Wilcox test *P* < 1e−7). In silico mixtures of samples representing purified cell types further confirmed the overall correlative accuracy of the imputed DNAm references (Fig. 5c). As with the lung, we verified that the classification and correlative accuracy of the scRNA-Seq and DNAm references is robust to algorithm parameter choices (Additional file 1: SI Fig. S7c-d).

As further validation, we applied CellDMC with the EPISCORE-derived breast tissue DNAm reference to identify DMCTs between 30 triple negative (TN) and 254 estrogen receptor-positive (ER+) breast cancers [47], which generally originate from distinct basal and luminal layers, respectively, and which should therefore exhibit DNAm differences mostly within the corresponding epithelial compartment. To assess sensitivity, we separately defined a gold standard list of 11,488 CpGs that are differentially methylated (FDR < 0.05) between TN and ER+ breast cancer cell lines and therefore known to be altered in the epithelial compartment (Additional file 5) [57, 58]. Validating our EPISCORE DNAm reference, CellDMC identified 90,419 DMCTs (FDR < 0.05) between TN and ER+ breast cancers (i.e., 19% of all measured > 485,000 CpGs), of which the overwhelming majority (84,682, i.e., 94%) occurred in epithelial cells (Fig. 5d). CellDMC achieved over 60% sensitivity to detect the gold standard list, compared to effectively zero sensitivity when the breast cancer labels were randomized, clearly demonstrating that the identified epithelial DMCTs are *bona fide* TN-ER+ DMCTs (Fig. 5e). Importantly, epithelial DMCTs derived by comparing ER+ to TN breast cancer tissue exhibited very strong consistency with corresponding DNAm changes obtained by comparing ER+ to TN breast cancer cell lines, demonstrating substantially improved sensitivity and specificity over an ordinary linear model which does not call DMCTs (Fig. 5f).

### Validation of EPISCORE in peripheral blood mononuclear cells

**Fig. 6:**
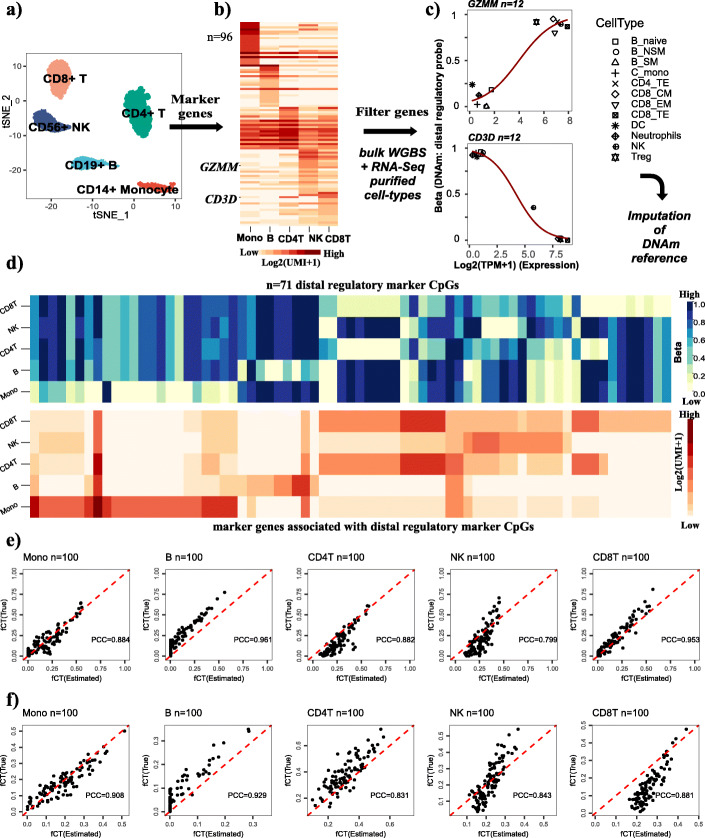
Proof of EPISCORE concept in peripheral blood. **a** Dimensional reduction and t-SNE embedding of a 10X dataset profiling 68,000 peripheral blood mononuclear cells (PBMCs), depicting the resulting cell clusters, annotated to PBMC cell types using a bulk RNA-Seq reference matrix for PBMCs. **b** The expression reference matrix over 5 main PBMC cell subtypes and 96 marker genes, as constructed from the scRNA-Seq data in **a**. **c** Two examples of marker genes, where differential expression across blood cell subtypes (as assessed using independent bulk RNA-Seq and DNAm data of purified blood cell types) is associated with corresponding differential DNAm at a CpG mapping to a distal regulatory element of the gene. The *y*-axis labels DNAm at this CpG, *x*-axis labels the normalized gene expression value, and datapoints are shown for all matching blood cell subtypes. A fit from a logistic regression is shown, which is used to impute DNAm at this CpG from the expression values in the scRNA-Seq reference matrix, after suitably normalizing the expression data. **d** Heatmaps displaying the imputed DNAm reference matrix for 71 marker CpGs (top panel) and the corresponding scRNA-Seq reference matrix for X marker genes associated with these 71 CpGs. **e** Validation of the imputed DNAm reference matrix using in silico mixtures of PBMC cell types, using Illumina 450k profiles from Reinius et al. For each of the 5 PBMC cell types, we plot the estimated fractions (*x*-axis) vs. the true fractions (*y*-axis), for each of 100 in silico mixtures. Mixture weights were simulated from a uniform Dirichlet distribution. PCC values are given. **f** As **e**, but with mixture weights drawn from a non-uniform Dirichlet distribution, with mean and variance for each cell type matched to observed data

Although constructing detailed DNAm reference matrices for hematological tissues is feasible using purified cell type samples generated via FACS/MACS sorting [22, 23], we nevertheless decided to also evaluate EPISCORE in such tissues, in order to demonstrate that imputation of a DNAm reference matrix at a higher cellular resolution (i.e., for more similar cell types) is possible by starting out from a scRNA-Seq atlas. Because neutrophils have low RNA content and are not readily captured in scRNA-Seq assays, we focused on a 68,000 peripheral blood mononuclear cell (PBMCs) 10X scRNA-Seq atlas [59]. From this atlas, we built a scRNA-Seq reference matrix for 5 cell types (monocytes, natural-killer (NK) cells, B cells, CD4+ and CD8+ T cells) (Fig. 6a, b), which we subsequently validated in independent bulk samples representing purified blood cell subtypes (Additional file 1: SI Fig. S15-S16). Due to the need to discriminate more similar cell types from each other (e.g., B cells, NK cells, CD4+ and CD8+ T cells), we extended the DNAm imputation framework to include enhancer elements (the “Methods” section, Fig. 6c, Additional file 1: SI Fig. S17). This resulted in an imputed DNAm reference matrix for PBMCs defined over 71 marker CpGs (Fig. 6d). Finally, we validated the DNAm PBMC reference matrix on in silico mixtures generated from independent DNAm profiles representing purified blood cell subtypes (Fig. 6e, f). Of note, although the PBMC DNAm reference matrix does not contain granulocytes, it validated reasonably accurately in whole blood and experimentally reconstructed whole blood samples with known cell type proportions (Additional file 1: SI Fig. S18). Thus, imputation of a DNAm matrix encompassing very similar cell types is possible from a corresponding scRNA-Seq atlas.

## Discussion

Here, we have shown that in silico microdissection of DNAm profiles generated from bulk tissue samples can be accomplished using a relevant tissue-specific DNAm reference matrix inferred from a corresponding high-resolution tissue-specific scRNA-Seq atlas. Underlying the success of this approach is the demonstration that for a proportion of cell type-specific marker genes, their promoter DNAm levels can be predicted from their measured expression levels. Importantly, while this imputation step is only approximate, and not even possible for many marker genes, we have shown that it is nevertheless accurate enough for reasonable numbers of marker genes to allow meaningful inferences to be made. Supporting this, we previously demonstrated that reference-based inference of cell type fractions is very robust to potential inaccuracies in the reference matrix, i.e., reference-based inference can tolerate even up to 30% of wrong marker genes, as long as the total pool of correct marker genes is big enough [19]. Supporting the robustness of EPISCORE, we have demonstrated proof of concept across three different tissue types (lung, breast, and peripheral blood mononuclear cells), encompassing many different datasets. Thus, EPISCORE can be applied to the many thousands of bulk tissue genome-wide DNAm profiles that are freely available in public data repositories and for which cell type deconvolution would otherwise not be possible. We note that this applies mainly to complex solid tissues, for which identifying, purifying, and generating DNAm profiles for all major cell types within the tissue is currently infeasible.

Demonstrating how EPISCORE can lead to an improved interpretation and understanding of epigenome data, we applied EPISCORE in conjunction with CellDMC to two large lung cancer DNAm sets from TCGA, revealing novel epigenetic alterations in endothelial cells that were characteristic of an endothelial to mesenchymal transition (EndoMT). Indeed, within lung endothelial cells, we observed strong enrichment of cancer associated DNAm changes at binding targets of SMAD2/SMAD3, transcription factors that are known to be indispensable for vascular stability and integrity [60] and which mediate TGF-beta induced EndoMT in cancer [52, 54]. Of note, the enrichment of endothelial DMCT genes for SMAD2/3 binding targets was especially pronounced among hypermethylated sites, consistent with an interpretation in which SMAD binding and signaling is disrupted in lung cancer endothelial cells, leading to EndoMT and destruction of vascular endothelial barriers. The ensuing disruption of such cell-cell junctions would increase the permeability of lung capillaries, facilitating trans-endothelial passage of tumor cells [53], and may explain our observation of a concomitant enrichment for terms relating to cell-migration and metastasis. Supporting an EndoMT interpretation, we also observed enrichment for genes imparting a mesenchymal phenotype, which could reflect the increase in cancer-associated fibroblasts that accompanies EndoMT [54]. We note that although an EndoMT process underpinning cancer invasion and metastasis has been suggested previously [52, 53, 61], our novel analysis reveals DNAm changes in endothelial cells that could underpin this transformation, if not causally, at least as a useful associative EndoMT marker.

It is important to also discuss some of the limitations and opportunities related to EpiSCORE.

One potential limitation is the tissue-specific scRNA-Seq atlas itself, as current scRNA-Seq assays are still biased towards cell types that are viable. For instance, adipocytes are abundant in breast tissue, yet are not readily captured in scRNA-Seq assays due to their larger size [12]. Despite this, EPISCORE was still able to yield valuable inferences for specific phenotypes such as breast cancer, probably because the adipocyte fraction does not vary significantly between normal and cancer tissue [47] and because references for other stromal components (endothelial and fibroblast) may serve as reasonably good proxies for adipocytes. Another potential limitation and important question is the cellular resolution at which to run EpiSCORE. Here, we have shown that EPISCORE can work reasonably well for up to around 5 or 6 main cell types. Ideally, however, we would like EPISCORE to operate at the highest possible cellular resolution (i.e., typically involving 10 or more cell types). To achieve this will require improvements in scRNA-Seq technologies to capture larger numbers of cells of each type. In addition, our results indicate that it may be necessary to consider distal regulatory regions (e.g., enhancers). While we did not observe that inclusion of distal regulatory probes improved performance in lung and breast tissue, it was necessary when applying EpiSCORE to PBMCs, possibly due to the need to discriminate more similar cell types (e.g., lymphoid subsets) from each other. Thus, including distal regulatory sites into the EpiSCORE framework may be important if one desires inference at a higher cellular resolution in solid tissues, for instance, if one wishes to estimate distinct leukocyte proportions in a tissue like breast or lung. With ongoing improvements in mapping enhancer-gene networks [62–64], such an approach is likely to be fruitful. Single-cell ATAC-Seq data, generated separately or simultaneously with scRNA-Seq in the same cells [65, 66], may provide a means to hone in on cell type-specific enhancers, which could further improve inference. Alternatively, it may also be possible to adapt the HEpiDISH hierarchical inference framework [19], so as to integrate an EpiSCORE DNAm reference at a coarser resolution (e.g., 4–5 cell types) with a high-resolution (e.g., 7 cell types) immune cell-specific DNAm reference [18, 22, 23, 30], to infer cell type fractions for over 10 cell types. Finally, we note that another limitation on cellular resolution arises if there is a requirement to identify cell type-specific differential methylation signal associated with some outcome or exposure of interest. To achieve the latter, it is necessary to apply an algorithm such as CellDMC, using as input the cell type fractions estimated with EpiSCORE, yet reliable inference of cell type-specific differential DNAm may require thousands of samples if high cellular resolution (> 6–7 cell types) is desired. In summary, although we have only validated EPISCORE at a coarser resolution level of 4–6 cell types, we stress that in the context of solid tissues this is already a major advance, as there are currently no other cell type deconvolution methods and DNAm reference matrices that can be systematically applied to arbitrary bulk tissue DNAm profiles [19].

In conclusion, EPISCORE enables virtual microdissection of an arbitrary bulk DNAm profile and can help identify cell type-specific differential DNAm signal, thus paving the way for an improved interpretation and understanding of disease epigenome data.

## Methods

### Brief description of all datasets analyzed

*scRNA-Seq datasets used for construction and validation:*

- Lung tissue MCA1 [12]: For the SmartSeq2 and 10X scRNA-Seq datasets, we obtained the read counts matrices as provided by the respective Seurat R-objects, available from https://tabula-muris.ds.czbiohub.org, which were subsequently normalized by first scaling each cell by the ratio of the maximum total read count across all cells to the cell’s total read count, then adding a pseudocount of + 1, and finally taking a log_2_ transformation. Human homologs were obtained using the annotationTools BioC package. In the case of the SmartSeq2 dataset, there were a total of 1716 cells, consisting of 138 lung epithelial, 693 lung endothelial, 423 stromal, and 387 immune cells, with 75 cells falling into other categories. In the case of the 10X data, there were 49 lung epithelial, 462 lung endothelial, 2540 stromal, and 1743 immune cells.
- Breast tissue MCA1 [12]: Same description applies as to the lung tissue MCA1 sets. In the case of the SmartSeq2 dataset, there were 2405 cells, consisting of 1340 basal, 578 luminal, 47 endothelial, and 440 stromal cells. In the case of 10X, there were 4481 cells, consisting of 392 basal, 459 luminal, 251 endothelial, 700 stromal, 2493 lymphocytes, and 186 macrophages.
- Lambrecht et al. [28]: This is a 10X scRNA-seq dataset encompassing 52,698 cells from lung tissue (both normal and lung cancer). We downloaded the .Rds files from https://www.ebi.ac.uk/arrayexpress under accession number E-MTAB-6149. Of the 52,698 cells, 1709 were annotated as alveolar epithelial, 5603 as B cells, 1592 as endothelial, 1465 as fibroblasts (stromal), 9756 as myeloid cells, 24,911 as T cells, and 7450 as tumor epithelial cells, with 212 of unclear origin. Data within the .Rds objects was already normalized as log_2_(CPM+1).

*DNA methylation datasets used for validation:*

- Reinius et al. [67]: This is an Illumina 450k DNAm dataset of purified blood cell subtypes, which included 6 CD14+ monocyte, 6 CD19+ B cell, 6 CD4+ T cell, 6 CD56+ NK cell, and 6 CD8+ T cell samples. Data is available from GEO (http://www.ncbi.nlm.nih.gov/geo/under accession number GSE35069) and was processed as in our previous publication [30].
- LSCC TCGA [37]: Illumina 450k DNAm dataset profiling 275 lung squamous cell carcinomas (LSCC) and 41 normal-adjacent lung samples, available from https://portal.gdc.cancer.gov/. Data was processed and normalized as described previously [68].
- LUAD TCGA [55]: Illumina 450k DNAm dataset profiling 399 lung adenocarcinomas (LUAD) and 32 normal adjacent lung samples, available from https://portal.gdc.cancer.gov/. Data was processed and normalized as described previously [68].
- Erlangen breast tissue [47]: Illumina 450k DNAm dataset profiling 50 normal breast tissue samples, 305 breast cancers, and 42 normal-adjacent tissues. Among the 305 breast cancers, 30 were triple-negative (ER−, PR−, HER2−) and 254 estrogen receptor-positive (ER+). Data is freely available from GEO (http://www.ncbi.nlm.nih.gov/geo/under accession number GSE69914) and was processed as in our previous publication [47].
- Cancer cell lines [58]: Illumina 450k DNAm dataset profiling 1028 cancer cell lines, encompassing 13 tissue types. A total of 52 breast cancer cell lines were profiled. Raw idat files were processed with minfi. Illumina definition of beta value was used. Only probes with less than 5% missing values were used, resulting in 482,106 probes. Missing values were imputed using impute.knn (*k* = 5) function from impute BioC package. Data is freely available from GEO (http://www.ncbi.nlm.nih.gov/geo/ under accession number GSE68379).
- ENCODE [32]: Illumina 450k DNAm profiles for 63 cell lines. Raw data was processed with minfi. Illumina definition of beta value was used. The very small proportion of missing values were imputed using impute.knn (*k* = 5) function from impute BioC package, resulting in 485,512 probes for analysis. Data is freely available from GEO (http://www.ncbi.nlm.nih.gov/geo/ under accession number GSE40699).
- Pulmonary epithelial cells (PECs) [69]: Illumina 450k DNAm profiles for 18 pulmonary endothelial cell samples. Raw data was processed with minfi. Illumina definition of beta value was used. Only probes with no missing values were retained, resulting in 484,081 probes. Data is freely available from GEO (http://www.ncbi.nlm.nih.gov/geo/ under accession number GSE84395).
- Breast fibroblasts and endothelial cells [70]: Illumina 450k DNAm profiles for 2 mammary fibroblast and 2 mammary endothelial samples. Raw data was processed with minfi. Illumina definition of beta value was used. Only probes with no missing values were retained, resulting in 477,809 probes. Data is freely available from GEO (http://www.ncbi.nlm.nih.gov/geo/ under accession number GSE74877).
- HMECs [71]: Illumina 450k DNAm profiles for human mammary epithelial cells (in triplicate). Data was processed as described by us previously [47]. Data is freely available from GEO (http://www.ncbi.nlm.nih.gov/geo/ under accession number GSE56719).

*Matched DNA methylation and gene expression databases:*

- SCM2: One of the matched DNA methylation gene expression datasets derives from the Stem-Cell-Matrix Compendium-2 (SCM2) [34], which used Illumina beadarrays (450k and HT12v3), and is freely available from GEO under accession number GSE30654. Illumina gene expression and Illumina DNAm 450k data was processed as described previously [44, 72]. We built an integrated set defined over 13,290 common Entrez gene IDs and 34 samples. The 34 samples included primary epithelial, mesenchymal, and fibroblast cells (*n* = 20), as well as 14 somatic fetal and adult primary cells encompassing different cell types, including adipocytes, adrenal gland, bladder, kidney, liver, lung, lymph nodes, muscle, stomach, tongue, and ureter. In the case of DNAm, we assigned a unique DNAm value to a given gene using the same validated procedure as implemented in our FEM algorithm [73], which uses the average of CpGs within 200 bp upstream of the transcription start site (TSS), or if no such probes are available the average of 1st exon probes.
- Epigenomics Roadmap (RMAP): The other matched DNAm gene expression dataset derives from the Epigenomics Roadmap [31, 33] and is based on sequencing data. Specifically, the gene expression is bulk RNA-Seq data, while the DNAm profiles derive mainly from whole genome bisulfite sequencing (WGBS) and MeDIP-Seq, with one sample generated with reduced representation bisulfite sequencing (RRBS). Data was downloaded from https://egg2.wustl.edu/roadmap/web_portal/meta.html. The bulk RNA-Seq was obtained as RPKM, which was subsequently log-transformed using a pseudocount of + 1. In the case of DNAm, we only used CpGs covered by at least 10 reads. In order to assign a unique DNAm value to any given gene, we averaged the DNAm beta values for CpGs within 200 bp upstream of the TSS. We built an integrated set defined over 18,651 common Entrez gene IDs and 45 samples, which included hESCs, derived cells representing all major developmental layers (endoderm, ectoderm, mesoderm, mesoendoderm, mesenchymal, neural progenitor, trophoblast, CD34+ primary cells) and samples from different tissue types including keratinocytes, liver, brain, esophagus, intestine, stomach, lung, ovary, pancreas, muscle, colon, atrium, ventricle, small intestine, thymus, spleen, aorta, breast myoepithelial cells, CD4+ and CD8+ T cells, foreskin fibroblasts, and foreskin melanocytes.

### The EPISCORE algorithm

The aim of the algorithm is to generate a DNAm reference matrix for any solid tissue for which a corresponding tissue-specific scRNA-Seq atlas exists, with the ultimate goal to then use this tissue-specific DNAm reference matrix to (i) infer proportions of the main cell types in a bulk tissue DNAm profile and (ii) to identify which cell types are altered epigenetically in the context of a given disease epigenome study. The key idea underlying EPISCORE is to take advantage of the high-resolution screen of cell types within a solid tissue, as provided by a scRNA-Seq assay, to build representative DNAm profiles for these cell types through a probabilistic imputation procedure, which in effect translates the representative scRNA-Seq profiles of the cell types in the tissue into corresponding profiles at the DNAm level. Thus, broadly speaking, there are 4 main steps that make up the EPISCORE algorithm: (i) construction of the scRNA-Seq reference matrix, (ii) genome-wide identification of genes for which cell type-specific variation in DNAm at its regulatory elements is associated with corresponding variation in gene expression, (iii) development and application of a probabilistic imputation model in order to impute a corresponding tissue-specific DNAm reference matrix, and (iv) application of this DNAm reference to bulk DNAm profiles of the same tissue to infer cell type fractions and cell type-specific differential DNAm.

#### Step (i): Construction of a tissue-specific scRNA-Seq expression reference

Given a normalized and clustered scRNA-Seq dataset from a given tissue, where all main cell types in the tissue are represented, and with well-defined clusters annotated to known cell types, we construct an expression reference matrix using the following procedure. First, we decide on the resolution level desired. For instance, various types of immune cells that infiltrate the tissue may be considered as defining a generic immune cell. Similarly, different types of fibroblasts or endothelial cells could be merged to define a single generic fibroblast and endothelial subtype. Having established the main cell types of interest, we next perform differential expression (DE) between a given cell type and all others, i.e., if there are *K* cell types, we perform *K* separate DE analyses, ranking genes for each cell type according to the *P* value from a Wilcoxon rank sum test. *P* values are adjusted for multiple testing using Benjamini-Hochberg procedure. Next, for each cell type, we identify candidate marker genes as those satisfying the following conditions: (i) a false discovery rate (FDR) < 0.05, (ii) highest and non-zero median expression in the given cell type, (iii) and a marker specificity score (MSS) of *K-1*. The MSS of a given marker gene in a given cell type is defined as the number of other cell types (maximum value is therefore *K-1*) for which the median expression in each of these cell types is zero. Thus, our marker gene selection procedure supplements statistical significance with the additional requirements that the gene is most highly expressed in the given cell type and that it is not expressed in any other cell type (using medians to establish this). In case the number of marker genes for specific cell types is too low (say less than 100 or so), the marker specificity score requirement for that cell type can be relaxed. Ideally, one would also desire balanced numbers of marker genes for each cell type, yet it is difficult to know in advance how many of our marker genes will be selected for the DNAm imputation step. Thus, we typically recommend using all markers that satisfy the given conditions and to relax the MSS score in case there are too few markers for a given cell type. For instance, in the case of lung tissue, we constructed the scRNA-Seq and DNAm references at the resolution of 4 cell types (epithelial, endothelial, stromal, and immune cell) using the maximal possible MSS threshold (this value equals 3) for each of the 4 cell types. Here, the maximal value is 3 because there is maximally only 3 other cell types where a given marker gene is not expressed. In the case of breast tissue, we constructed the scRNA-Seq and DNAm references at the resolution of 6 cell types (basal, luminal, endothelial, stromal, lymphocytes, macrophages) and corresponding MSS thresholds for these cell types were 5, 5, 4, 4, 3, and 3, respectively. These choices were motivated so as to ensure sufficient and approximately equal numbers of markers for each cell type. Finally, for the resulting marker genes, we construct two scRNA-Seq reference matrices, one using the median over all cells within each cluster (cell type) and another using the average. In this work, we only use the median-based one however, because we infer cell type fractions within an unconstrained robust partial correlation framework for which taking the average is not a requirement. In the case of the mouse lung scRNA-Seq atlas (MCA1 SmartSeq2 assay) [12], we considered 4 main cell types (epithelial, fibroblast, endothelial and immune cell), which resulted in 716 epithelial, 131 endothelial, 274 fibroblast, and 172 immune cell-specific marker genes (after homology mapping from mouse to human). The final scRNA-Seq reference matrix for lung was defined over 1293 unique genes and 4 cell types. We recommend at least 100 marker genes or more for each cell type, as the imputation step to be carried out later will only be possible for 10–20% of these marker genes. Although marker genes are enriched for genes where the differential expression across cell types is associated with differential promoter DNA methylation, for the great majority of genes, there will be no significant association between DNAm and gene expression, resulting in approximately 10–20% of marker genes for which DNAm can be imputed.

#### Step (ii): Identification of “imputable” genes

Completely separate from the previous step, we develop a probabilistic imputation model that will allow us to eventually translate the scRNA-Seq reference matrix into a corresponding one for DNAm. Our key hypotheses are twofold: first, that for specific genes, variation in gene expression across different cell types is well explained by corresponding variation in DNAm as measured at regulatory elements associated with the gene, and second, that these genes would be enriched for marker genes (e.g., as those found in the previous step). In order to find these genes, we take a genome-wide approach analyzing two independent matched DNAm and mRNA expression datasets, correlating the two data types across the common samples. One dataset is sequencing based and is derived from the NIH Epigenomics Roadmap [33, 74], consisting of bulk RNA-Seq and WGBS/MeDIP-Seq DNAm data for a common set of 45 samples, representing a wide variety of different cell types. The other matched dataset is array-based, is derived from the Stem-Cell Matrix Compendium-2 (SMC2) [34], and consists of Illumina beadarray mRNA expression and 450k DNAm profiles for a common set of 34 samples, representing a wide variety of different primary cell and somatic tissue samples. In the case of DNAm, we considered the average promoter DNAm level (averaged over a 200 bp region upstream of the TSS, or if no probes/CpGs available in this region, we took the average over probes/CpGs in 1st Exon [73]). For each gene in each dataset, we thus determined if its expression was significantly associated with DNAm. Significant associations were determined using Pearson correlation coefficients (PCCs) and a nominal *P* value threshold level < 0.05. Although the relation between DNAm and gene expression can be non-linear, we find that a linear PCC works very well in capturing also non-linear patterns. This is because the non-linearities are still described by monotonic functions as opposed to non-monotonic (e.g., inverse quadratic) ones. Later when developing the imputation model in step (iii), we explicitly deal with potential non-linearities. We also note that before computing PCCs, genes were filtered based on two variability criteria: DNAm range > 0.1 and mRNA expression range > 1. Since not all samples have high purity, it is sensible to restrict to a DNAm range of 0.1, as DNAm changes of this order of magnitude have been shown to be highly reproducible and could be biologically significant. In the case of gene expression, the threshold of 1 was motivated by observing that it corresponds approximately to the difference in magnitude of the modes of expressed and non-expressed genes. Thus, with these variability criteria, we are more likely to capture biologically meaningful associations. Next, we focus on genes that exhibit anti-correlation. This is because the overwhelming majority (> 80%) of significant expression-promoter DNAm pairs exhibited such anti-correlation. Thus, for each of the matched datasets, we obtained sets of genes where expression variation is significantly associated with promoter DNAm. For the sequencing-based set, where we started out from 18,651 genes, we obtained 2174 anti-correlated genes, whereas for the SCM2 set we performed the analysis for 13,290 genes, finding 1152 anti-correlated genes. The two sets of anti-correlated genes exhibited a highly significant overlap of 516 genes (Fisher test *P* < 1e−300), which thus effectively proves our 1st hypothesis. To test the 2nd hypothesis involves evaluating the significance of the overlap of these gene sets with the marker genes making up a tissue-specific scRNA-Seq reference matrix, as obtained in step (i).

#### Step (iii): Development and application of probabilistic imputation model

The sets of anti-correlated genes found in previous step define features for which promoter DNAm is imputable from gene expression. Because the relation between promoter DNAm and gene expression can be non-linear, we adopted the following procedure to impute DNAm values. In general, for the anti-correlated genes, high expression is associated with low or near-zero promoter DNAm levels [33], so for a sample of high cellular purity and with high expression of the given gene, we assume a promoter DNAm value of zero. For a sample where the gene is not expressed, promoter DNAm is less informative (since the silencing could be caused by repressive histone marks), and hence for these cases, we developed the following imputation model: for each of the original full expression matrices from SCM2 and RMAP, we fitted a 2-state mixture of gamma distributions to all the expression values in the given matrix in order to learn the parameters for the unexpressed and expressed states. Parameters of the gamma distribution, as well as the mixing weights, were learned using an EM-algorithm [75], as implemented with *gammamixEM* from the *mixtools* R-package [76]. From the estimated parameters, we then use Bayes theorem to compute posterior probabilities that a gene is expressed given its measured expression value:

$$ p\left({E}_{gs}=1|{x}_{gs}\right)=p\left({x}_{gs}|{\hat{a}}^{(E)},{\hat{s}}^{(E)}\right){\hat{\pi}}^{(E)}/p\left({x}_{gs}\right) $$

where $p\left({x}_{gs}|{\hat{a}}^{(E)},{\hat{s}}^{(E)}\right)$ is a gamma distribution with ${\hat{a}}^{(E)},{\hat{s}}^{(E)}$ the estimated parameters of the gamma distribution, ${\hat{\pi}}^{(E)}$ is the estimated mixing weight for the expressed state, *x*_*gs*_ denotes the expression value of gene *g* in sample *s*, and where *E*_*gs*_ denotes a binary random variable with 1 indicating expressed state and 0 non-expressed state. Next, from each of the SCM2 and RMAP datasets, we identified the samples for which the given gene is not expressed using a posterior probability threshold of 0.2, i.e., samples where *p*(*E*_*gs*_ = 1| *x*_*gs*_) < 0.2 were declared to be ones where the gene is not expressed. Finally, the imputed value was obtained by taking the median promoter DNAm level over all the non-expressing samples. Of note, if for a given marker gene no sample passes the *p*(*E*_*gs*_ = 1| *x*_*gs*_) < 0.2 threshold, then it is not possible to impute DNAm values for the cell types in which this gene is not expressed, which thus renders this marker gene as non-imputable and must therefore be excluded. In order to salvage these marker genes, one can run logistic regressions with DNAm and expression as the dependent and independent variables, respectively. The estimated parameters of the logistic regression fit allow imputation of DNAm for the non-expressed state by setting the expression value to zero (i.e., in effect, by extrapolating the fit to the state of zero-expression). We note that imputed DNAm values are robust if we were to relax the *p(E|X) <* 0.2 threshold to say 0.3. This is because the data distribution on the probability scale is strongly bi-modal with genes either clearly non-expressed or expressed, by virtue of our 2-state mixture model. This means that few samples fall in the middle probability range (i.e., in between 0.2 and 0.5), and thus changing the threshold within this range cannot affect imputed estimates. On the other hand, imposing a more stringent threshold (e.g., 0.1) risks having more marker genes where DNAm must be imputed through a logistic regression model.

Using the above procedure, we can thus impute DNAm values from the scRNA-Seq reference matrix constructed in step (i), for all marker genes that overlap with the sets of anti-correlated genes derived in step (ii). Of note, marker genes highly expressed in the cell type they define will be assigned a promoter DNAm value of zero, whereas for marker genes not expressed in the given cell type, the imputed DNAm value derives from the Bayesian model or from a logistic regression fit. Ideally, for genes in the reference matrix that are not expressed in a particular cell type, the imputed DNAm value would be as close as possible to 1, making these genes particularly informative for the subsequent cell type deconvolution. Thus, in addition to the imputation, we also define a weight for each marker gene as the average of imputed DNAm values for the cell types where the marker gene is not expressed. Thus, for highly informative marker genes, this weight is close to 1, whereas for uninformative marker genes, the weight is close to zero. We use these weights as “quality scores” when inferring cell type fractions in step (iv).

When applied to a scRNA-Seq reference matrix, the above procedure results in two imputed DNAm reference matrices, one derived from each of the SCM2 and RMAP sets. In order to arrive at one unique DNAm reference matrix, we merged the two as follows. For marker genes present in both DNAm references, we averaged the DNAm values over the two reference matrices. This is justified because we always observed excellent correlation between the imputed DNAm values from the two separate imputation models. For marker genes present in one DNAm reference and not the other, we assigned the imputed DNAm values from the one where it was present. Most often, this scenario occurs when a given marker gene is present in the SCM2 dataset but not in the RMAP set, or vice versa, or because the given marker genes exhibited anti-correlation only in one of the sets. Marker genes not present in any of the two sets, or for which imputation was not possible in both sets, were discarded (equivalent to assigning a quality weight of zero).

#### Step (iv): Estimation of cell type fractions and cell type-specific differential DNAm

With the merged imputed DNAm reference obtained in previous step, we next use this reference matrix to estimate corresponding cell type fractions in a bulk sample for which a genome-wide DNAm profile is available. These bulk samples could be real tissue, or could be in silico generated, i.e., “simulated”, mixtures from purified DNAm profiles presenting the cell types in the reference matrix. In either case, given a genome-wide DNAm profile of a bulk sample, we first collapse the DNAm values so as to assign a unique DNAm value to each gene in the DNAm reference matrix. We do this as before, by taking the average DNAm of CpGs/probes within 200 bp upstream of the TSS or, if not available, by taking the average DNAm over 1st Exon CpGs/probes, following our validated procedure in the FEM algorithm [73]. Denoting by $\overrightarrow{y}$ the vector of such DNAm values for the bulk sample over the genes in the DNAm reference, and letting *M*^*(R)*^ and *W* denote the DNAm reference and quality weight matrices, respectively, we then run a weighted robust linear multivariate model, i.e., we minimize the objective function

$$ {\mathit{\min}}_{\overrightarrow{f}}\left\Vert {W}^{\frac{1}{2}}\left(\overrightarrow{y}-{M}^{(R)}\overrightarrow{f}\right)\right\Vert $$

using Huber’s robust M-estimator [77] as implemented with the *rlm* function of the *MASS* R-package. We note that *W* is by definition a diagonal matrix. The cell type fraction vector $\overrightarrow{f}$ which minimizes the above objective function are the estimated cell type fractions in the given bulk sample. While the above optimization does not result in fractions that are necessarily positive or that add to a number equal to 1, we can impose such non-negativity and normalization constraints a posteriori, by setting any inferred negative values to zero and scaling the rest so that their sum equals 1, a procedure which we and others have extensively validated [20, 30]. We note that the above model can be viewed as performing weighted robust partial correlations (RPC); hence, we refer to the estimation procedure of cell type fractions as RPC.

If we estimate cell type fractions for many bulk tissue samples in the context of an epigenome study where we are interested in identifying cell type-specific differential methylation associated with some phenotype of interest, we can achieve this using our CellDMC algorithm [24]. Briefly, CellDMC runs the following linear model, which is run separately for each CpG *c*:

$$ \overrightarrow{y_c}=\sum \limits_{k=1}^K{\mu}_{ck}\overrightarrow{\hat{f_k}}+\sum \limits_{k=1}^K{\beta}_{ck}^{(I)}\overrightarrow{\hat{f_k}}\ast \overset{\rightharpoonup }{z}+\overrightarrow{\varepsilon} $$

where $\overrightarrow{y_c}$ denotes the vector of DNAm values of CpG *c* across all bulk samples, $\overrightarrow{\hat{f_k}}$ denotes the corresponding vector of cell type fraction estimates for cell type *k* across all bulk samples, $\overset{\rightharpoonup }{z}$ denotes the phenotype of interest vector, ${\mu}_c,{\mu}_{ck},{\beta}_c,{\beta}_{ck}^{(I)}$ are regression coefficients to be estimated, and * denotes the interaction term and where we assume *K* cell types and that errors are Gaussianly distributed with a variance that may depend on the specific CpG *c*. The regression coefficients ${\beta}_{ck}^{(I)}$ inform us as to whether there is a significant interaction between the phenotype and the corresponding fraction for cell type *k*. We note that if differential methylation associated with the phenotype occurs at a CpG *c* and in cell type *k*, that the observed differential methylation should be larger in samples with high fractions for that cell type *k* compared to samples with low content for cell type *k* and should be detectable via a statistically significant interaction term ${\beta}_{ck}^{(I)}$. We solve the above model using least squares which provides estimates for the regression coefficients and their statistical significance via *P* values ${P}_{ck}^{(I)}$. The *P* values ${P}_{ck}^{(I)}$ for each cell type *k* are adjusted for multiple hypothesis testing using Benjamini-Hochberg (BH) FDR estimation. For those CpGs with BH-adjusted *P* values less than a predefined significance threshold (i.e., typically BH FDR < 0.05), we call it a DMCT (differentially methylated cell type) in the given cell type. Finally, CpGs can be ranked within each cell type according to the associated *P* value of significance. Finally, we note that additional covariates representing other biological (e.g., age, gender, ethnicity) or technical factors (batch) can be included in the above model, as described by us previously [24].

### Validation strategies for EPISCORE

Within EPISCORE, there is a need to validate both the scRNA-Seq expression and imputed DNAm reference matrices. We validate the former using independent scRNA-Seq data (or with bulk expression for purified cell types if appropriate scRNA-Seq data is not available) and using two distinct approaches. In the classification approach, we classify cells from independent scRNA-Seq experiments into one of the cell types in our reference matrix using a maximum proportion criterion, where the proportions are estimated using RPC as implemented in our EpiDISH BioC package [30, 78]. For instance, we use this procedure to validate the scRNA-Seq reference matrix derived from SmartSeq2 in corresponding 10X data. Importantly, the use of RPC has two key advantages. First, in RPC, the estimation of regression weights is carried out without imposing the non-negativity and normalization constraints (these constraints are imposed a posteriori), which renders RPC more robust to technical differences between scRNA-Seq assays, i.e., to variations in the number of dropouts and the dynamic range in gene expression which could otherwise negatively affect inference. Indeed, in RPC, the inferred regression weights are invariant to shifts in the mean and scaling of the single-cell expression profiles. Second, because in the RPC framework estimated regression weights do not need to add to 1, we can use median values in the actual reference matrices as opposed to averages, rendering the estimation and classification more robust. In the second validation approach, we use in silico mixtures, simulating bulk samples by mixing together 1000 single cells, with the proportions of each cell type drawn from an unbiased Dirichlet distribution. RPC applied to these bulk simulated samples yields estimated proportions for each cell type that we can then compare to the true proportions using Pearson correlation coefficients and/or *R*^2^ values.

In the case of DNAm, single-cell DNAm profiles are too sparse and not generally available, so for validation, one needs to use bulk DNAm profiles of high cellular purity representing cell types that are as similar as possible to those in the imputed DNAm reference matrix. These purified DNAm profiles can then be mixed together in known proportions using a Dirichlet distribution to simulate bulk tissue DNAm profiles. In this case, we run the weighted RPC to infer cell type fractions and compare these to the true proportions using Pearson correlation coefficients and/or *R*^2^ values. We note that purified samples corresponding to relevant cell types are not always available, which thus requires the use of sensible proxies. In the case of lung tissue, for epithelial and fibroblast cells, we used corresponding epithelial and fibroblast cell lines from ENCODE [32]. For immune cells, we use the FACS sorted purified blood cell subtype samples from Reinius et al. [67]. For lung endothelial cells, we used pulmonary endothelial cells (PECs) from Hautefort et al. [69]. We note that before generating in silico mixtures, we removed samples that appeared to be outliers or which did not appear to be pure. For instance, of the 18 PECs, one was not predicted to be of endothelial origin and was therefore removed. In the case of cell lines, it is well known that many have been misidentified or misclassified [79], and so we removed those whose predicted purity was not over 60%. A similar procedure was used for the breast DNAm reference matrices. While we acknowledge that excluding specific outliers from the in silico mixtures could result in inflated performance measures, not removing misclassified cell lines could equally result in reporting overly pessimistic performance. In the case of DNAm, the strongest validation thus derives from the application of CellDMC to specific phenotypes where there is prior biological truth as to which DNAm changes are expected to occur in which cell types. For instance, it is well known that tissue-specific transcription factors and PRC2-marked genes generally undergo hypermethylation at their CpG-rich promoters in cancer epithelial cells [42, 45, 80]. Thus, the DNAm references can be indirectly validated by studying the predictions for DMCTs derived from CellDMC. In the context of breast cancer, there are two well-known epithelial subtypes: triple-negative and ER+. Breast cancer cell lines and breast cancer tissue classified into these two subtypes are available in sufficiently large numbers. Hence, here we can define a gold standard list of epithelial DMCTs between the two subtypes of breast cancer using the pure epithelial cell lines and subsequently test the DNAm reference and CellDMC in their ability to detect such DMCTs in the epithelial compartment of corresponding breast cancer tissue samples.

### GSEA and ChIP-Seq analysis

Briefly, gene set enrichment analysis was performed using the ebGSEA package (non-regression version) [39] and the MSigDB database [38]. Briefly, from a list of statistically significant CpGs, the non-regression version of ebGSEA maps these to a list of significantly-associated genes, by adjusting for the number of probes on the Illumina DNAm beadarray that map to a given gene. For this list of associated genes, GSEA is subsequently performed using a one-tailed Fisher test. *P* values are adjusted for multiple testing using Benjamini-Hochberg procedure. In the case of the ChIP-Seq data, we downloaded the SMAD2 and SMAD3 predicted binding target files from http://chip-atlas.org/ [56] for all 3 choices of window size: ± 1 kb, 5 kb, and 10 kb centered on TSS of a gene. For SMAD2, ChIP-Seq data was available in an endothelial cell line, so for SMAD2, we performed two separate analysis: (i) averaging the gene-assigned binding intensity values over the HUVEC cell line experiments and (ii) averaging the gene-assigned binding intensity values over all cell lines not including hESCs and embryonic ones, as for these ChIP-Seq profiles may be far less representative of those found in adult cells and tissues. For SMAD3, no data in endothelial cells was available and we only performed the second analysis. Because the number of genes bound by SMAD2/SMAD3 in at least one samples was quite high (with highly variable peak binding intensity values), we decided that a threshold independent analysis was necessary. To this end, we compared the binding intensity values for genes associated with specific CpGs of interest (in our case these will be hyper or hypomethylated DMCTs) to those of genes not associated with any DMCTs. The comparison was performed using a one-tailed Wilcoxon rank sum test.

### Application of EpiSCORE to peripheral blood mononuclear cells

*Brief description of bulk expression datasets used:*

- Watkins et al. [81]: A dataset containing bulk mRNA expression profiles of purified blood cell subtypes, generated using Illumina HumanWG-6 expression BeadChips. The set contains expression profiles for 7 CD14+ monocyte, 7 B cell, 7 CD4+ T cell, 7 NK, and 7 CD8+ T cell samples and is available from the Haemosphere project (https://haemosphere.org/datasets/show). Library size normalization and log2 transformed data is available from this site.
- Haemopedia-Human [82]: A dataset containing bulk RNA-Seq profiles (Illumina HiSeq) of purified blood cell subtypes, including 7 CD14+ monocyte samples (5 classic monocytes and 2 non-classic), 8 CD19+ B cell samples (3 memory and 5 naïve), 5 CD4+ T cell samples, 4 CD56+ NK samples, and 4 CD8+ T cells samples. This dataset is also available from Haemosphere (https://haemosphere.org/datasets/show). TPM-normalized data was available from website and was further transformed as log2(TPM + 1) for downstream analysis.
- Monaco et al. [83]: This is a bulk RNA-Seq dataset of purified blood cell subtypes, available from GEO (GSE107011), containing expression profiles for 29 blood cell subtypes, including CD14+ monocytes, CD19+ B cells, CD4+ T cells, CD56+ NK cells, and CD8+ T cells. We downloaded the TPM-normalized expression matrix, which were further transformed as log2(TPM + 1) for downstream analysis.
- Salas et al. [23]: This data set was profiled with Illumina MethylationEPIC beadarrays and consists of immunomagnetic sorted adult blood cell populations, consisting of 37 magnetic sorted purified blood cell and 12 mixture samples reconstructed using fixed amounts of DNA from purified cells. Raw data is available from FlowSorted.Blood.EPIC package, formatted as an RGChannelSet. Six immune cell subtypes (CD4+ T cell, CD8+ T cell, CD19+ B cells, CD14+ monocytes, CD56+ NK cells, and granulocytes) were profiled. We used the 12 experimental mixtures with known cell type fractions for evaluation of the EpiSCORE PBMC DNAm reference. RGChannelSet data was processed with minfi. Illumina definition of beta value was used. Probes with > 25% failed samples defined by *p* > 0.01 comparing to negative control positions were discarded, resulting in 865,986 probes. The remaining failed positions were imputed with knn (*k* = 5). The beta value of all the samples is normalized with BMIQ before analysis.
- Koestler et al. [22]: This data set was profiled with Illumina HM450k beadarrays and consists of 6 whole blood samples with matched FACS cell counts and 12 whole blood experimental mixtures, generating from purified blood cell subtypes. This dataset was normalized as described by us previously [30].

#### Dimensional reduction and clustering of scRNA-Seq data

To construct the scRNA-Seq expression reference for peripheral blood mononuclear cells (PBMCs), we processed the scRNA-seq UMI count matrix from Zheng et al. (PBMC68k 10X dataset) (https://github.com/10XGenomics/single-cell-3prime-paper/tree/master/pbmc68k_analysis) [59], which profiled almost 70,000 PBMCs. Cells expressing at least 400 genes and with mitochondria gene counts less than 5% were retained. We normalized the UMI count matrix by first dividing the total counts of each cell and then multiplying with the maximum total counts across all cells, to yield UMI’. Then, the count matrix was transformed according to log2(UMI’ + 1). Based on a plot of the standard deviation (SD) vs. the mean (AV), we selected 3259 variable genes according to AV > 0.1 and SD/AV > 0.25, as estimated across cells. Then, PCA was applied on the 3259 variable genes, followed by t-SNE [84] analysis with the top 50 PCs as input, which projected the log2 normalized count matrix to a two-dimensional embedding. DBSCAN algorithm [85] was then used to cluster the cells in the t-SNE space. t-SNE was implemented using Rtsne function in *Rtsne* R-package (perplexity = 50, pca = T). DBSCAN was run with dbscan function in *dbscan* R-package (eps = 0.5, minPts = 60).

#### Construction of bulk RNA-Seq expression reference and annotation of single-cell clusters

In order to annotate the clusters of cells obtained in the previous step, we constructed a bulk RNA-Seq expression reference matrix using data from Monaco et al. (GSE107011) [83]. We analyzed the TPM-normalized expression matrix as provided by the authors. TPM values were transformed into a log2(TPM + 1) scale. The 29 blood cell subtypes described there were categorized into 5 main cell types, which included CD14+ monocytes, CD19+ B cells, CD4+ T cells, CD56+ NK cells, and CD8+ T cells. Cell types not included in the 5 main categories were discarded. Differential expression analysis was performed for each of these cell types by comparison to all other 4 cell types using a Wilcoxon rank sum test. Differentially expressed genes (DEGs) were declared using a FDR < 0.05 threshold (with *P* values as given by the Wilcoxon test) and in addition by requiring a log2[fold change] > 2, i.e., a stringent 4-fold change in expression. In order to improve the specificity of the DEGs for each given cell type, genes with a log2[fold change] difference < 0.2 with the second highest expressing cell type were removed. Finally, the bulk RNA-seq expression reference matrix for the 6 PBMC cell types was obtained by taking the average expression level of log2(TPM + 1) values. This reference matrix was subsequently validated using bulk RNA-Seq and microarray expression data of purified blood cell subtypes from Haemopedia-Human-RNASeq [82] and Watkins et al. [81]. This validation was done by generating in silico mixtures with uniform Dirichlet weights of the 5 relevant PBMC cell types and then applying robust partial correlations (RPCs) [20, 30] to each mixture using the constructed bulk RNA-Seq expression reference. Estimated vs. true cell type fractions were compared to validate the reference. Finally, having validated the bulk RNA-Seq expression reference for the 5 PBMC cell types, we then used this reference to annotate the single cells from the PBMC68k 10X study. This was done by regressing each scRNA-Seq profile against the bulk RNA-Seq reference matrix in a robust multivariate model, i.e., effectively running again RPCs. The predicted cell type was defined as the cell type with the highest estimated fraction among the 5 cell types (CD14+ monocyte, CD19+ B, CD4+ T cell, CD56+ NK, and CD8+ T cell). Clusters where more than 80% of cells were predicted to be of the same cell type (“dominant cell type”) were retained and annotated to that dominant cell type. Clusters without a dominant cell type were discarded. Cells mapping to a cluster with a dominant cell type but which did not classify into that dominant cell type were discarded. Clusters with the same dominant cell type were merged, resulting in 5 major clusters: a 1577 CD14+ monocyte cluster, a 2796 CD19+ B cell cluster, a 4308 CD4+ T cell cluster, a 2750 CD56+ NK cluster, and a 4496 CD8+ T cell cluster.

#### Construction and validation of single-cell mRNA expression reference for PBMCs

Having annotated the single-cell clusters in the PBMC 10X dataset in terms of the main 5 PBMC cell types, we next performed differential expression analysis between these 5 PBMC cell types. This was done for each cell type separately by comparison to all other 4 cell types. Differentially expressed genes (DEGs) were identified using a Wilcoxon test with FDR < 0.05, and further selected according to log2(fold change) > 0.5, and by demanding that the expression in the given cell type is highest among all cell types (we note that the Wilcoxon test does not necessarily guarantee this requirement). For each cell type, resulting genes were then ranked by decreasing log2(FC). The top 20 marker genes from each cell type were combined into a reference matrix. The number of marker genes was chosen according to the minimum number of significant genes among 5 cell types, in order to balance the number of marker genes of each cell type, resulting in 96 unique marker genes. The average expression level of the selected marker genes in each cell type was used in the reference matrix. This scRNA-Seq expression reference was validated using in silico mixtures of bulk RNA-Seq profiles representing purified blood cell subtypes derived from the Haemosphere-human dataset.

#### Imputation and validation of DNAm reference matrix for peripheral blood

We aimed to impute a DNAm reference matrix for peripheral blood directly from the scRNA-Seq expression reference constructed as described in previous sections. The imputation involves two steps: (i) identification of a subset of marker genes in the scRNA-Seq reference matrix for which their differential expression across PBMC cell types is explained by corresponding differential DNAm at regulatory elements associated with these genes and (ii) imputation of DNAm at these regulatory elements in the PBMC cell types using as input the expression value from the scRNA-Seq PBMC reference matrix.

- *Preliminary processing*: In order to identify the subset of marker genes where DNAm variation associates with corresponding differential expression, we require independent DNAm and mRNA expression data of purified blood cell subtypes. To this end, we obtained WGBS data from BLUEPRINT (BP), specifically the hg38 blood samples from IHEC data portal (https://epigenomesportal.ca/ihec/grid.html). The LiftOver tool from the UCSC Genome Browser was used to convert gene coordinates and annotation files from version hg38 to hg19. We kept CpGs with read coverage ≥ 20 and removed CpGs or samples where the missing fraction was equal or more than 30%. In total, there were 12 blood cell subtypes in BP for which we could also find corresponding bulk RNA-Seq profiles, using the data from Monaco et al. [83]. These cell types were central memory CD8 T cell, effector memory CD8 T cell, terminal effector CD8 T cell, regulatory T cell, naïve B cell, switched memory B cell, non-switched memory B cell, classical monocytes, natural killer, dendritic cells, neutrophils, and terminal effector CD4 T cell. The final beta value DNAm matrix was constructed by taking the average beta value across samples of the same cell type. Because later we want to validate our imputed DNAm reference matrix, we only kept CpGs that overlap with Illumina450k CpGs. Coordinate information of CpG was from Illumina450k annotation file in IlluminaHumanMethylation450kanno.ilmn12.hg19 package. We also only retained CpGs which exhibited a DNAm range across the 12 samples larger than 0.5. This was done to ensure that later, when correlating to gene-expression, that significant associations involve a reasonable range in DNAm change. As far as the mRNA expression data is concerned, we used the normalized log2(TPM + 1) values from Monaco et al., which included the same 12 cell types as in the WGBS beta matrix. Finally, we averaged expression across all the samples of each cell type. Only variable genes exhibiting an expression range > 1 were chosen for the downstream correlation analyses. This particular expression range was chosen because the difference in expression between the non-expressed and expressed genes in Monaco et al. was approximately 1, and therefore, this filtering would help identify correlations with DNAm where differences in expression are likely to be more biologically meaningful.
- *DNAm and gene expression association analysis*: To each variable gene, we assigned CpGs mapping within a 1 Mb window centered at the transcription start site (TSS) of the gene. In more detail, we only kept CpGs that mapped + 500 kb upstream or downstream of the TSS, but also excluding CpGs mapping within 5 kb on either side of the TSS. This was done because it is well known that distal enhancers are among the regulatory elements showing most variation in DNAm and in association with gene-expression. Thus, for each variable gene (expression range > 1), we then performed linear regressions against each variable CpG (DNAm range > 0.5) across the 12 blood cell subtypes. CpG-gene pairs with FDR < 0.05 and *R*^2^ > 0.8 were considered significant and kept for further analysis. Among these CpG-gene pairs, if a CpG mapped to multiple genes, we assigned the CpG to the gene with the largest absolute *t* statistic value.
- *Imputation and validation of PBMC DNA methylation reference from single-cell expression reference*: First, we *z*-score normalized each gene’s expression profile across the 12 blood samples before fitting a logistic regression to the DNAm beta values of the variable CpGs. This was done for all significant CpG-gene pairs as generated in previous step. To impute a DNAm value from scRNA-Seq reference, we first *z*-score normalized each gene in the scRNA-Seq reference, and then used these values as input to the logistic fit to obtain the corresponding DNAm estimate. Finally, we validated the imputed DNA methylation reference matrix using independent Illumina 450k DNAm data of purified blood cell subtypes (only the PBMCs) from Reinius et al. [67]. This was done using in silico mixtures with uniform Dirichlet weights, and comparing estimated to true cell type fractions, where estimated cell type fractions were obtained using EpiDISH (robust partial correlations—RPCs) [30] with the imputed DNAm reference matrix. RPCs implement a robust multivariate linear regression estimator, regressing each sample (e.g., a mixture) against the imputed DNAm reference profiles for each cell type. It is performed using a robust M-estimator, as implemented in the *rlm* function of the *MASS* R-package. A posterior constraint is imposed where all negative regression weights were set to 0, and subsequently, all weights normalized to sum up to 1. These weights represent the estimated cell type fractions in the sample. We also repeated the in silico mixture analysis, but this time using the weights of the Dirichlet distribution to match those seen in real blood data [86], that is, weights were chosen so as to match the mean cell type fraction as observed in PBMC tissue, and a global scale factor was chosen to match the observed variance across all cell types.
- *Evaluation of DNA methylation PBMC reference in whole blood DNAm dataset with known cell type fractions*: Both 450k data from Koestler et al. and EPIC data from Salas et al. have known fraction of 6 cell types, which are CD4+ T cell, CD8+ T cell, CD19+ B cells, CD14+ monocytes, CD56+ NK cells, and granulocytes. Because the EPISCORE reference does not include granulocytes (only monocytes, B cells, CD4 T cells, NK and CD8 T cells), the FACs fraction of monocytes is counted as the sum of monocytes and granulocytes. We used RPC with EPISCORE DNAm PBMC reference to estimate cell type fractions with epidish function in EpiDISH package. As a benchmark, we also used the EpiDISH DNAm whole blood reference available in EpiDISH package with data(“centDHSbloodDMC.m”), which profiled 7 immune cell subtypes consisting of monocytes, B cells, CD4 T cells, NK, CD8 T cells, neutrophils, and eosinophils. To compare EpiSCORE and EpiDISH references in an unbiased way, we summed up the neutrophil, eosinophil, and monocyte proportions from the EpiDISH reference to define a total monocyte fraction. Performance of the two methods was assessed using R square and RMSE.

## Supplementary information

**Additional file 1.** Supplementary Figures. A pdf document containing all Supplementary Figures.

**Additional file 2.** Database data. An .Rd. data object file to be read in with R, containing the matched mRNA expression and DNA methylation data matrices for each of the two databases (Stem-cell matrix compendium-SCM2, and Epigenomics Roadmap-RMAP).

**Additional file 3.** EPISCORE DNAm reference for lung tissue. An .Rd. data object file, containing the EPISCORE DNAm reference matrices derived from SCM2, RMAP and the final merged one.

**Additional file 4.** EPISCORE DNAm reference for breast tissue. An .Rd. data object file, containing the EPISCORE DNAm reference matrices derived from SCM2, RMAP and the final merged one.

**Additional file 5.** Gold-standard differentially methylated CpGs between breast cancer subtypes. An .Rd. data object file containing a matrix listing differentially methylated CpGs between triple negative estrogen receptor negative and estrogen-receptor positive breast cancer.

**Additional file 6.** Review history.

## Data Availability

Data analyzed in this manuscript is already publicly available from GEO (www.ncbi.nlm.nih.gov/geo/) under accession numbers GSE69914 [47], GSE35069 [67], GSE68379 [58], GSE40699 [87], GSE84395 [69], GSE74877 [70], and GSE56719 [71], from ArrayExpress (www.ebi.ac.uk/arrayexpress) under accession number E-MTAB-6149 [28], from the Chan-Zuckerberg Biohub https://tabula-muris.ds.czbiohub.org [12], and from TCGA data portal https://portal.gdc.cancer.gov/ [37, 55]. EPISCORE [27, 88] is freely available as an R-package from https://github.com/aet21/EpiSCOREunder a GPL-2 license, or from 10.5281/zenodo.3893646 under a Creative Commons Attribution 4.0 International Public License (“Public License”). The R package comes with a vignette and tutorial, sample datasets and a reference manual.
