# Supplementary material for: EPISCORE: cell type deconvolution of bulk tissue DNA methylomes from single-cell RNA-Seq data
Source: Genome Biol. 2020 Sep 4;21:221. doi: 10.1186/s13059-020-02126-9 (PMC7650528; doi:10.1186/s13059-020-02126-9)
Supplement: Supplementary file 1 — Additional file 1. Supplementary Figures. A pdf document containing all Supplementary Figures. [file 13059_2020_2126_MOESM1_ESM.pdf]

Supplementary Information for "Cell-type deconvolution of bulk tissue DNA methylomes from single-cell RNA-Seq data"

SUPPLEMENTARY FIGURES

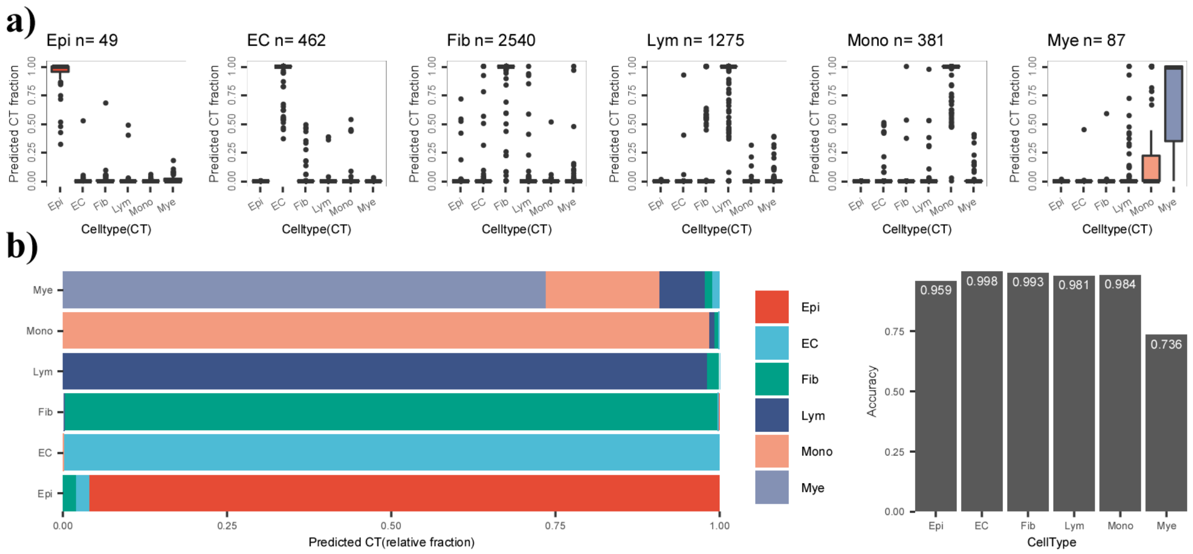

Fig.S1: Validation of lung-tissue scRNA-Seq reference matrix at resolution of 6 cell-types. a) Boxplots of estimated cell-type fractions for single-cells from the lung MCA1 10X atlas, which were annotated as epithelial (Epi), endothelial (Endo), fibroblast (Fib), lymphocyte (B-cell, T-cell or NK-cell), monocyte (Mono) or myeloid (Mye). The number of single cells annotated to each cell-type is given. Cell-type fractions were estimated using RPC with an expression reference defined over 6 cell-types (epithelial, endothelial, fibroblast, lymphocyte, monocyte and myeloid) using the SmartSeq2 scRNA-Seq data from MCA1. **b)** Left: Barplots depicting the relative fractions of single-cells annotated to each type (labeled on y-axis) which were predicted to be epithelial, endothelial, fibroblast, lymphocyte, monocyte or myeloid cell (labeled by different colors) based on the cell-type fraction estimates obtained in a). Right: barplot displaying the overall accuracy of using RPC+expression reference to correctly classify each cell type.

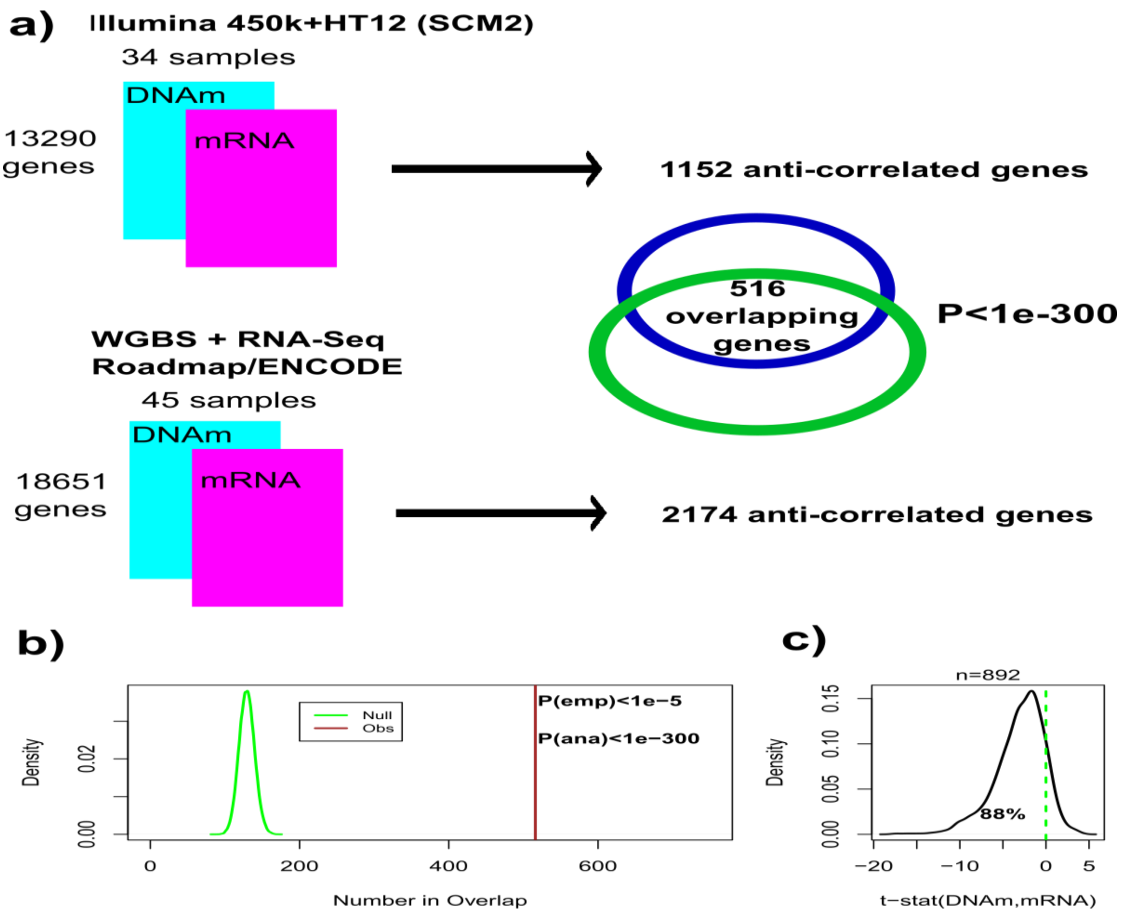

Fig.S2: DNAm and mRNA expression anti-correlated gene subsets are robust. a) Illumina array and sequencing based matched DNAm-mRNA expression sets from the Stem-Cell-Matrix Compendium-2 (SCM2) and the NIH Epigenomics Roadmap/ENCODE, respectively, were used to identify genes with significant associations between their promoter DNAm levels and gene expression. Over 80% of these exhibited anticorrelations, and there was a remarkable overlap (516 overlapping genes) between those derived from the two separate matched sets, as shown. P-value derives from a normal approximation to the null, as derived empirically through Monte-Carlo analysis (100,000 randomizations). **b)** Density plot of the observed overlap numbers obtained by randomly selecting genes from each matched set, thus defining the null distribution (green curve). Vertical line in brown indicates the observed overlap number. Empirical as well as analytical (normal approximation) P-values are given. **c)** Density distribution of t-statistics derived from a linear regression between promoter DNAm and gene expression in the WGBS/RNA-Seq set, for 892 genes which exhibited significant variance in DNAm and gene expression and which had been selected as exhibiting a significant anti-correlation in the array-based matched set. The percentage of these 892 genes which exhibited a negative correlation in the sequencing set is given.

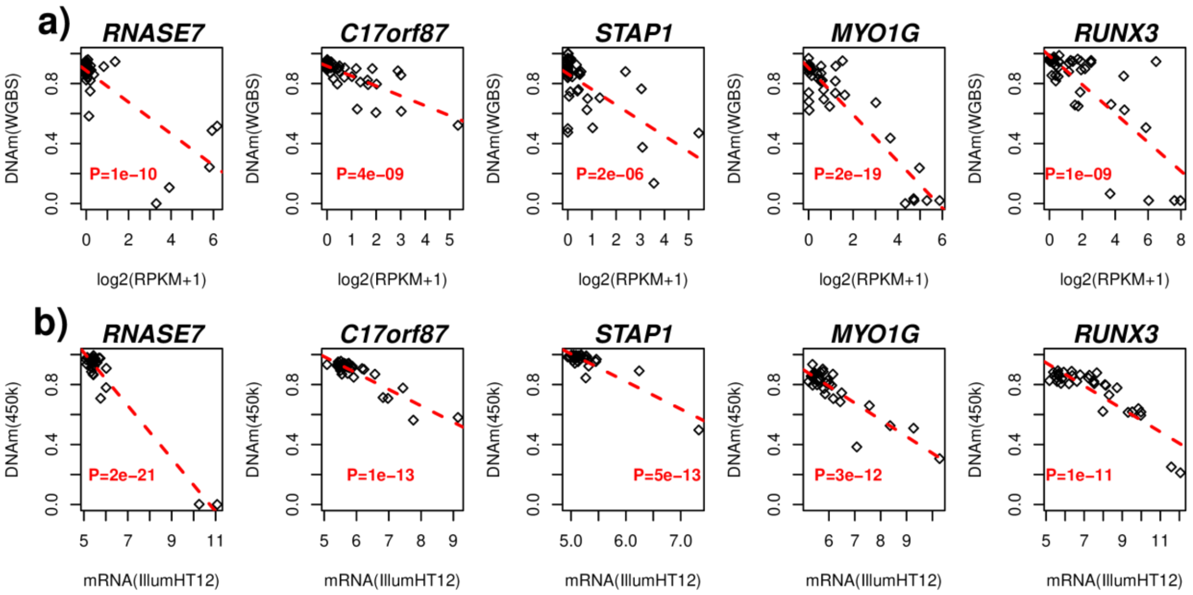

Fig.S3: Example profiles of anti-correlated genes. a) Scatterplot of promoter DNAm level (y-axis) vs. gene expression (x-axis) for 5 genes and 45 samples with matched DNAm/gene expression data derived from the NIH Epigenomics Roadmap/ENCODE. Estimated regression line and P-value are given. **b)** As a), but now for 34 samples with matched Illumina array DNAm-mRNA expression from the Stem-Cell-Matrix Compendium-2 (SCM2).

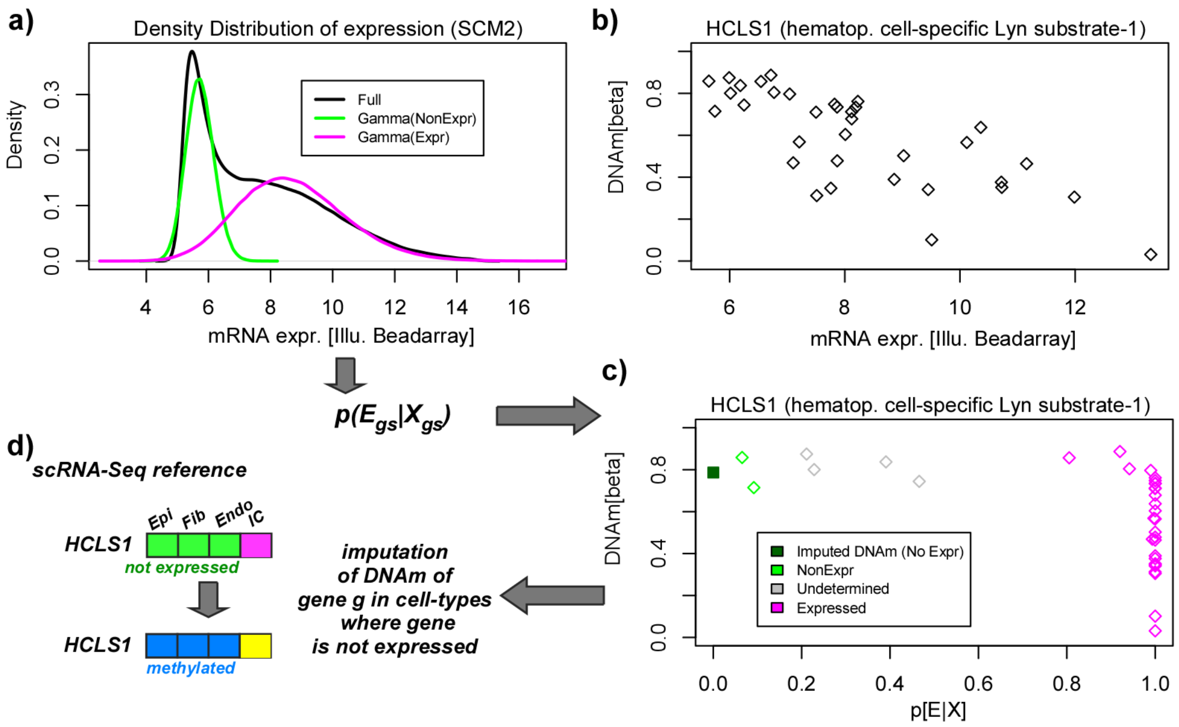

Fig.S4: Example of imputation of DNAm at a marker gene. a) Density distribution of normalized expression values in the SCM2 database across all genes and samples (black line) with a 2-component Gamma-mixture model fit displayed in green (non-expressed state) and magenta (expressed state). **b)** An example of a gene (HCLS1) which exhibits strong anti-correlation between promoter DNAm and gene-expression as assessed in the SCM2 database. **c)** As b), but now plotting DNAm (y-axis) versus the probability of being expressed (E) given the expression level X, i.e. p(E|X). This probability is obtained via Bayes theorem, as the posterior probability from the 2-component Gamma-mixture model fit in step-a). This plot allows imputation of DNAm at the gene's promoter for samples not expressing the given gene, as indicated. **d)** Illustration of how the expression levels derived from the scRNA-Seq tissue-atlas are mapped to corresponding DNAm levels, where the imputation proceeds for the samples not expressing the given gene. In the case of HCLS1, an immune-cell marker gene, no expression is associated with relatively high promoter methylation.

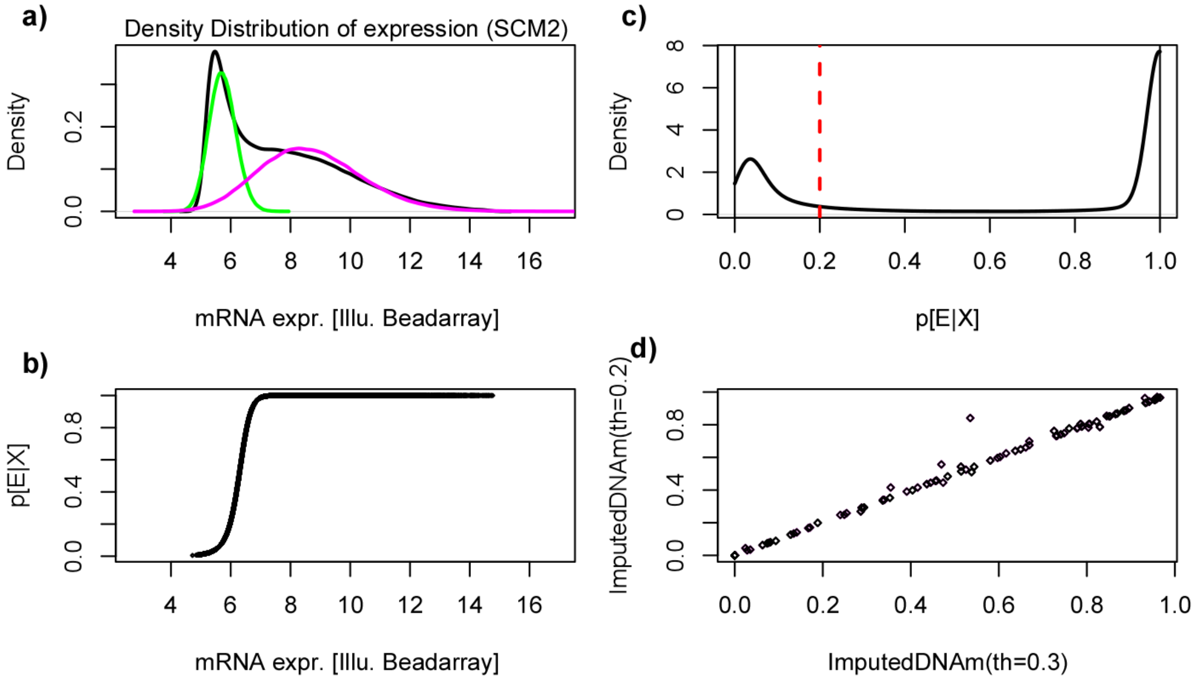

Fig.S5: Robustness of DNAm imputation to expression significance threshold. a) Density distribution of normalized expression values in the SCM2 database across all genes and samples (black line) with a 2-component Gamma-mixture model fit displayed in green (non-expressed state) and magenta (expressed state). **b)** Scatterplot displaying the probability of being expressed given the expression level (p[E|X], y-axis) vs. the expression level (log2-expression, Illumina Beadarray, x-axis). **c)** The density distribution of p[E|X], revealing the bi-modality. Vertical red dashed line indicates the threshold (p[E|X]=0.2) used to declare a gene in a given sample to be non-expressed. **d)** Scatterplot of the imputed DNAm values using two different thresholds for declaring the non-expressed state: p[E|X] < 0.3 (x-axis), vs. p[E|X] < 0.2 (y-axis).

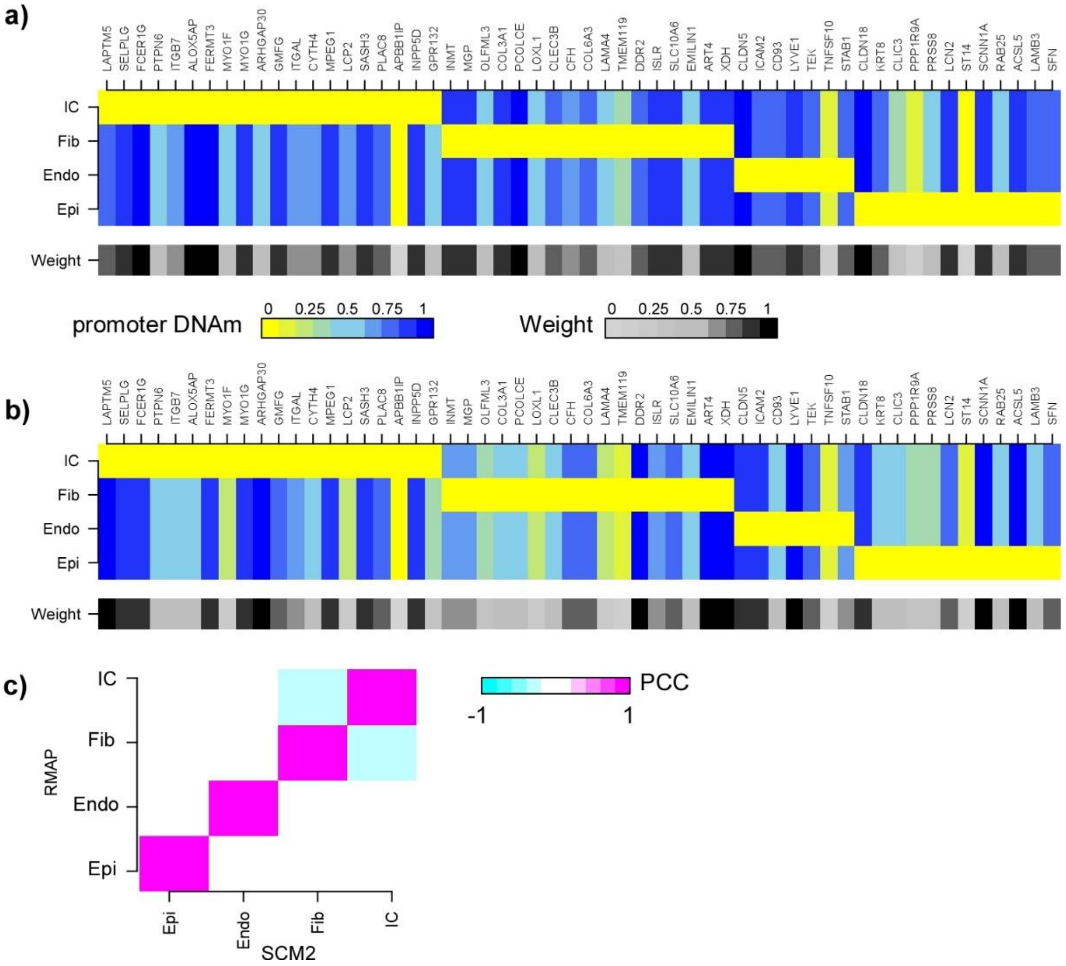

Fig.S6: Imputed DNAm references for lung. a) Heatmap of imputed promoter DNAm levels for 56 core anti-correlated genes from the scRNA-Seq lung tissue reference matrix, as inferred using matched bulk RNA-Seq and WGBS data from the Roadmap (RMAP), and for the 4 major cell-types found in lung tissue. Also shown is the weight as a quality metric, with higher weights indicating genes for which promoter DNAm is more informative of differential gene expression. **b)** As a), but now having imputed the DNAm data using matched Illumina beadarray mRNA expression and DNAm data from the Stem-Cell-Matrix Compendium-2 (SCM2). The heatmaps in a) & b) are displayed only for the common set of 56 genes. **c)** Heatmap of Pearson Correlation Coefficients between the RMAP and SCM2 DNAm reference matrices.

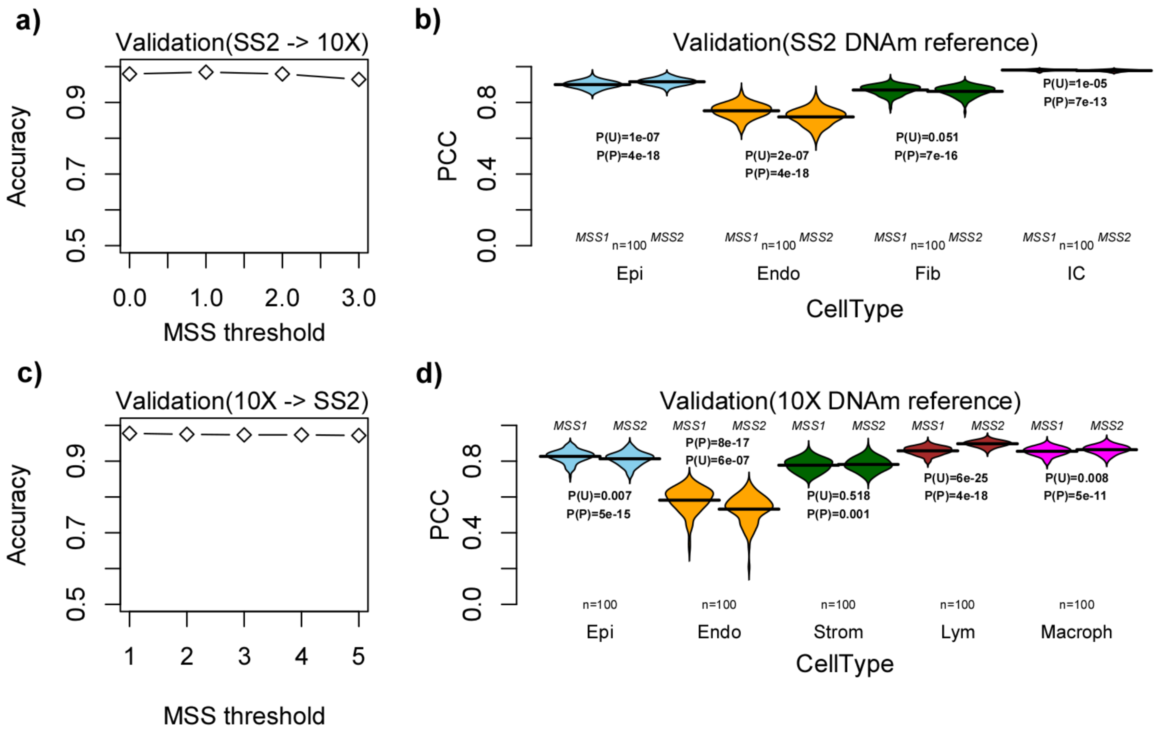

Fig.S7: Robustness of results to choice of MSS-thresholds. a) Classification accuracy (y-axis) of the scRNA-Seq reference matrix derived from the SmartSeq2 (SS2) lung Mouse Cell Atlas dataset in the corresponding scRNA-Seq 10X lung set, as a function of the marker-specificity score (MSS) threshold. MSS=m (m=0,1,2,3) means that marker genes were required to not only be more highly expressed in one cell-type compared to all others, but that the given gene is also not expressed in at least m of the other cell-types. e.g. MSS=3 means that the marker genes are not expressed in all 3 of the other cell-types, whereas MSS=1 means that a marker gene is allowed to be expressed in 2 of the other 3 cell-types. We use the same MSS-threshold for each cell-type. **b)** Violin plots displaying the Pearson Correlation Coefficient (PCC) between the true cell-type fractions and the estimated ones from the EpiSCORE DNAm reference matrix, as derived across 100 in-silico mixtures, and for a total of 100 Monte-Carlo simulations. Here MSS1 means MSS=3 for all 4 cell-types, whereas MSS2 means MSS=2 for all 4 cell-types. We provide unpaired (U) and paired (P) two-tailed Wilcoxon rank sum test P-values, as estimated over the 100 Monte-Carlo runs. **c)** As a), but for the case of the SmartSeq2 and 10X breast mouse cell atlas datasets. **d)** As b), but in the case of breast, the scRNA-Seq reference matix was constructed using MSS1=(5,5,4,4,3,3) for luminal, basal, endothelial, fibroblast, lymphocytes and macrophages, respectively, and MSS2=5 for all 6 cell-types. We note that in the validation of the DNAm reference matrix, there are no sufficient normal luminal and basal DNAm profiles in the public domain, which is why validation of the in-silico mixtures is carried out at the level of total epithelial cells, i.e. estimated basal and luminal fractions were summed and compared to the true epithelial fraction in the simulation.

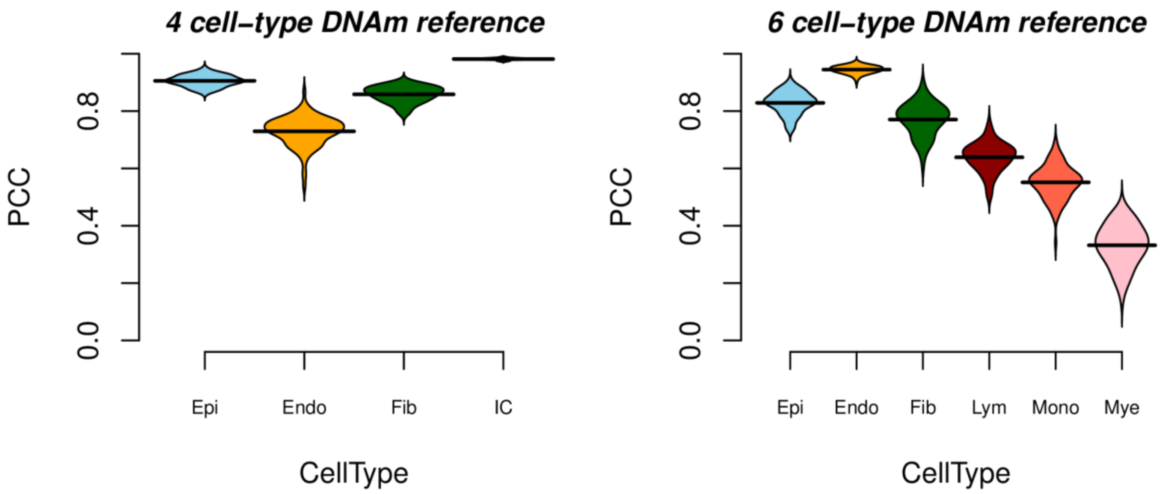

Fig.S8: Performance of EpiSCORE lung DNAm reference as a function of cellular resolution. Violin plots of the Pearson Correlation Coefficient (PCC, y-axis) vs. cell-type (x-axis), where the PCC is estimated by computing the correlation between the estimated cell-type fraction and the true fraction over 100 in-silico mixtures. Each violin plot contains 100 datapoints corresponding to 100 Monte-Carlo runs. Left panel depicts the case where the EpiSCORE DNAm reference for lung tissue is constructed over 4 main cell-types (Epithelial, Endothelial, Fibroblast and Immune-Cell), whereas right panel depicts the case where the EpiSCORE DNAm reference is constructed over 6 cell-types (Epithelial, Endothelial, Fibroblast, Lymphocyte, Monocyte and Myeloid).

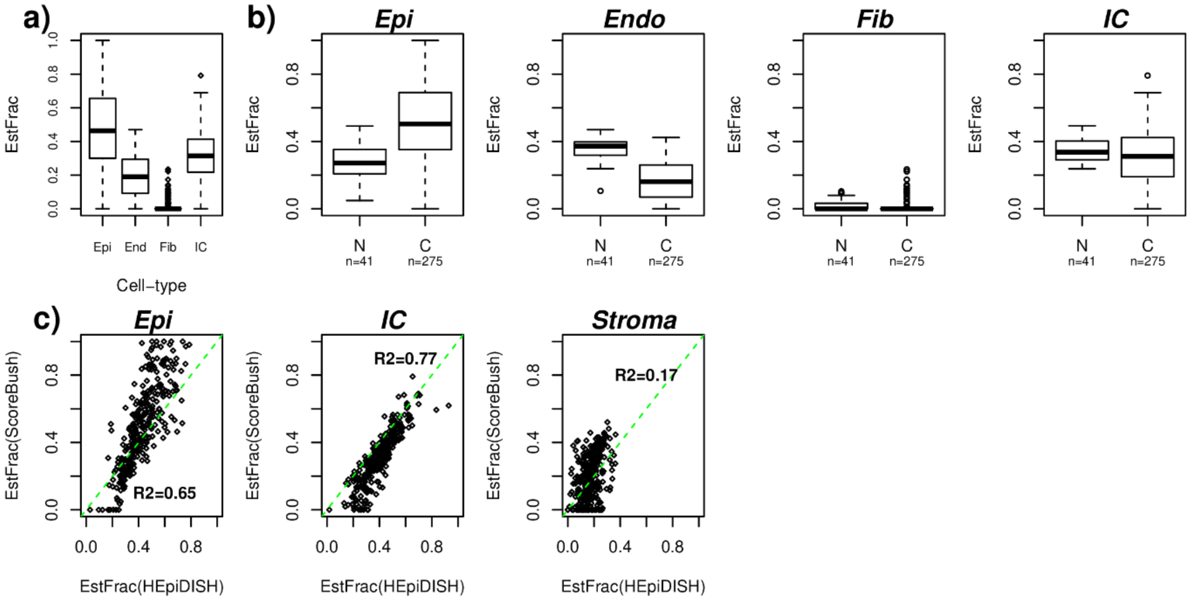

Fig.S9: EPISCORE cell-type fraction estimates in the lung squamous cell carcinoma TCGA set . a) Boxplot of cell-type fractions for the four main cell-types in lung tissue, as obtained using RPCs with our EPISCORE DNAm reference. Each boxplot is over 316 samples (41 normal + 275 cancer). **b)** Boxplot of cell-type fractions comparing normal to cancer tissue for each cell-type separately, as indicated. **c)** Scatterplot of estimated cell-type fractions obtained using HEpiDISH (x-axis) against those obtained using EPISCORE (y-axis) for the epithelial, immune cell and non-immune stromal components, as indicated. We note that since the HEpiDISH DNAm reference does not contain an endothelial component, that in the last panel we are correlating the endothelial+fibroblast component from EPISCORE to the fibroblast component of HEpiDISH. R^2 values are given in each panel.

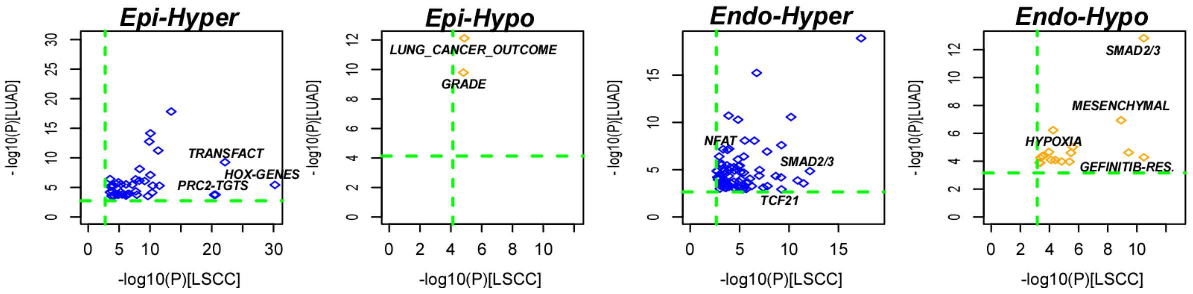

Fig.S10: Validation of EPISCORE+CellDMC GSEA results in LUAD TCGA. For the four categories of DMCTs for which we observed significant enrichment (BH-adjusted P<0.05) for biological terms in the LSCC TCGA cohort, we plot correspondingly enriched biological terms among the corresponding DMCTs identified in the LUAD TCGA cohort. Each panel is a scatterplot of the -$\log_{10}$ enrichment P-values (from a one-tailed Fisher-test) in LSCC (x-axis) vs. the corresponding values in LUAD (y-axis). Some of the biological interesting terms are labeled.

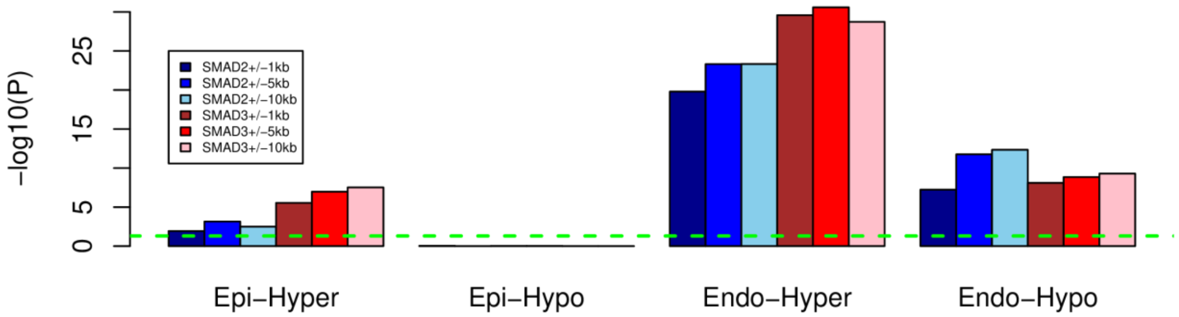

Fig.S11: SMAD2/3 binding intensity among lung cancer DMCT genes. Barplots display -log10[P-values] of a one-tailed Wilcoxon rank sum test comparing SMAD2/3 binding intensity values at peaks associated with lung cancer DMCT genes vs. genes not associated with any DMCTs. Results are shown for SMAD2 and SMAD3 binding targets defined at 3 different window sizes (+/-1kb, +/-5kb, +/-10kb). Green dashed line denotes the line P=0.05. The overall binding intensity for SMAD2 was obtained by taking the average ChIP-Seq signal intensity over Aortic smooth muscle cells, hematopoietic stem cells, human umbilical veil endothelial cells and pancreatic cells. For SMAD3, we took the average over the following cell-lines: A549, BJ, HCASMC, LX2, MDA-MB-231, NCI.H441, PC3, SUM-159PT, WHIM12.

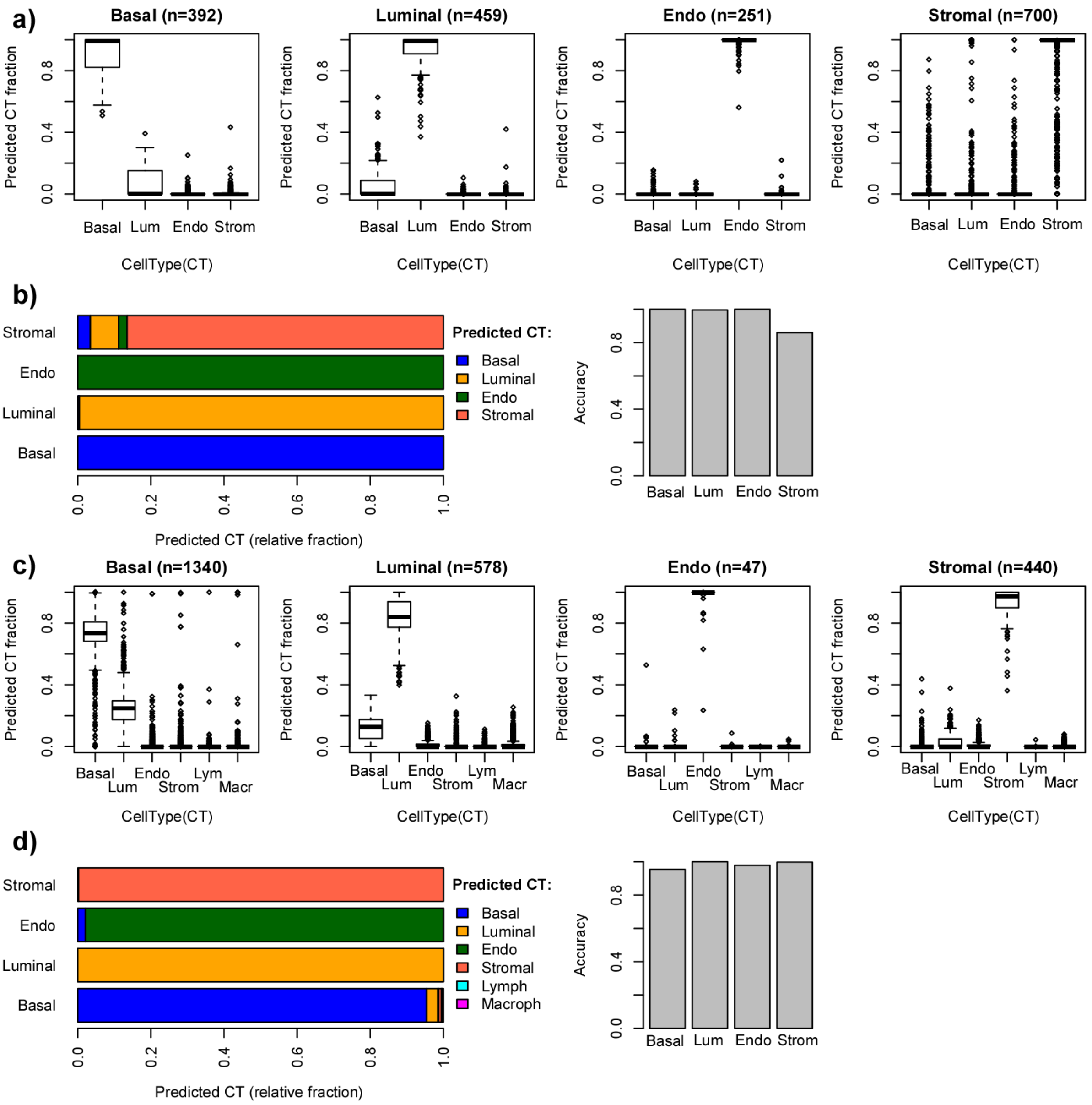

Fig.S12: Validation of breast tissue scRNA-Seq reference matrices. a) Validation of the scRNA-Seq reference matrix derived from the breast SmartSeq2 Mouse Cell Atlas and encompassing 4 cell-types (Basal, Luminal, Endothelial and Stromal) in the corresponding 10X breast mouse cell atlas set. Each panel displays boxplots of the estimated cell-type fractions for the cells in the 10X study annotated as Basal, Luminal, Endothelial and Stromal. The number of cells annotated to each cell-type in 10X is given above plot. X-axis labels the predicted cell-type using the scRNA-Seq reference matrix derived from the SmartSeq2 data and using Robust Partial Correlations to obtain the proportions. **b)** Left barplot displays corresponding classification into the cell-types in the reference matrix using a maximum fraction criterion. Right barplot displays the overall accuracy of each annotated cell-type in the 10X being classified into that cell-type. **c-d)** Same as a-b), but now validating the scRNA-Seq reference matrix derived from 10X in the SmartSeq2 data. Note that the scRNA-Seq reference matrix derived from the 10X data contains 6 cell-types (Basal, Luminal, Endothelial, Stromal, Lymphocytes and Macrophages).

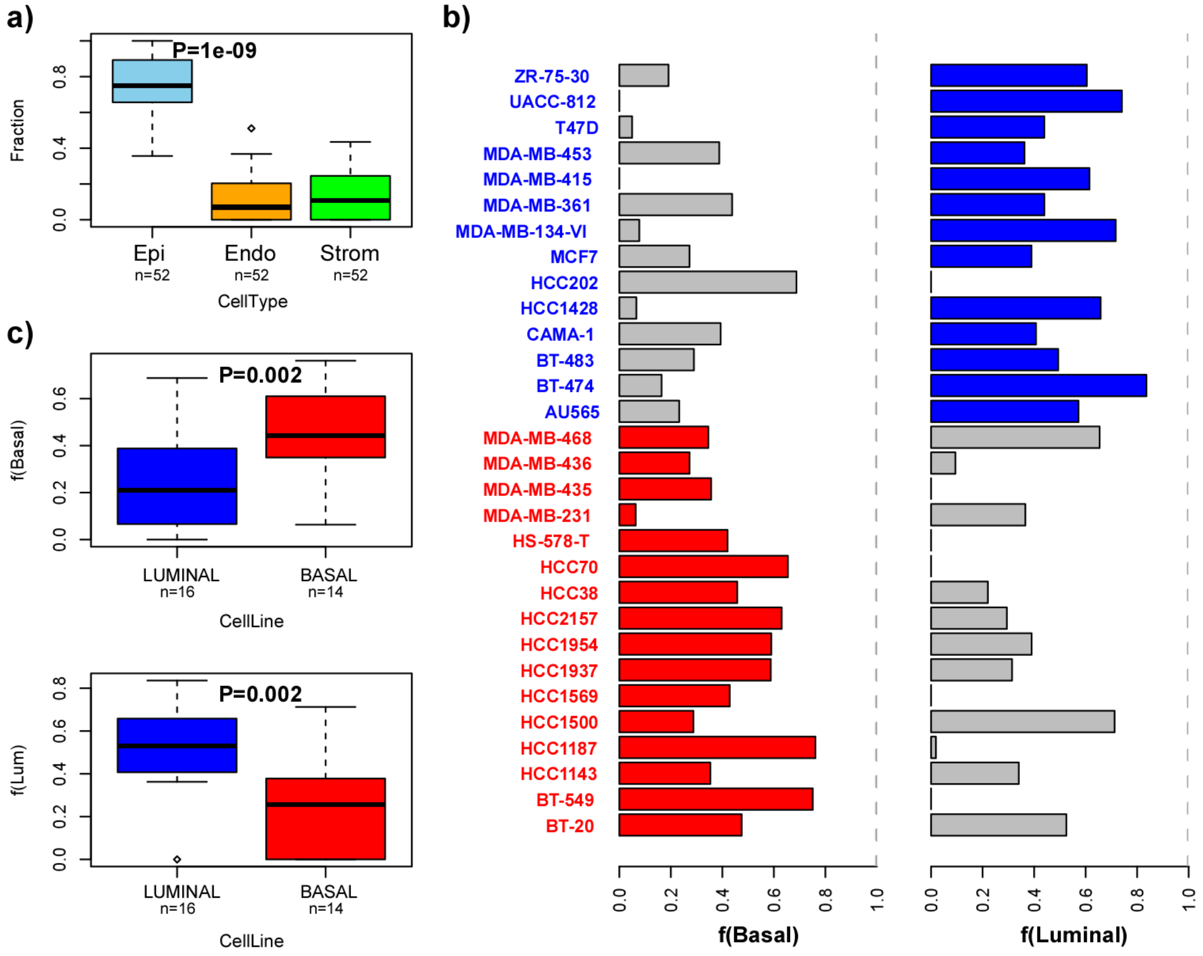

Fig.S13: Validation of EPISCORE DNAm reference (basal & luminal epithelial references) derived from MCA1 SmartSeq2 breast tissue atlas. a) Estimated total epithelial, endothelial and stromal fractions across 52 breast cancer cell-lines. P-value derives from a one-tailed paired Wilcoxon test comparing epithelial fraction to the sum of endothelial and stromal fractions. **b)** For a subset of 30 breast cancer cell-lines that have been previously classified into luminal (blue) and basal (red) subtypes, we display the estimated basal and luminal epithelial fractions. **c)** Boxplots comparing the estimated fraction of basal (top panel) and luminal (lower panel) cells across cell-lines characterized as being luminal (n=16) and basal (n=14), respectively. P-values are derived from a one-tailed Wilcoxon rank sum test.

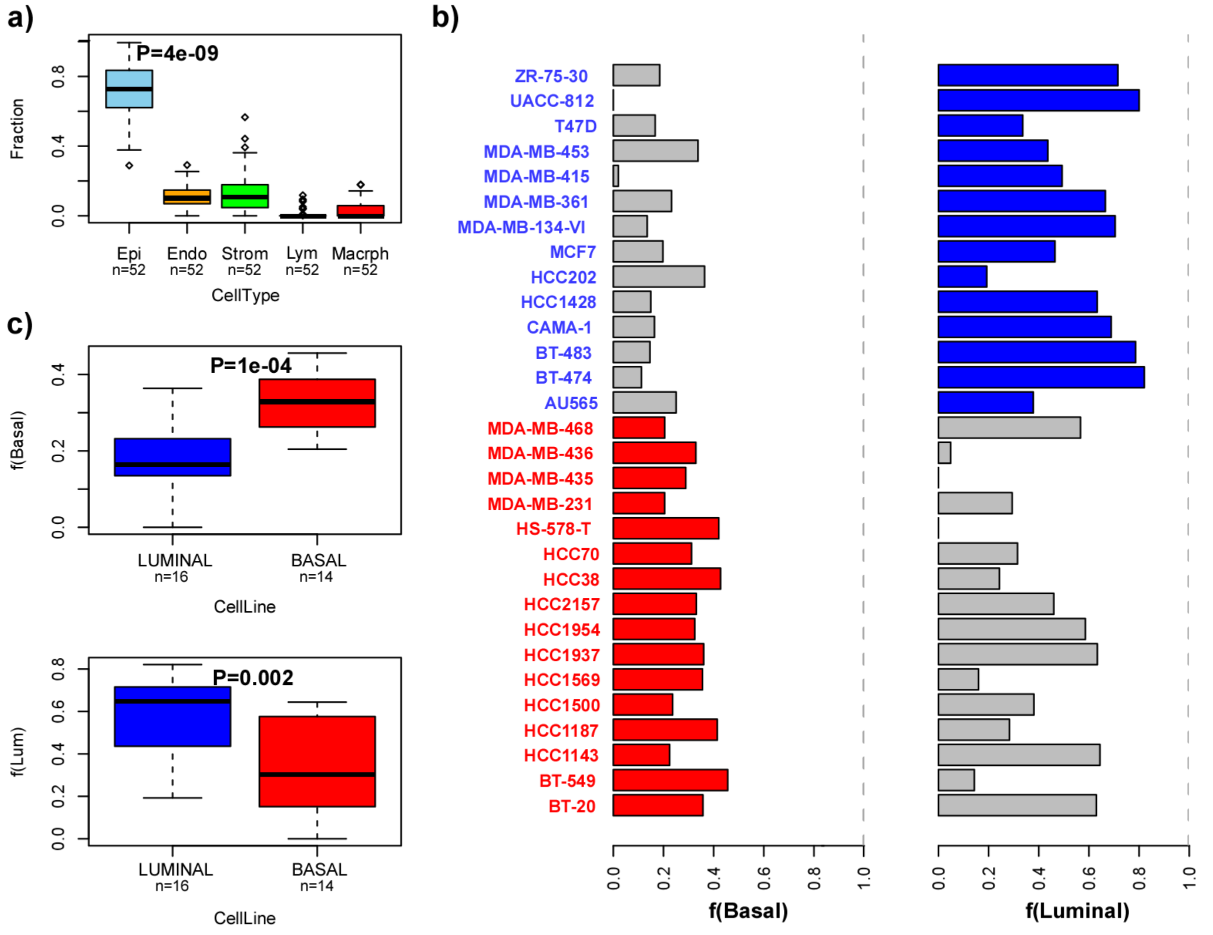

Fig.S14: Validation of EPISCORE DNAm reference (basal & luminal epithelial references) derived from MCA1 10X breast tissue atlas. a) Estimated total epithelial, endothelial and stromal fractions across 52 breast cancer cell-lines. P-value derives from a one-tailed paired Wilcoxon test comparing epithelial fraction to the sum of endothelial and stromal fractions. **b)** For a subset of 30 breast cancer cell-lines that have been previously classified into luminal (blue) and basal (red) subtypes, we display the estimated basal and luminal epithelial fractions. **c)** Boxplots comparing the estimated fraction of basal (top panel) and luminal (lower panel) cells across cell-lines characterized as being luminal (n=16) and basal (n=14), respectively. P-values are derived from a one-tailed Wilcoxon rank sum test.

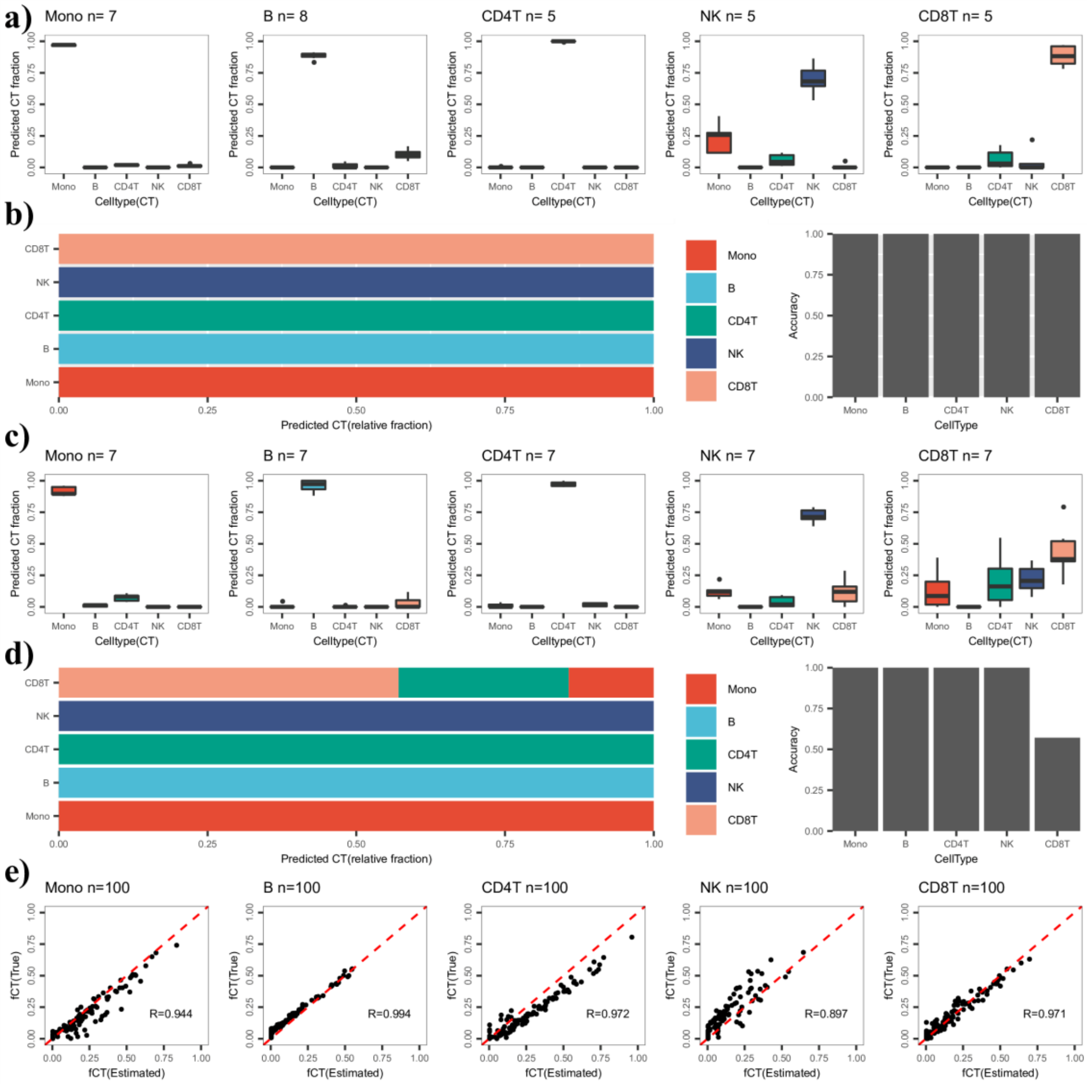

Fig.S15: Validation of Monaco's bulk RNA-seq reference matrix. a) Boxplots of estimated cell-type fractions for purified bulk RNA-seq samples from the Haemosphere-human dataset. The number of purified samples of each cell type is given. Cell-type fractions were estimated using robust partial correlations (RPC) with the expression reference defined over 5 blood subtypes (CD14+ Monocytes, CD19+ B, CD4+ T, CD56+ NK, and CD8+ T) as derived from the Monaco et al PBMC68k 10x data. **b)** Left: Barplots displaying the relative fractions of purified samples from Haemosphere-human dataset classified to each cell-type (labeled on y axis), where the classification was based on the maximum cell-type fraction in a). Right: Barplot displaying the overall accuracy to correctly classify each purified sample. **c)** The same as a) but now using the bulk expression microarray data of purified blood cell subtypes from Watkins et al. **d)** as b) but now using the bulk expression microarray data of purified blood cell subtypes from Watkins et al. **e)** Scatterplots of the true cell-type fraction (y-axis) against the estimated cell-type fraction(x-axis) for the five main PBMC cell subtypes. In each panel, there are 100 in-silico mixtures of bulk RNA-seq profiles of purified blood cell subtypes from Haemosphere-human dataset. The mixing proportions of each cell type (CD14+ Monocytes, CD19+ B, CD4+ T, CD56+ NK, and CD8+ T) were generated with a uniform Dirichlet distribution. RPCs with Monaco expression reference was used to obtain the estimated cell-type fractions.

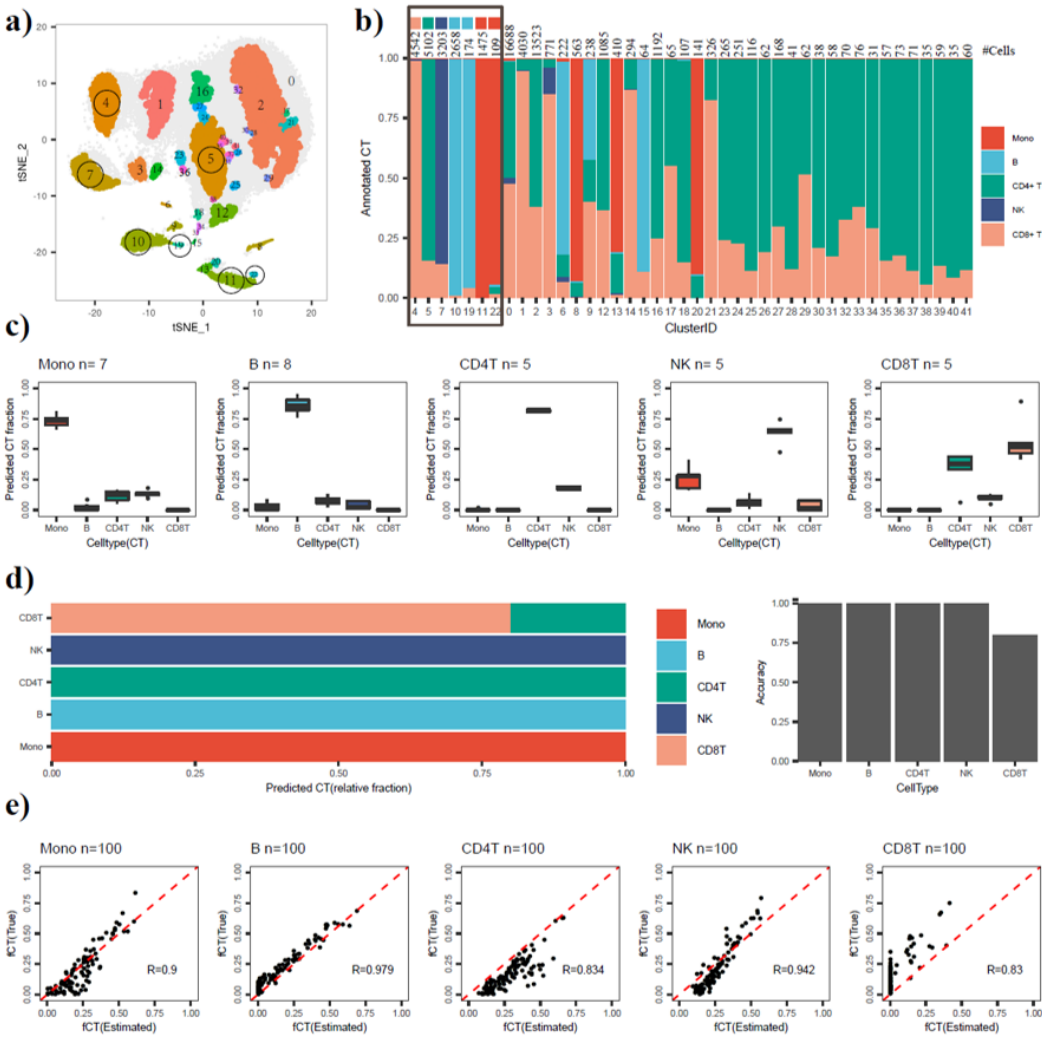

Fig.S16: Construction and validation of peripheral blood scRNA-Seq reference matrix. a) t-SNE plot of 25252 cells from PBMC68k 10x data, displaying all the main inferred clusters (labeled by clusterID). **b)** Barplot displaying for each cluster, the proportion of cells in that cluster annotated to CD14+ Monocytes, CD19+ B, CD4+ T, CD56+ NK, and CD8+ T-cells. Annotation of each cell was done by running RPC with Monaco's expression reference. The number of cells in each cluster is indicated at the top, and the dominant clusters selected for marker gene selection are enclosed in the displayed rectangle. **c)** Boxplots of estimated cell-type fractions for the purified bulk RNA-seq samples from Haemopaedia-Human. The number of purified samples of each blood cell type is given. Cell-type fractions were estimated using RPC with the scRNA-Seq expression reference as given in b). **d)** Left: Barplots displaying the relative fractions (x-axis) of purified samples of a given cell-type (labeled on y axis) that were predicted to be CD14+ Monocytes, CD19+ B, CD4+ T, CD56+ NK, and CD8+ T-cells, based on the cell-type fraction estimates obtained in c). Right: Barplot displaying the overall accuracy of the classification. **e)** Scatterplots of the true cell-type fraction (y-axis) against the estimated cell-type fraction (x-axis) for each cell-type. Fractions for each cell-type were drawn from a uniform Dirichlet distribution, and RPC+scRNA-Seq expression reference was used to obtain the estimated fraction. Each panel displays 100 in-silico mixtures of purified blood cell type samples from the Haemopaedia-Human bulk RNA-Seq dataset.

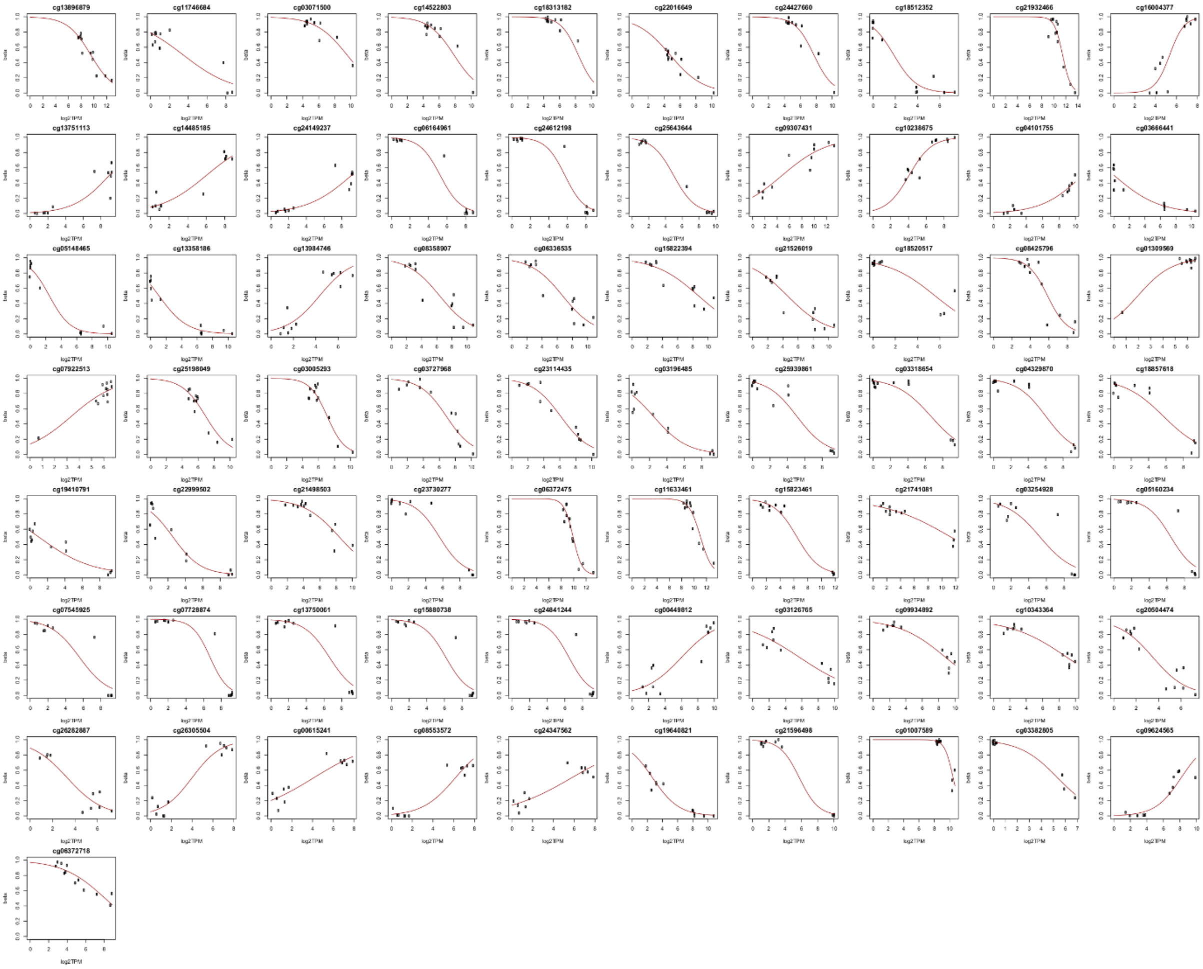

Fig.S17: Bulk DNAm and bulk RNA-Seq profiles of the selected 71 marker CpGs across purified blood cell subtype samples. Scatterplots of beta DNAm-values (y-axis) versus bulk RNA-Seq gene expression (x-axis) for the selected 71 marker CpGs, as indicated. Red curve denotes the logistic regression fit. These marker CpGs map to distal enhancer regulatory elements of marker genes in the scRNA-Seq reference matrix of PBMCs, which exhibited significant association between DNAm (as measured with WGBS data of purified blood cell subtypes from BLUEPRINT/IHEC) and gene expression (normalized bulk RNA-Seq data of purified blood cell subtypes from Monaco et al), as assessed over 12 blood cell subtypes: central memory CD8 T cells, effector memory CD8 T cells, terminal effector CD8 T cells, regulatory T cells, naïve B cells, switched memory B cells, non-switched memory B cells, classical monocytes, natural killer cells, dendritic cells, neutrophils and terminal effector CD4 T cells.

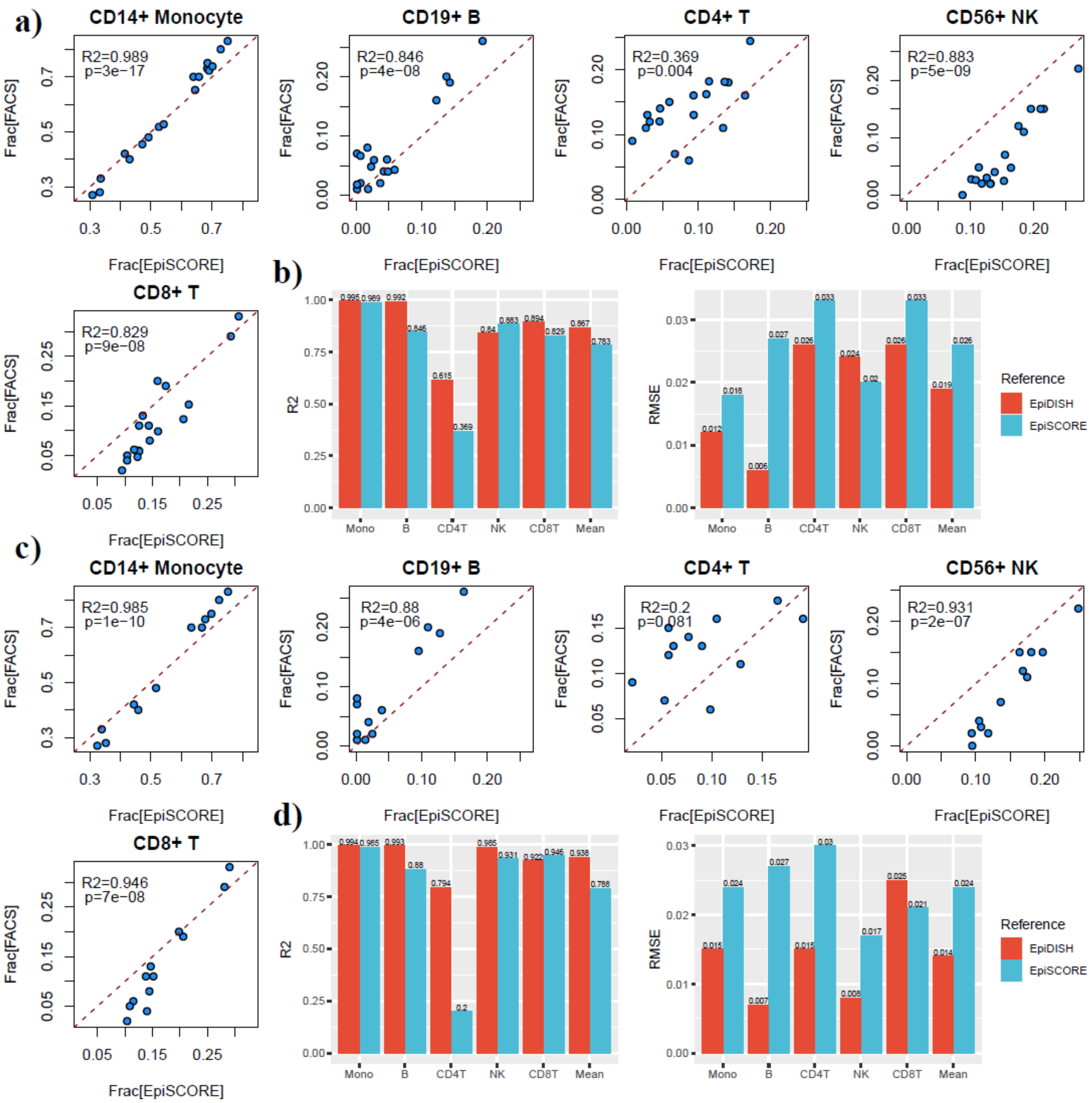

Fig.S18: Evaluation of EpiSCORE PBMC DNAm reference in IDOL datasets. **a)** Scatterplots of true fraction (FACS or experimental mixture, y-axis) vs estimated fraction from EpiSCORE (x-axis) for each of the 5 cell types present the EpiSCORE PBMC DNAm reference, as evaluated in the whole blood HM450k data from Koestler et al. Because Koestler's data is whole blood, we summed the FACS estimates for all myeloid cells together, to allow comparison with the 5 cell-type PBMC reference. Red dashed line indicates the line of unit gradient. The R-square and p-value of linear regression fit is given. **b)** Barplots of the R-square and root mean square error (RMSE) of cell type estimation with RPC with EpiDISH and EPISCORE reference. **c)** The same as a), but with Salas et al EPIC data. **d)** The same as b), but with Salas et al EPIC data.
