# Supplementary material for: EPISCORE: cell type deconvolution of bulk tissue DNA methylomes from single-cell RNA-Seq data
Source: Genome Biol. 2020 Sep 4;21:221. doi: 10.1186/s13059-020-02126-9 (PMC7650528; doi:10.1186/s13059-020-02126-9)
Supplement: Supplementary file 6 — Additional file 6. Review history. [file 13059_2020_2126_MOESM6_ESM.docx]

Review History

**First round of review**

**Reviewer 1**

**Are you able to assess all statistics in the manuscript, including the appropriateness of statistical tests used?**

Yes: the use of statistical methods is appropriate

**Comments to author:**

In their manuscript "Cell-type deconvolution of bulk tissue DNA methylomes from single-cell RNA-Seq data", Teschendorff et al. deliver present EPISCORE, a technically sound and powerful method that connects single cell RNA-seq and DNA methylation in an elegant fashion. While next-generation cell type deconvolution methods for bulk RNA-seq data generate high quality signatures from single cell RNA-seq data, this is not applicable to the deconvolution of bulk DNA methylomes, as single cell DNA methylation data is very scarce and far from the quality of single cell RNA-seq data. EPISCORE mitigates this issue by building signatures for the deconvolution of bulk DNA methylomes from single cell RNA-seq data. In a first step, cell-type-specific gene signatures are identified. These are used to impute promoter methylation where a sufficiently strong link to gene expression can be observed in matched purified bulk RNA-seq and DNA methylation data. The manuscript goes to greath lengths to validate the applied method, and shows promising results in lung, breast and blood tissue. My suggestion thus mostly focus on a clearer presentation of some methodological details and parameters. I would like to add that I could install the EPISCORE R package without issues and found it well documented with a helpful vignette. As single cell RNA-seq data becomes ubiquitous, EPISCORE is a timely and important contribution to the field.

\# Major

- In the supplemental material the authors show that more fine grained cell type resolution can be achieved in blood. Do the authors think it is feasible to discern individual leukocytes in solid tissues such as the lung tissue examined here? if not, is this a limitation of the scRNA-seq data (i.e. signature creation) or the DNAm bulk data (i.e. deconvolution)?

- I believe the manuscript should be clearer on the distinction between EPISCORE and the CellDMC algorithm. In particular in the discussion, it does not become clear that EPISCORE is used to infer cell type proportions while it is not by itself capable of identifying cell-type specific differential methylation between conditions. This is done by using CellDMC on top and should be stated clearly.

- The MSS score used for identifying cell type-specific genes is relatively stringent and the authors suggest relaxing the MSS if only few markers are found. Did the authors try higher MSS? How stable are the results or by how much do they change?

- why is the imputation step only possible for 10-20% of marker genes? Is this motivated with the overlap between step I and II? How did the authors come up with this number?

- EPISCORE considers only promoter methylation. Did the author consider enhancer elements, which might also be positively correlated and could potentially improve results? It might be worth picking this up in the discussion, as in particular the distinction of fine-grained cell types may not be feasible without considering enhancer elements.

- in step II, the manuscript states that "In the case of DNAm, we initially considered both average promoter DNAm levels". What do you mean with initially?

- in step III, the choice of 0.2 as threshold for the posterior probability was not motivated or discussed (neiher qualitatively nor quantitatively)

- in step III the authors suggest to salvage marker genes by logistic regression. While this makes sense, it did not become clear when this rule is applied in practice and how these results compared to the ones from the gamma mixture model. In other words, how much is gained by using a gamma mixture model over logistic regression?

- single cell ATAC-seq data is very popular these days and allows genome-wide profiling of DNA accessibility. While the coverage here is rather poor, I would like to see a discussion about the potential of this data type for deconvolution of DNA methyltomes, e.g. by narrowing down cell-type specific enhancers that could be considered in addition the the promoters for EPISCORE.

\# Minor

- The TCGA is doubling the article as it's part of the acronym, remove 'the'

- there is a typo in the methods: a poteriori instead of a posteriori

- abstract: "unlocks the door" - too colloquial in my opinion.

- section "The Episcore algorithm" - constructed in previous step -> missing article "the previous step"

**Reviewer 2**

**Are you able to assess all statistics in the manuscript, including the appropriateness of statistical tests used?**

Yes: I assessed the statistics in my review

**Comments to author:**

In the manuscript entitled "Cell-type deconvolution of bulk tissue DNA methylomes from single-cell RNA-seq studies", Teschendorff and colleagues describe and extensively validate a novel method for assembling DNA methylation reference libraries (for subsequent deconvolution of bulk DNA methylation profiles and cell-specific analyses of DNA methylation) by leveraging publicly available data from scRNA-seq experiments. Their approach, EPISCORE, is resourceful in that it makes use of already available data sets, biologically sensible based on the way in which cell specific DNA methylation signatures are inferred from cell specific gene expression data, and creative/clever in its formulation. I agree with the author's sentiment that EPISCORE has the potential to open new lines of inquiry and feel that the proposed method addresses what has been a bottleneck for recently proposed statistical/analytical approaches for conducting cell-specific analyses based on DNA methylation data profiled in heterogeneous tissues (aka bulk methylation data). With respect to the latter, the bottleneck of such approaches, including the CellDMC approach developed by the authors of the present manuscript, is having accurate estimates of the underlying cellular landscape for all of the samples for which bulk DNA methylation data is available. While this is generally not problematic for tissues such as whole blood or PBMCs, as reliable and validated reference libraries exist that allow for highly accurate deconvolution estimates, it is potentially problematic for tissues for which no such reference libraries exist. EPISCORE addresses this issue and has the potential to greatly expand the scope of cell-specific DNA methylation analyses that can be performed based on bulk DNA methylation data. Other highlights of this manuscript include: the use of several different tissue types (lung, breast, and whole blood) in which to validate the proposed approach, dissemination of R code for EPISCORE to the Comprehensive R Archive Network, and clarity of the presentation. With that being said, my comments/suggestions are mostly minor and are intended to improve the quality and transparency of the manuscript.

1. While I commend the authors for the thoroughness of their validation efforts, I am a little perplexed why the authors chose to use in-silico mixtures to validate the cell deconvolution estimates obtained from EPISCORE when applied to whole blood or PBMCS (Supplementary Note)? I understand that the use of in-silico mixtures are likely necessary for lung and breast, as to my knowledge there is no publicly available data sets that consist of DNA methylation profiles of those tissue types accompanied by laser capture microdissection derived estimates of the underlying cellular landscape that would allow for gold-standard validation of the cell estimates obtained via application of EPISCORE, but there do exist such data sets for whole blood (see PMID: 29843789 and PMID: 26956433). I am fairly confident that EPISCORE will still return accurate cell deconvolution estimates when applied to such data sets, however it would be interesting to see how the accuracy of the EPISCORE-derived estimates (e.g., R2 and RMSE) benchmarks as compared to those obtained when an established/validated reference library for whole blood deconvolution is used? My expectation is that the EPISCORE-derived estimates will be somewhat less accurate, but how much so? It would be useful to know how the two compare as the established/validated reference library can be viewed somewhat as establishing a "ceiling" for the accuracy of reference-based deconvolution. Another reason for validating EPISCORE-derived estimates of cell composition using whole-blood DNA methylation data sets with accompanying FACS measurements or "known" reconstruction proportions, is that it allows one to see whether EPISCORE-derived deconvolution accuracy is invariant to the underlying proportion of the mixture the cell makes up; in the present manuscript, the in-silico mixture proportions are being simulated from a Dirichlet distribution whose K-dimensional parameter vector consists of equal elements, leading to a relatively "uniform landscape" of proportions across the K cell types. One final reason for the above suggestion is that the creation of the in-silico mixtures assumes the same data generating model as is used in RPC for deconvolution. Because of this, the accuracy of deconvolution estimates of in-silico mixtures may be a little more optimistic than what you get from deconvoluting samples with both whole-blood methylation data and accompanying FACS measurements.

2. In general, I found the description of step (iii) of EPISCORE (pages 22-23) a little difficult to follow and think a schematic diagram or some sort of conceptional figure might allay my confusion. While the author's claim that they are using a probabilistic Bayesian imputation model, I do not recall seeing any mention of what the assumed prior distributions are? The formula given on page 22 is also somewhat confusing and possibly contributing to my misunderstanding of what is actually being done here. I think this formula is simply a statement that the posterior distribution that gene "g" in sample "s" is expressed, is proportional to the likelihood times the prior, except that the "~" ("distributed as" symbol is being used instead of the symbol for "proportional to"). The other thing that is throwing me off in this formula is the use of the capital Greek symbol for gamma to represent the likelihood for expression data for gene "g" in sample "s". My recommendation would be to just use f(.) and simply state that f(.) is assumed to follow a Gamma distribution. I think what I was expecting to see in this section is that the imputation model would be some sort of beta-regression model, modeling DNA methylation beta-values (or average/median thereof) as the dependent variable, against expression as the independent variable. Under a Bayesian framework, one could assume weakly informative priors for the mean and dispersion parameter and use MCMC or Gibbs sampling to draw from the posterior distribution. This model could then be applied to the gene expression marker matrix in step 1 to obtain estimates of DNAm (e.g., mean of the posterior predictive distribution) to be used in deconvolution.

**Authors Response**

**Point-by-point responses to the reviewers’ comments:**

Reviewer #1:

General Comment: In their manuscript "Cell-type deconvolution of bulk tissue DNA methylomes from single-cell RNA-Seq data", Teschendorff et al. deliver present EPISCORE, a technically sound and powerful method that connects single cell RNA-seq and DNA methylation in an elegant fashion. While next-generation cell type deconvolution methods for bulk RNA-seq data generate high quality signatures from single cell RNA-seq data, this is not applicable to the deconvolution of bulk DNA methylomes, as single cell DNA methylation data is very scarce and far from the quality of single cell RNA-seq data. EPISCORE mitigates this issue by building signatures for the deconvolution of bulk DNA methylomes from single cell RNA-seq data. In a first step, cell-type-specific gene signatures are identified. These are used to impute promoter methylation where a sufficiently strong link to gene expression can be observed in matched purified bulk RNA-seq and DNA methylation data. The manuscript goes to greath lengths to validate the applied method, and shows promising results in lung, breast and blood tissue. My suggestion thus mostly focus on a clearer presentation of some methodological details and parameters. I would like to add that I could install the EPISCORE R package without issues and found it well documented with a helpful vignette. As single cell RNA-seq data becomes ubiquitous, EPISCORE is a timely and important contribution to the field.

*Response: We would like to thank the reviewer for taking time to evaluate our manuscript. We are delighted for the very positive feedback and that the reviewer is enthused by this manuscript.*

Major points:

Comment: In the supplemental material the authors show that more fine grained cell type resolution can be achieved in blood. Do the authors think it is feasible to discern individual leukocytes in solid tissues such as the lung tissue examined here? if not, is this a limitation of the scRNA-seq data (i.e. signature creation) or the DNAm bulk data (i.e. deconvolution)?

*Response: The reviewer has raised an excellent point. The scRNA-Seq data is not a limitation as long as all the cell-types within the tissue have been captured and profiled in reasonable numbers. This however is often not the case: in the case of breast tissue, both 10X and SmartSeq2 mouse cell atlases did not capture adipocytes, a major component of breast tissue. In the case of the lung SmartSeq-2/10X mouse cell atlases, relatively few myeloid cells (<85) were captured or identified as such. Ideally, one would have profiled hundreds of cells of each cell-type in order to identify more marker genes. So, although in theory the scRNA-Seq atlas should not be a limitation, in practice, current scRNA-Seq assays do present limitations, and indeed we already mentioned this in Discussion. As far as the deconvolution of bulk DNAm samples is concerned, this is not a*

*limitation per se if the corresponding DNAm reference matrix being used for deconvolution contains sufficient markers (about 50 or more for each cell-type) and is sufficiently accurate. Indeed, the case of whole blood illustrates this point, where it has been possible to successfully estimate cell-type fractions for approximately 7 cell-types.*

*As to the question whether it is possible to estimate proportions of different leukocyte subtypes in lung tissue, there are first of all a number of reasons why we did not pursue this task: First, for the downstream application that we had in mind where we identify DMCTs in the main cell-type compartments (epithelial, endothelial, fibroblast and immune-cell) of lung tissue, the higher resolution of IC cell subtypes is not necessary. A second related reason is that running CellDMC with larger numbers of cell-types would correspondingly also require substantially larger numbers of samples. Indeed, we estimate that CellDMC may require thousands of bulk DNAm samples to reliably infer cell-type specific differential methylation signal at the resolution of different leukocytes within a solid tissue. A third reason is that validating predictions of different leukocyte subset fractions in solid tissues is challenging, as we are not aware of publicly available cell-count data at this resolution in solid tissues. Nevertheless, in response to the reviewer’s point we have now made an attempt to increase the cellular resolution in lung-tissue, considering three immune cell subtypes (monocytes, lymphocytes, myeloid cells), in addition to epithelial, endothelial and fibroblast cells (displayed in new SuppFig.S1 and SuppFig.S8). First, we note that the scRNA-Seq reference itself validates reasonably well at this higher cellular resolution (see SuppFig.S1, which for convenience we display below) :*

*SI fig.S1: Validation of lung-tissue scRNA-Seq reference matrix at resolution of 6 cell-types. a) Boxplots of estimated cell-type fractions for single-cells from the lung MCA1 10X atlas, which were annotated as epithelial (Epi), endothelial (Endo), fibroblast (Fib), lymphocyte (B-cell, T-cell or NK-cell), monocyte (Mono) or myeloid (Mye). The number of single cells annotated to each cell-type is given. Cell-type fractions were estimated using RPC with an expression reference defined over 6 cell-types (epithelial, endothelial, fibroblast, lymphocyte, monocyte and myeloid) using the SmartSeq2 scRNA-Seq data from MCA1. b) Left: Barplots depicting the relative fractions of single-cells annotated to each type (labeled on y-axis) which were predicted to be epithelial, endothelial, fibroblast, lymphocyte, monocyte or myeloid cell (labeled by different colors) based on the cell-type fraction estimates obtained in a). Right: barplot displaying the overall accuracy of using RPC+expression reference to correctly classify each cell type.*

*As we can see from this figure, the classification accuracy of all cell-types remains over 98% except for the myeloid cells, possibly due to their lower numbers in the SmartSeq2 MCA1 set, where the reference was derived from. We note that in the 10X lung MCA1 set too, myeloid cells were far fewer (87 vs. 1275 lymphocytes and 381 monocytes). Second, we have also assessed the performance of the DNAm cell-type deconvolution at this higher resolution of 6 cell-types, the result of which is displayed in new SuppFig.S8 (for convenience we display this below):*

*SI fig.S8: Performance of EpiSCORE lung DNAm reference as a function of cellular resolution. Violin plots of the Pearson Correlation Coefficient (PCC, y-axis) vs. cell-type (x-axis), where the PCC is estimated by computing the correlation between the estimated cell-type fraction and the true fraction over 100 in-silico mixtures. Each violin plot contains 100 datapoints corresponding to 100 Monte-Carlo runs. Left panel depicts the case where the EpiSCORE DNAm reference for lung tissue is constructed over 4 main cell-types (Epithelial, Endothelial, Fibroblast and Immune-Cell), whereas right panel depicts the case where the EpiSCORE DNAm reference is constructed over 6 cell-types (Epithelial, Endothelial, Fibroblast, Lymphocyte, Monocyte and Myeloid).*

*We can see from this figure that, although the average correlative accuracy over epithelial, endothelial and fibroblast cells is similar (it drops for epithelial and fibroblasts but increases for endothelial cells), performance does drop very significantly when considering three different immune cell subtypes. To improve the discrimination of the different immune cell subtypes at the DNAm level may require introduction of enhancer elements, as we have done in the case of PBMCs. We have explored combining promoter and enhancer elements together, but feel that this would indeed justify a separate manuscript, as with the current work we want to demonstrate proof of concept that scRNA-Seq reference matrices can be used to impute approximate DNAm reference matrices, albeit at a coarser cellular resolution.*

*We should also add that the question posed by the reviewer is one that we have also explored previously in the context of the HEpiDISH algorithm (see Zheng SC et al Epigenomics 2018), where DNAm references are built from existing DNAm profiles from epithelial and fibroblast cell-lines, and purified blood cell-type samples. HEpiDISH works by inferring cell-type fractions in a hierarchical fashion, e.g. inferring first a total immune-cell, total epithelial and total fibroblast fraction and subsequently inferring fractions of individual leukocyte subtypes using a separate second DNAm reference matrix specific to immune cells. The main limitation encountered in this previous study was the lack of DNAm data representing purified epithelial, fibroblast and endothelial cells which are present in each solid epithelial tissue, and which is necessary to build corresponding tissue-specific DNAm references. This is indeed why we now introduce the EpiSCORE framework to build more complete DNAm reference matrices for solid tissue types. Once these have been built, one could in principle now apply the HEpiDISH framework combining these EpiSCORE-derived DNAm reference matrices with a separate DNAm reference matrix that is specific to say immune cell subtypes. There are however a number of technical limitations that prevent us from implementing this strategy in the current manuscript. Once again, we hope to address this limitation in future work, where combining EpiSCORE with HEpiDISH may well be an alternative strategy to obtain cell-type proportions for all leukocyte subsets within solid tissues.*

Comment: I believe the manuscript should be clearer on the distinction between EPISCORE and the CellDMC algorithm. In particular in the discussion, it does not become clear that EPISCORE is used to infer cell type proportions while it is not by itself capable of identifying cell-type specific differential methylation between conditions. This is done by using CellDMC on top and should be stated clearly.

*Response: We thank the reviewer for raising this point. We fully agree, and we now make it clearer in Discussion, as requested.*

Comment: The MSS score used for identifying cell type-specific genes is relatively stringent and the authors suggest relaxing the MSS if only few markers are found. Did the authors try higher MSS? stable are the results or by how much do they change?

*Response: We thank the reviewer for raising this important point, and indeed we apologize for not clarifying what the choice of MSS thresholds was in each application. In the case of lung-tissue we constructed the scRNA-Seq and DNAm references at the resolution of 4 cell-types (epithelial, endothelial, stromal and immune-cell) using the maximal possible MSS threshold (this value equals*

*3) for each of the 4 cell-types. Here, the maximal value is 3 because there is maximally only 3 other cell-types where a given marker gene is not expressed. In the case of breast tissue we constructed the scRNA-Seq and DNAm references at the resolution of 6 cell-types (basal, luminal, endothelial, stromal, lymphocytes, macrophages) and corresponding MSS thresholds for these cell-types were 5, 5, 4, 4, 3, 3, respectively. These choices were motivated so as to ensure sufficient and approximately equal numbers of markers for each cell-type. We have now clarified this in Methods.*

*In order to address the reviewer’s question about the robustness of the results to the choice of MSS thresholds, we have now performed additional analyses in both lung and breast tissue, rederiving new scRNA-Seq reference matrices at different values of MSS, and subsequently validating these in the independent scRNA-Seq dataset from the Mouse Cell Atlas. Results indicate that classification accuracy remains high. We have also compared EpiSCORE’s performance in predicting true cell-type fractions from in-silico mixtures of DNAm profiles, for different combinations of MSS thresholds, finding that results are also very robust. Below we display the new SuppFig.S7 that demonstrates these results.*

*SI fig.S7: Robustness of results to choice of MSS-thresholds. a) Classification accuracy (y-axis) of the scRNA-Seq reference matrix derived from the SmartSeq2 (SS2) lung Mouse Cell Atlas dataset in the corresponding scRNA-Seq 10X lung set, as a function of the marker-specificity score (MSS) threshold. MSS=m (m=0,1,2,3) means that marker genes were required to not only be more highly expressed in one cell-type compared to all others, but that the given gene is also not expressed in at least m of the other cell-types. e.g. MSS=3 means that the marker genes are not expressed in all 3 of the other cell-types, whereas MSS=1 means that a marker gene is allowed to be expressed in 2 of the other 3 cell-types. We use the same MSS-threshold for each cell-type. b) Violin plots displaying the Pearson Correlation Coefficient (PCC) between the true cell-type fractions and the estimated ones from the EpiSCORE DNAm reference matrix, as derived across 100 in-silico mixtures, and for a total of 100 Monte-Carlo simulations. Here MSS1 means MSS=3 for all 4 cell-types, whereas MSS2 means MSS=2 for all 4 cell-types. We provide unpaired (U) and paired (P) two-tailed Wilcoxon rank sum test P-values, as estimated over the 100 Monte-Carlo runs. c) As a), but for the case of the SmartSeq2 and 10X breast mouse cell atlas datasets. d) As b), but in the case of breast, the scRNA-Seq reference matix was constructed using MSS1=(5,5,4,4,3,3) for luminal, basal, endothelial, fibroblast, lymphocytes and macrophages, respectively, and MSS2=5 for all 6 cell-types. We note that in the validation of the DNAm reference matrix, there are no sufficient normal luminal and basal DNAm profiles in the public domain, which is why validation of the in-silico mixtures is carried out at the level of total epithelial cells, i.e. estimated basal and luminal fractions were summed and compared to the true epithelial fraction in the simulation.*

Comment: why is the imputation step only possible for 10-20% of marker genes? Is this motivated with the overlap between step I and II? How did the authors come up with this number?

*Response: We thank the reviewer for raising this important point, and apologize if this was unclear. As we stated in Methods section “For the sequencing-based set, where we started out from 18651 genes, we obtained 2174 anti-correlated genes (for promoters), whereas for the SCM2 set we performed the analysis for 13290 genes, finding 1152 anti-correlated genes (for promoters).”. Thus, for the RMAP set, the fraction of imputable genes is approximately 12%, whereas it is 9% for the SCM2 dataset. This sets the lower bound at around 10%. In practice however, when we consider genes making up a scRNA-Seq reference expression matrix, the marker genes in these reference matrices are naturally enriched for imputable genes, so the fraction of marker genes which can be imputed is higher, and based on the lung & breast tissue sets we have considered in our work, the fraction of imputable marker genes is higher at around 20%. In response to the reviewer’s point, we have now clarified this in Methods.*

Comment: EPISCORE considers only promoter methylation. Did the author consider enhancer elements, which might also be positively correlated and could potentially improve results? It might be worth picking this up in the discussion, as in particular the distinction of fine-grained cell types may not be feasible without considering enhancer elements.

*Response: We thank the reviewer for raising this excellent point. We have indeed explored using distal regulatory elements, which includes enhancers. As far as the solid tissues is concerned and at the cellular resolution considered here, inclusion of enhancer elements did not lead to improved performance. As the reviewer correctly points out, taking distal regulatory/enhancer elements into account may well be necessary if higher cellular resolution is required, for instance, if we wish to estimate cellular proportions of distinct lymphoid subsets within a tissue like lung or breast. Supporting this, we found that using distal regulatory elements was necessary when applying EpiSCORE to peripheral blood mononuclear cells (PBMCs), where we need to discriminate many lymphoid subsets from each other. Our impression however is that we still need to await for improvements in mapping enhancer-gene networks, before we can take EpiSCORE to the next level. We hope to accomplish this in future work. In response to the reviewer’s point, and as requested, we have now added some sentences to Discussion where we elaborate on these points. We have also incorporated the PBMC analysis into a separate subsection within the main manuscript, which should therefore emphasize more the fact that we did consider enhancer elements in this particular application.*

Comment: in step II, the manuscript states that "In the case of DNAm, we initially considered both average promoter DNAm levels". What do you mean with initially?

*Response: We thank the reviewer for asking this. We agree that we did not express this well. When developing EpiSCORE, we wanted to assess if focusing solely on promoter DNAm levels would be sufficient. Indeed, distal regulatory enhancer elements are also known to be important cell-type specific factors, and so it is natural, when developing EpiSCORE, that we initially considered promoter DNAm and distal probes separately. We found that in tissues like lung or breast (and at the cellular resolution considered), promoter DNAm patterns were informative. In peripheral blood mononuclear cells (PBMCs) however, where there is a need to distinguish more similar cell-types, we used an approach based on distal probes, as this worked better. However, let us stress that EpiSCORE is not really designed for blood or PBMCs, as for these tissues it is more feasible to build DNAm references through FACS sorting. On the other hand, if one seeks to build a DNAm reference for a tissue like whole blood at a much higher resolution of say 10 or more cell-types, then using distal probes within the EpiSCORE framework could turn out to be a way to build such a high resolution DNAm reference matrix, assuming of course that a corresponding high resolution scRNA-Seq whole blood atlas exists. This is certainly an avenue for further exploration, but the focus of the current manuscript is on solid epithelial tissues, and at a coarser resolution of 4-6 cell-types, and for this task our manuscript demonstrates that focusing on promoter DNAm patterns is adequate. In response to the reviewer’s point, we have now rephrased some of the relevant sentences in Methods, so as to avoid confusion, and have also added a full subsection in Methods describing the application of EpiSCORE to PBMCs, and which is based on considering distal regulatory elements.*

Comment: in step III, the choice of 0.2 as threshold for the posterior probability was not motivated or discussed (neiher qualitatively nor quantitatively)

*Response: The reviewer is right and we apologize for forgetting to justify this threshold. First, we should point out that once the expression values (see panel-a) below) are converted to probabilities of expression (see panel-b) below), that the distribution of these probabilities becomes strongly bi-modal, i.e. there are relatively few data points where probabilities are in the middle range (see panel-c) below). Therefore, results are very stable if we were to relax the threshold to say p(E|X) < 0.3 (see panel-d) below).*

*On the other hand, we may also wish to impose a more stringent threshold, yet doing so jeopardizes that for specific genes we no longer have samples where the genes are not expressed, and hence for these genes we would then need to use logistic regressions in order to do the imputation. So, the threshold p(E|X) < 0.2 is sensible as it ensures that for many genes we can do the imputation from the gamma-mixture model, whilst also ensuring that on average 80% of the samples where we declare the gene not to be expressed, are correct calls. In response to the reviewer’s point, we now further clarify this in Methods, and have added the figure above as a new SuppFig.S5.*

Comment: in step III the authors suggest to salvage marker genes by logistic regression. While this makes sense, it did not become clear when this rule is applied in practice and how these results compared to the ones from the gamma mixture model. In other words, how much is gained by using a gamma mixture model over logistic regression?

*Response: We thank the reviewer for raising this question. We implement the logistic regression whenever there are no samples in the SCM2/RMAP databases where the given marker gene is not expressed. This is because imputation via the gamma-mixture model requires samples where the marker gene is not expressed, and so for marker genes where this requirement is not satisfied we resort to fitting a logistic regression between DNAm and gene-expression, and then extrapolating to the state of zero-expression to obtain the imputed DNAm level. All of this was stated in Methods, but we have now added a brief additional clarification. In response to the reviewer’s point and also to a related point raised by the other reviewer, we have also added a new SuppFig.S4 which explains the imputation procedure based on the gamma-mixture model fit.*

*In practice, we have found that the gamma-mixture model based imputation works better, because it is far less sensitive to the precise functional relationship between DNAm and gene expression (it only assumes an overall inverse association). The inclusion of the logistic regression step to salvage more marker genes is only included to increase marker gene number in situations where too few marker genes are imputable via the gamma-mixture model procedure.*

Comment: single cell ATAC-seq data is very popular these days and allows genome-wide profiling of DNA accessibility. While the coverage here is rather poor, I would like to see a discussion about the potential of this data type for deconvolution of DNA methyltomes, e.g. by narrowing down cell-type specific enhancers that could be considered in addition the the promoters for EPISCORE.

*Response: The reviewer has raised another excellent point. We agree that scATAC-Seq data could prove valuable to hone in on cell-type specific enhancers and that such information could be integrated into EpiSCORE. As requested, we now briefly mention this possibility in the Discussion section.*

Minor points:

Comment: The TCGA is doubling the article as it's part of the acronym, remove 'the'

*Response: We have corrected this.*

Comment: there is a typo in the methods: a poteriori instead of a posteriori

*Response: We have corrected this.*

Comment: abstract: "unlocks the door" - too colloquial in my opinion.

*Response: As requested, we have removed this expression from the abstract. It was indeed not*

*necessary.*

Comment: section "The Episcore algorithm" - constructed in previous step -> missing article "the

previous step"

*Response: We have clarified this by now stating “...in step (iii)”.*

Reviewer #2:

General Comment: In the manuscript entitled "Cell-type deconvolution of bulk tissue DNA methylomes from single-cell RNA-seq studies", Teschendorff and colleagues describe and extensively validate a novel method for assembling DNA methylation reference libraries (for subsequent deconvolution of bulk DNA methylation profiles and cell-specific analyses of DNA methylation) by leveraging publicly available data from scRNA-seq experiments. Their approach, EPISCORE, is resourceful in that it makes use of already available data sets, biologically sensible based on the way in which cell specific DNA methylation signatures are inferred from cell specific gene expression data, and creative/clever in its formulation. I agree with the author's sentiment that EPISCORE has the potential to open new lines of inquiry and feel that the proposed method addresses what has been a bottleneck for recently proposed statistical/analytical approaches for conducting cell-specific analyses based on DNA methylation data profiled in heterogeneous tissues (aka bulk methylation data). With respect to the latter, the bottleneck of such approaches, including the CellDMC approach developed by the authors of the present manuscript, is having accurate estimates of the underlying cellular landscape for all of the samples for which bulk DNA methylation data is available. While this is generally not problematic for tissues such as whole blood or PBMCs, as reliable and validated reference libraries exist that allow for highly accurate deconvolution estimates, it is potentially problematic for tissues for which no such reference libraries exist. EPISCORE addresses this issue and has the potential to greatly expand the scope of cell-specific DNA methylation analyses that can be performed based on bulk DNA methylation data. Other highlights of this manuscript include: the use of several different tissue types (lung, breast, and whole blood) in which to validate the proposed approach, dissemination of R code for EPISCORE to the Comprehensive R Archive Network, and clarity of the presentation. With that being said, my comments/suggestions are mostly minor and are intended to improve the quality and transparency of the manuscript.

*Response: We would like to thank the reviewer for taking time to evaluate our manuscript and for the very positive feedback. We are indeed delighted that the reviewer fully agrees with all the key points.*

Minor comments:

Comment1a: While I commend the authors for the thoroughness of their validation efforts, I am a little perplexed why the authors chose to use in-silico mixtures to validate the cell deconvolution estimates obtained from EPISCORE when applied to whole blood or PBMCS (Supplementary Note)? I understand that the use of in-silico mixtures are likely necessary for lung and breast, as to my knowledge there is no publicly available data sets that consist of DNA methylation profiles of those tissue types accompanied by laser capture microdissection derived estimates of the underlying cellular landscape that would allow for gold-standard validation of the cell estimates obtained via application of EPISCORE, but there do exist such data sets for whole blood (see PMID: 29843789 and PMID: 26956433).

*Response: We thank the reviewer for raising this good point. There is a very good reason why we did not evaluate the EpiSCORE PBMC DNAm reference in the IDOL datasets, and we sincerely apologize for not making that clear. This is our fault, because we had repeatedly referred to the application of EpiSCORE as if it were in whole blood, but in fact, it is in peripheral blood mononuclear cells (PBMCs), which is devoid of neutrophils and eosinophils. Indeed, we note that popular single-cell RNA-Seq technologies like 10X are not suitable for profiling neutrophils as these cells seem to have a lower RNA content, and therefore many of the scRNA-Seq studies have not been able to capture neutrophils in their assays. Hence, the scRNA-Seq atlas we constructed is for PBMCs, not whole blood. If we were to now evaluate our EpiSCORE PBMC reference in the whole blood samples or reconstructed experimental mixtures (which contain neutrophils) of the IDOL papers, we would encounter the problem that we don’t have the component representing neutrophils, and so performance could be severely compromised, since neutrophils often make up over 50% of the samples. Nevertheless, in response to the reviewer’s point, we have now evaluated the EpiSCORE derived PBMC reference in the IDOL datasets, result of which is displayed below and which has been incorporated as a new SuppFig.S18:*

*SI fig.S18: a) Scatterplots of true fraction (FACS or experimental mixture, y-axis) vs estimated fraction from EpiSCORE (x-axis) for each of the 5 cell types present the EpiSCORE PBMC DNAm reference, as evaluated in the whole blood HM450k data from Koestler et al. Because Koestler’s data is whole blood, we summed the FACS estimates for all myeloid cells together, to allow comparison with the 5 cell-type PBMC reference. Red dashed line indicates the line of unit gradient. The R-square and p-value of linear regression fit is given. b) Barplots of the R-square and root mean square error (RMSE) of cell type estimation with RPC with EpiDISH and EPISCORE reference. c) The same as a), but with Salas et al EPIC data. d) The same as b), but with Salas et al EPIC data.*

*We can see from panels a) & c) that, despite the significant limitations of (i) applying a DNAm reference without neutrophils to whole blood data, which is predominantly made up of neutrophils, and (ii) the fact that monocytes are an imperfect surrogate for neutrophils, EpiSCORE still yields reasonable R2 values for most cell-types, and significant P-values for all cell-types. Moreover, in panels-b) & d) above, we compare the mean-performance (averaged over all cell-types) of the EpiSCORE PBMC DNAm reference to that of our EpiDISH algorithm (which is comparable to say Houseman’s method using a whole blood DNAm reference containing all cell-types), and we can see that EpiSCORE only performs marginally worse. In response to the reviewer’s point, we have included the above figure and analysis on the whole blood IDOL datasets into the manuscript.*

Comment1b: I am fairly confident that EPISCORE will still return accurate cell deconvolution estimates when applied to such data sets, however it would be interesting to see how the accuracy of the EPISCORE-derived estimates (e.g., R2 and RMSE) benchmarks as compared to those obtained when an established/validated reference library for whole blood deconvolution is used? My expectation is that the EPISCORE-derived estimates will be somewhat less accurate, but how much so? It would be useful to know how the two compare as the established/validated reference library can be viewed somewhat as establishing a "ceiling" for the accuracy of reference-based deconvolution.

*Response: We agree, but of course the big caveat here (as elaborated in our response to the point above) is that we have built a DNAm reference for PBMCs, not whole blood. Once again, we apologize for our sloppiness in using the term “blood” instead of PBMCs, which we think have misled this reviewer into thinking that we derived a DNAm reference for whole blood. In response to the reviewer’s point, we have now corrected this, and make it clear that we derived a DNAm reference for PBMCs, not whole blood. As elaborated in our response to the previous point, we have also now included a new SuppFIg.S18 into SI, where we display the R2 and RMSE values on the IDOL whole blood datasets, which fully confirm the reviewer’s expectation.*

Comment1c: Another reason for validating EPISCORE-derived estimates of cell composition using whole-blood DNA methylation data sets with accompanying FACS measurements or "known" reconstruction proportions, is that it allows one to see whether EPISCORE-derived deconvolution accuracy is invariant to the underlying proportion of the mixture the cell makes up; in the present manuscript, the in-silico mixture proportions are being simulated from a Dirichlet distribution whose K-dimensional parameter vector consists of equal elements, leading to a relatively "uniform landscape" of proportions across the K cell types. One final reason for the above suggestion is that the creation of the in-silico mixtures assumes the same data generating model as is used in RPC for deconvolution. Because of this, the accuracy of deconvolution estimates of in-silico mixtures may be a little more optimistic than what you get from deconvoluting samples with both whole-blood methylation data and accompanying FACS measurements.

*Response: As elaborated in our response to the earlier points, unfortunately, we are not aware of experimental mixtures of PBMCs or of PBMC samples with matched FACS counts, that would allow us to do this type of analysis. Nevertheless, what we can do is generate in-silico mixtures of PBMCs using more realistic cell-type fractions, in line with their observed proportions and variance. We have now done this and have added the result to a new panel 1f in a new Fig.6. For convenience, we display the new Fig.6 below:*

*Panel 1e is for the case of a uniform Dirichlet distribution, whereas 1f is for the case of a Dirichlet with realistic parameter choices, and there is generally not a major difference in performance.*

Comment 2a. In general, I found the description of step (iii) of EPISCORE (pages 22-23) a little difficult to follow and think a schematic diagram or some sort of conceptional figure might allay my confusion. While the author's claim that they are using a probabilistic Bayesian imputation model, I do not recall seeing any mention of what the assumed prior distributions are? The formula given on page 22 is also somewhat confusing and possibly contributing to my misunderstanding of what is actually being done here. I think this formula is simply a statement that the posterior distribution that gene "g" in sample "s" is expressed, is proportional to the likelihood times the prior, except that the "~" ("distributed as" symbol is being used instead of the symbol for "proportional to"). The other thing that is throwing me off in this formula is the use of the capital Greek symbol for gamma to represent the likelihood for expression data for gene "g" in sample "s". My recommendation would be to just use f(.) and simply state that f(.) is assumed to follow a Gamma distribution.

*Response: We thank the reviewer for raising this point, and apologize for any confusion caused. What we are doing here is fitting a mixture of Gamma distributions to the expression values of each of the SCM2 and RMAP expression data matrices, in order to learn the parameters defining the expressed and unexpressed states. With the estimated parameters, we can then infer the posterior probability of a gene being in the expressed state given its expression value in a given sample. This invokes Bayes theorem. So, we are using the term “Bayesian” in this sense. In relation to the formula, given the confusion it has caused, we have now replaced the gamma-symbol with p , and the proportionality symbol with an equation, which hopefully makes it clearer that we are dealing with Bayes theorem.*

*The rationale for the whole procedure is that we need to know in which samples of the SCM2 and RMAP expression databases a given gene is not expressed, as the scRNA-Seq reference matrix is constructed from corresponding genes that are either expressed, or not expressed, in given cell-types. This then allows us to infer likely DNAm values at the regulatory elements of the marker genes in cell-types where these genes are not expressed. In response to the reviewer’s point we have now added a new SuppFig4 (shown below for convenience), that illustrates this imputation scheme for an example immune-cell marker gene (HCLS1).*

*SI fig.S4: Example of imputing promoter DNAm at a marker gene. a) Density distribution of normalized expression values in the SCM2 database across all genes and samples (black line) with a 2-component Gamma-mixture model fit displayed in green (non-expressed state) and magenta (expressed state). b) An example of a gene (HCLS1) which exhibits strong anti-correlation between promoter DNAm and gene-expression as assessed in the SCM2 database. c) As b), but now plotting DNAm (y-axis) versus the probability of being expressed (E ) given the expression level X, i.e. p(E|X). This probability is obtained via Bayes theorem, as the posterior probability from the 2-component Gamma-mixture model fit in step-a). This plot allows imputation of DNAm at the gene’s promoter for samples not expressing the given gene, as indicated. d) Illustration of how the expression levels derived from the scRNA-Seq tissue-atlas are mapped to corresponding DNAm levels, where the imputation proceeds for the samples not expressing the given gene. In the case of HCLS1, an immune-cell marker gene, no expression is associated with relatively high promoter methylation.*

Comment2b: I think what I was expecting to see in this section is that the imputation model would be some sort of beta-regression model, modeling DNA methylation beta-values (or average/median thereof) as the dependent variable, against expression as the independent variable. Under a Bayesian framework, one could assume weakly informative priors for the mean and dispersion parameter and use MCMC or Gibbs sampling to draw from the posterior distribution. This model could then be applied to the gene expression marker matrix in step 1 to obtain estimates of DNAm (e.g., mean of the posterior predictive distribution) to be used in deconvolution.

*Response: We appreciate the reviewer’s suggestion, which is an approach that could be explored in future studies. To us it is unclear however whether this may lead to improved inference. The relation between DNAm and gene expression is complex, even at promoter regions. From previous experience analyzing matched DNAm and gene expression data, it is clear that the relation is “bi-phasic” with methylated promoters generally (i.e 70-80% of the time) leading to gene silencing but with unmethylated promoters being uninformative of gene expression (repression can still happen to specific histone marks). Our imputation strategy reflects this in that we only seek to impute DNAm at promoter regions of marker genes that are not expressed in the given cell-types of the scRNA-Seq reference matrix. This is because their repression could be due to either promoter DNAm or histone marks. In case a gene is not expressed in some samples of the SCM2/RMAP databases, and there is a strong inverse association between DNAm and gene expression, then DNAm can be imputed. If however a marker gene exhibits a strong inverse association, but there is no sample in the SCM2/RMAP database where it is not expressed, we then need to fit a logistic regression to impute by extrapolation. We think that the choice of a logistic regression is also appropriate, as the beta-value itself can be interpreted as a probability of methylation.*

**Second round of review**

**Reviewer 1**

The authors have diligently addressed all reviewer comments and I appreciate the efforts that went into generating the additional supplemental figures that clarify these points.

**Reviewer 2**

The authors have adequately addressed my comments/suggestions. I have no further comments at this point.
